# Characterization of glutathione transferases involved in the pathogenicity of *Alternaria brassicicola*

**DOI:** 10.1186/s12866-015-0462-0

**Published:** 2015-06-18

**Authors:** Benoit Calmes, Mélanie Morel-Rouhier, Nelly Bataillé-Simoneau, Eric Gelhaye, Thomas Guillemette, Philippe Simoneau

**Affiliations:** Université d’Angers, UMR 1345 IRHS, SFR 4207 QUASAV, 2 Bd Lavoisier, Angers cedex, F-49045 France; INRA, UMR 1345 IRHS, 42 rue Georges Morel, Beaucouzé Cedex, F-49071 France; Agrocampus-Ouest, UMR 1345 IRHS, 2 rue le Nôtre, Angers cedex, F-49045 France; Université de Lorraine, UMR1136 Interactions Arbres-Microorganismes, Vandoeuvre-lès, F-54500 Nancy, France; INRA, UMR1136 Interactions Arbres-Microorganismes, F-54280 Champenoux, France

**Keywords:** Glutathione transferases, Necrotrophic fungi, Pathogenesis

## Abstract

**Background:**

Glutathione transferases (GSTs) represent an extended family of multifunctional proteins involved in detoxification processes and tolerance to oxidative stress. We thus anticipated that some GSTs could play an essential role in the protection of fungal necrotrophs against plant-derived toxic metabolites and reactive oxygen species that accumulate at the host-pathogen interface during infection.

**Results:**

Mining the genome of the necrotrophic *Brassica* pathogen *Alternaria brassicicola* for glutathione transferase revealed 23 sequences, 17 of which could be clustered into the main classes previously defined for fungal GSTs and six were ‘orphans’. Five isothiocyanate-inducible GSTs from five different classes were more thoroughly investigated. Analysis of their catalytic properties revealed that two GSTs, belonging to the GSTFuA and GTT1 classes, exhibited GSH transferase activity with isothiocyanates (ITC) and peroxidase activity with cumene hydroperoxide, respectively. Mutant deficient for these two GSTs were however neither more susceptible to ITC nor less aggressive than the wild-type parental strain. By contrast mutants deficient for two other GSTs, belonging to the Ure2pB and GSTO classes, were distinguished by their hyper-susceptibility to ITC and low aggressiveness against *Brassica oleracea.* In particular AbGSTO1 could participate in cell tolerance to ITC due to its glutathione-dependent thioltransferase activity. The fifth ITC-inducible GST belonged to the MAPEG class and although it was not possible to produce the soluble active form of this protein in a bacterial expression system, the corresponding deficient mutant failed to develop normal symptoms on host plant tissues.

**Conclusions:**

Among the five ITC-inducible GSTs analyzed in this study, three were found essential for full aggressiveness of *A. brassicicola* on host plant. This, to our knowledge is the first evidence that GSTs might be essential virulence factors for fungal necrotrophs.

**Electronic supplementary material:**

The online version of this article (doi:10.1186/s12866-015-0462-0) contains supplementary material, which is available to authorized users.

## Background

*Alternaria brassicicola* is the causative agent of black spot disease in a wide range of Brassicaceae crops. The necrotrophic behavior of this fungus exposes it to several plant defense compounds such as phytoanticipins and phytoalexins [1, 2] during host colonization. Brassicaceae phytoanticipins are represented by glucosinolates that are hydrolyzed by myrosinase when the plant tissues are damaged during necrotrophic colonization. Brassicaceae contain a large variety of glucosinolates, each of which form different isothiocyanates (ITCs) when hydrolyzed such as allyl-ITC (Al-ITC), benzyl-ITC (Bz-ITC) or phenylethyl-ITC (Ph-ITC) [3]. ITCs, which are the major breakdown compounds of glucosinolates [4], have been shown to exert their toxicity towards *A. brassicicola* by oxidative stress generation [5].

Glutathione transferases (GSTs) are a superfamily of proteins which are found widespread in animals, plants, fungi and bacteria. GSTs usually catalyze glutathione (GSH) transfer onto hydrophobic molecules (glutathionylation activity), or GSH removal from specific substrates (deglutathionylation) [6]. The varieties of electrophilic compounds, which can be conjugated to GSH by GSTs include aliphatic and aromatic halogen compounds, peroxides and epoxides, α, β-unsaturated and low molecular weight proteins. In particular, it has been shown that GSTs are able to conjugate GSH to ITC in human, *Arabidopsis thaliana* and *Phanerochaete chrysosporium* [7–9]. In the same vein, GSTs play a major role in insect adaptation to plant secondary compounds, such as glucosinolates (GLS) and ITCs in the polyphagous aphid species *Myzus persicae* [10]. In addition, ITCs specifically induce GSTs in *Caenorhabditis elegans,* providing oxidative stress tolerance [11]. Concerning fungal pathosystems, induction of GST encoding genes and increased transferase activity were reported in *Sclerotinia sclerotiorum* during *Brassica napus* infection and in the presence of ITC [12]. Moreover, some GSTs have also been identified as ligandin proteins that selectively bind organic anions such as tetrapyrroles in mammals and plants [13, 14]. This ligandin property has been defined as the capacity of the protein to bind non-substrate ligands [13]. In plants, it could be involved in intracellular transport of hydrophobic compounds such as pigments, and in temporary storage of phytohormones [15, 16]. In fungi, this ligandin property has been described for members of the GSTFuA class in *P. chrysosporium* [17]. These enzymes are able to bind wood-derived molecules and could participate in the intracellular transport and further elimination of these compounds, which could be toxic for the cells. Several GSTs also play a direct role in the antioxidant response through their peroxidase activity, which reduces endogenous or exogenous hydrogen peroxides or fatty acid peroxides [18, 19]. GSTs can usually accept various substrates. This functional property allows them to detoxify a wide range of endogenous and environmental chemicals and is part of their evolution in response to selective pressure. In *Saccharomyces pombe* and *Aspergillus fumigatus,* GSTs are involved in the oxidative stress response. Their gene expression is induced by hydrogen peroxide (H_2_O_2_). The promoters contain multiple copies of the stress response element (STRE) consensus region and the binding site of the Yap1 transcription factor, known to modulate the adaptive response to oxidative stress or cytotoxic agents [20]. In yeast, the GTT1 gene promoter contains specific regions of the response to xenobiotics [21] but GST regulation is usually the result of post-transcriptional modifications [22].

The pathogenicity of *A. brassicicola* could be partly related to its ability to protect itself against Brassicaceae defenses compounds including ITC [23–25]. The results obtained by [5, 26] showed that at least six genes encoding GST in *A. brassicicola*, were up-regulated upon exposure to ITC. One of these GSTs, named AbGST1, which is also up-regulated during interaction with the host plant has been the focus of a more detailed study [26]. *AbGST1* transcription was found to be significantly enhanced by heavy metals and 1-chloro-2,4-dinitrobenzene (CDNB) and the recombinant protein exhibited high glutathione transferase activity with allyl and benzyl ITC as substrate as compared to CDNB. In the present study, we functionally characterized the other five ITC-inducible GSTs as well as the phenotype of mutants deficient for these enzymes. Our results indicate that three of these GSTs, belonging to the GSTO, Ure2pB and MAPEG classes, may contribute to pathogenicity probably by protecting the fungus against the oxidative stress generated by host plant defense compounds.

## Results

### Phylogenetic analysis of GST- coding sequences from *A. brassicicola*

**Fig. 1:**
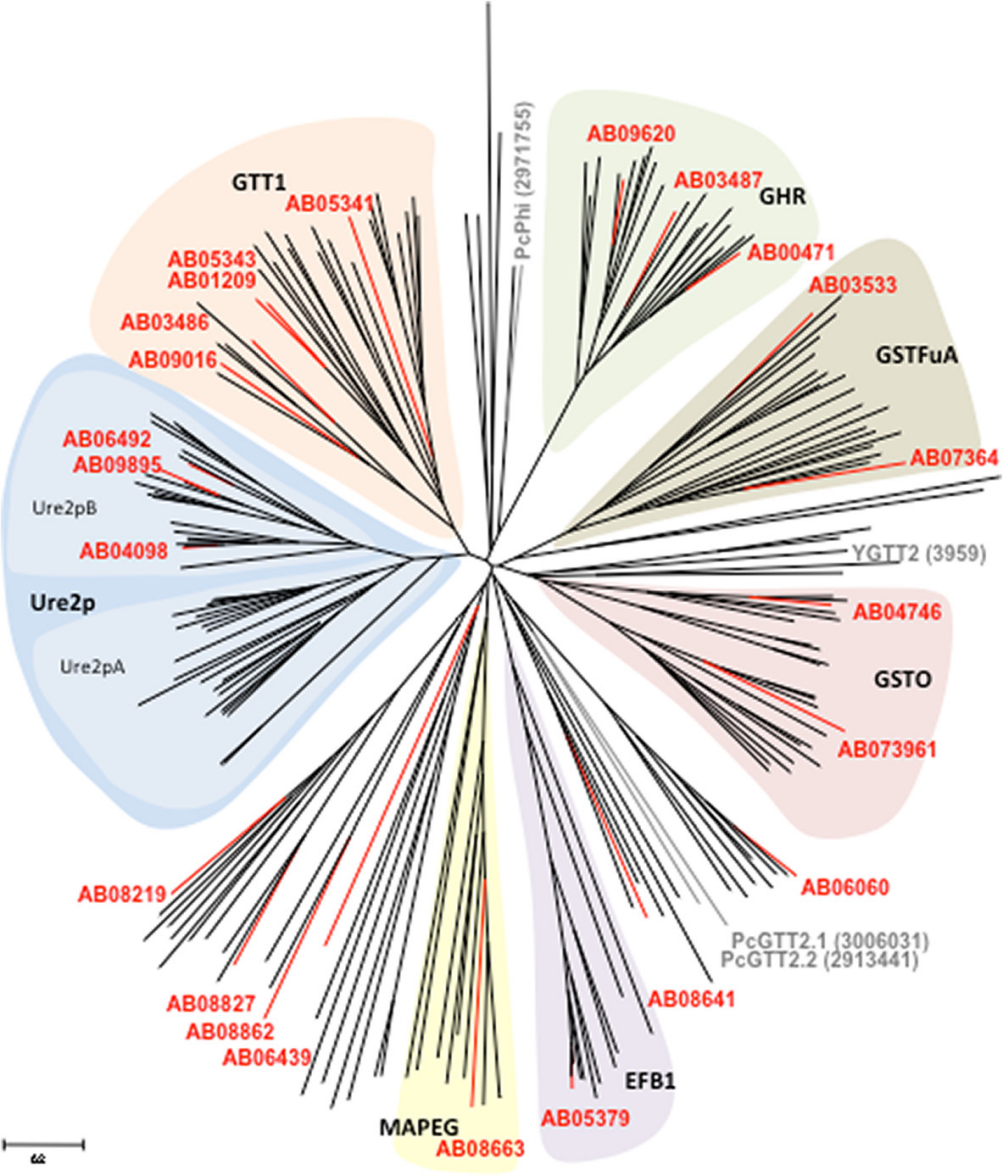
Phylogenetic tree of ascomycete glutathione transferases. Sequences from the basidiomycete *Phanerochaete chrysosporium* were added to allow GST classification into the following classes: Ure2p, GSTFuA, Omega (GSTO), Glutathionyl Hydroquinone Reductase (GHR), EFBγ, GTT1 and MAPEG. The sequences (Additional file 3) were retrieved from genomes of *Saccharomyces cerevisiae, Phanerochaete chrysosporium (Pc), Alternaria brassicicola*, *Botrytis cinerea*, *Leptosphaeria maculans*, *Mycosphaerella figensis*, *Mycosphaerella graminicola*, *Neurospora crassa* and *Stagonospora nodorum* available at the Joint Genome Institute (http://www.jgi.doe.gov/). Sequence alignments were done by CLUSTALW and the tree was constructed with the Neighbor joining method. The scale marker represents 0.2 substitutions per residue. Data available from the Dryad Digital Repository: http://dx.doi.org/10.5061/dryad.19f18

Searching the *A. brassicicola* genome (http://genome.jgi-psf.org/Altbr1/Altbr1.home.html) with glutathione transferase as keyword generated 25 entries. Sequence analyses revealed that 23 of them might correspond to true GSTs. A phylogenetic analysis focusing on GSTs from 8 Ascomycetes species and 1 Basidiomycete fungus allowed the clustering of most sequences into the main previously defined classes [27–29] (Fig. 1). *A. brassicicola* clearly exhibited 3 Ure2pB sequences but no Ure2pA, 1 EFBγ, 5 GTT1, 3 GHR, 2 GSTO, 2 GSTFuA and 1 MAPEG sequences. However, attribution of the other six sequences to previously identified groups was not clear. In particular, two sequences clustered close to *P. chrysosporium* GTT2, but their sequences contained domains related to beta or sigma GSTs that are usually found in bacteria and humans, respectively. Moreover, they were not related to the yeast GTT2 isoform. These sequences have thus not yet been classified. No member of the newly described phi class [30] was found.

In a previous study, five of these GST coding genes have been found up-regulated upon exposure to ITC [5]. Based on the present phylogenetic analysis, they were renamed as follows: AbGTT1.2 (AB05243.1), AbGSTO1 (AB04746.1), AbMAPEG1 (AB08663.1), AbUre2pB1 (AB09895.1) and AbGSTFuA1 (AB07364.1) corresponding to ITC-induced ESTs A1F1, A2C1, A2H5, A2C10 and A4D12, respectively [5] (Table 1). Similarly, the previously characterized AbGST1 from *A. brassicicola* [26] was renamed AbGTT1.1 (AB05341.1).

**Table 1:** Correspondence between EST, protein accession numbers and AbGST names derived from the phylogenetic analysis

EST	Protein	GST name
A1F1	AB05243.1	AbGTT1.2
A2C1	AB04746.1	AbGSTO1
A2H5	AB08663.1	AbMAPEG1
A2C10	AB09895.1	AbUre2pB1
A4D12	AB07364.1	AbGSTFuA1

### Enzymatic activities of selected AbGST proteins

Recombinant AbGTT1.2, AbUre2pB1, AbGSTFuA1 and AbGSTO1 proteins were produced in *Escherichia coli* and purified. Despite several attempts to optimize the production parameters, AbMAPEG1 was always found in the insoluble fraction and could thus not be purified in its active form. The enzymatic activities of the recombinant proteins were determined using CDNB as a classical substrate for glutathione transferase assay. Various ITCs were also used (Al-ITC, Bz-ITC and Ph-ITC) and peroxidase activities were tested using H_2_O_2_ and cumene hydroperoxide (Cu-OOH) (Table 2). Finally deglutathionylation activity was assayed using β-ME-SG. AbGTT1.2 and AbUre2pB1 exhibited both GSH transferase with CDNB and peroxidase activity with Cu-OOH. No activity was detected with H_2_O_2_. AbGSTFuA1 was found to be the most active protein in our tests, exhibiting significant GSH transferase activities with all ITCs. AbGSTO1 exhibited a deglutathionylating activity with β-ME-SG. These enzymatic profiles are in accordance with those identified within each class for other fungal species except for Ure2pB1 [9, 27, 29, 31, 32]. The Ure2p class was split into two subclasses (Ure2pA and Ure2pB). AbUre2pB1 clustered within the Ure2pB subclass, however it exhibited a glutathionylation activity like the yeast isoform, which belongs to the Ure2pA subclass, rather than the deglutathionylation activity previously measured for Ure2pB in *P. chrysposporium* and *E. coli* [33, 34].

**Table 2:** Kinetic parameters of AbGSTs in enzymatic assays

	AbGTT1.2	AbGSTO1	AbUre2pB1	AbGSTFuA1
***K***_**m**_ **(mM)**				
CDNB	1.82 ± 0.33	ND	5.01 ± 0.40	0.40 ± 0.02
Al-ITC	ND	ND	ND	0.12 ± 0.02
Bz-ITC	ND	ND	ND	0.18 ± 0.03
Ph-ITC	ND	ND	ND	0.11 ± 0.02
Cu-OOH	0.12 ± 0.04	ND	0.77 ± 0.06	ND
β-ME-SG	ND	1.12 ± 0.02	ND	ND
GSH	1.33 ± 0.10	0.45 ± 0.13	0.87 ± 0.02	0.50 ± 0.0004
***k***_**cat**_ **(s** ^**-1**^ **)**				
CDNB	0.10 ± 0.05	ND	1.50 ± 0.08	38.18 ± 6.10
Al-ITC	ND	ND	ND	76.80 ± 11.64
Bz-ITC	ND	ND	ND	11.81 ± 1.82
Ph-ITC	ND	ND	ND	20.54 ± 2.96
Cu-OOH	6.82 ± 1.18	ND	0.20 ± 0.01	ND
β-ME-SG	ND	49.00 ± 9.40	ND	ND

The apparent *K*_m_ values for all compounds were determined using a 0.1-50 mM concentration range in the presence of 3 mM GSH. The *K*_m_ value for GSH was determined with 2 mM CDNB and a 0.01 to 10 mM GSH concentration range. The apparent *K*_m_ and *k*_cat_ values were calculated by nonlinear regression using the Michaelis-Menten equation (r2 > 0.99). Data are represented as mean ± S.D. (n ± 3)

*ND* Not detectable

### Gene expression of GSTs during plant infection and in response to oxidative stress

**Fig. 2:**
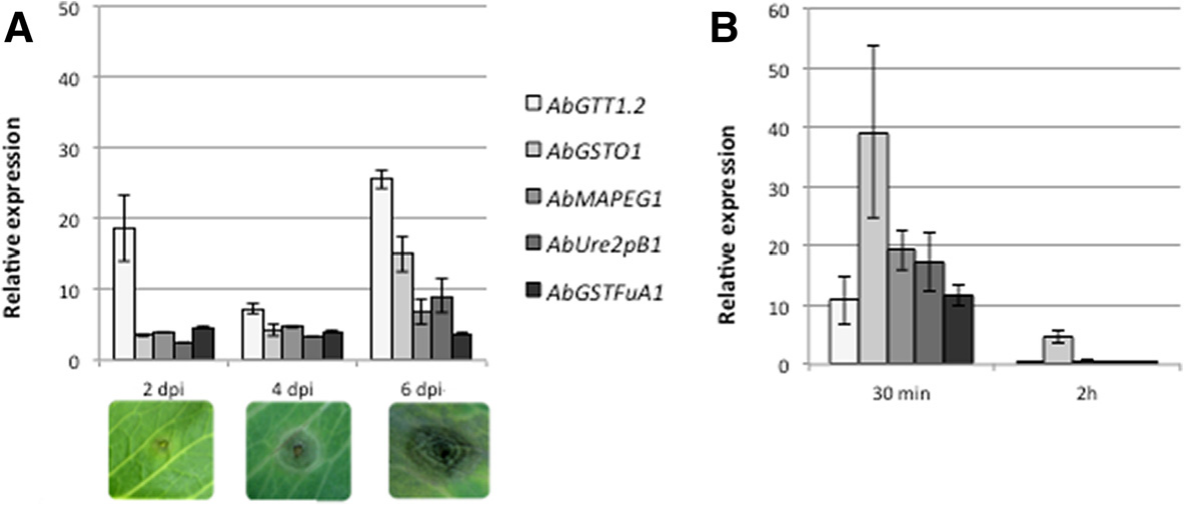
Quantitative RT-PCR results for the expression of selected GST genes. Gene expression was measured either *in planta* at 2, 4 and 6 day post inoculation (dpi) (**a**) or *in vitro* in the presence of H_2_O_2_ (2.5 mM) for 30 min and 2 h (**b**). For each gene, expression induction is represented as a ratio of its relative expression (studied gene transcript abundance/actin and tubulin transcript abundance) in each inductive condition to its relative expression in the corresponding control. The data are the mean of three repetitions

Gene expression of all the selected GSTs was previously shown to be ITC-inducible. Their expression was therefore checked during *in planta* interaction with *Brassica oleracea* (Fig. 2a). Genes encoding the five GSTs were up-regulated compared to the control (*in vitro* development conditions). *AbGTT1.2* gene was most strongly and quickly (2 dpi, i.e. when the necrotic area around the inoculation point was barely visible) induced during the interaction. The maximum expression level was reached at 6 dpi (i.e. extending necrosis with fungal conidia formed on the surface of infected tissues) for most of the GSTs. Peak of expression was particularly marked for the genes encoding AbGTT1.2 and AbGSTO1. Overall, the transcription induction of the five selected genes occurred very rapidly during infection and remained high throughout the interaction. As ITCs have been found to generate oxidative stress in fungi [5], expression of the five GST genes was also recorded in fungal cultures supplemented with H_2_O_2_ (Fig. 2b). Strong up-regulation of all the selected *AbGST* genes was observed after 30 min exposure and then the transcripts returned to their basal level after 2 h.

### Pathogenic behavior of GST-deficient mutants *in planta*

**Fig. 3:**
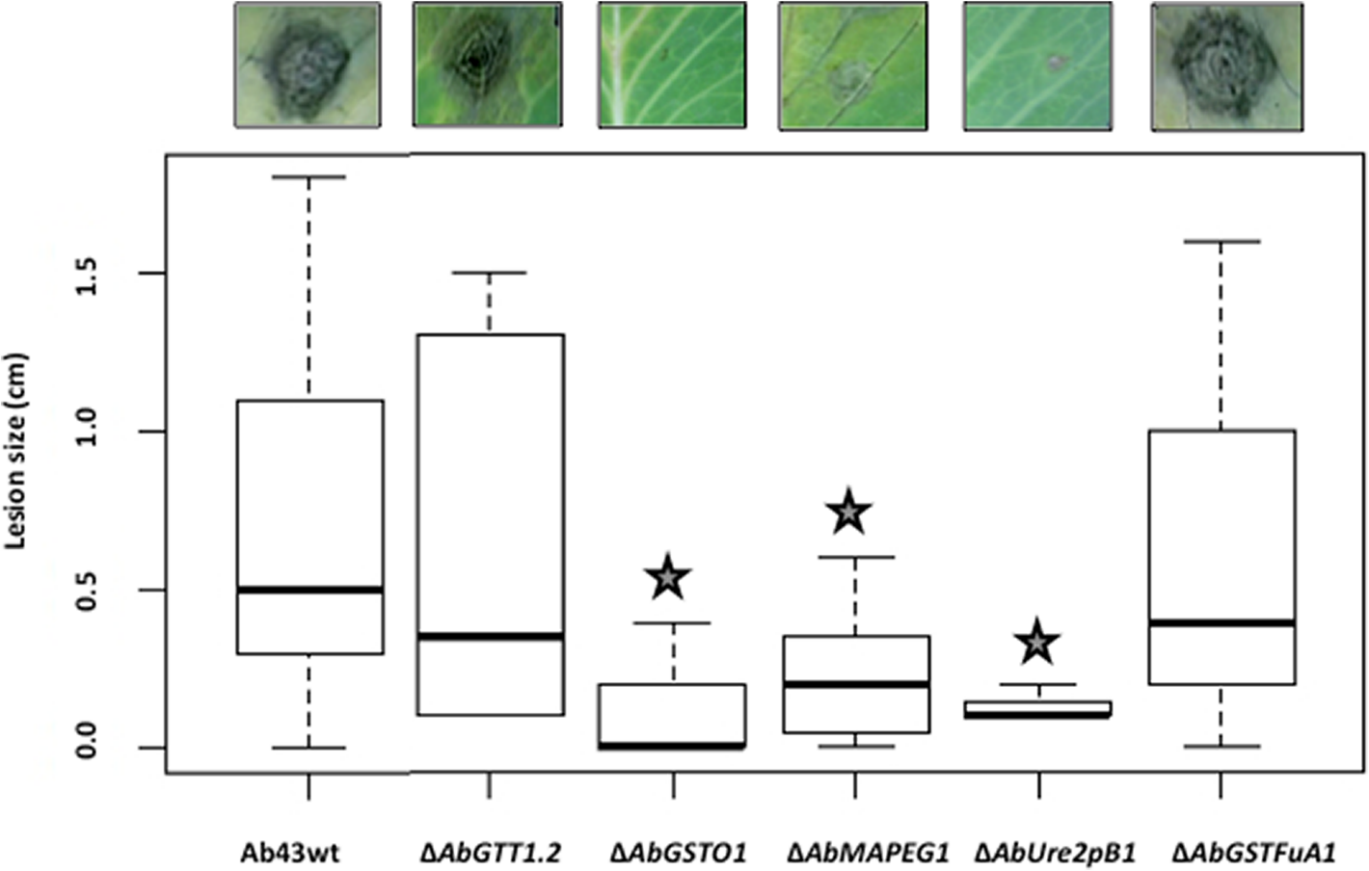
Pathogenic behaviour of GST-deficient mutants. *B. oleracea* leaves were inoculated with 5 μL drops of conidia suspension (10^4^ conidia/mL in water). Transformants were inoculated on the right part of the central vein and compared on the same leaf with the parental strain (inoculated on the left part of the central vein). Percentages of aggressiveness with respect to the wild-type strain were calculated at 6 dpi. Stars indicate a significant difference with respect to the wild-type aggressiveness (100 %) using the Student test (*P* < 0.01)

Virulence and aggressiveness of the GST-deficient mutants were evaluated on *B. oleracea*. Six days after inoculation of a highly concentrated conidia suspension (10^5^ conidia per ml), usual symptoms were observed for the wild-type and mutant genotypes (data not shown). At high parasitic pressure, the loss of one of the five-selected AbGSTs did not affect the virulence of *A. brassicicola*. The analysis of symptoms under lower applied parasitic pressure (10^4^ conidia per ml) revealed differences in aggressiveness between the tested genotypes (Fig. 3). Δ*AbGTT1.2* and Δ*AbGSTFuA1* mutants showed no significant difference compared to the wild-type when the inoculum was at low concentration. For ∆*AbGSTO1*, ∆*AbMAPEG1* and ∆*AbUre2pB1* a significant decrease in aggressiveness compared to the wild-type strain was observed at 6 dpi. The necrotic area decreased by approximately 70 % for these mutants.

### Susceptibility of GST-deficient mutants to isothiocyanates

**Fig. 4:**
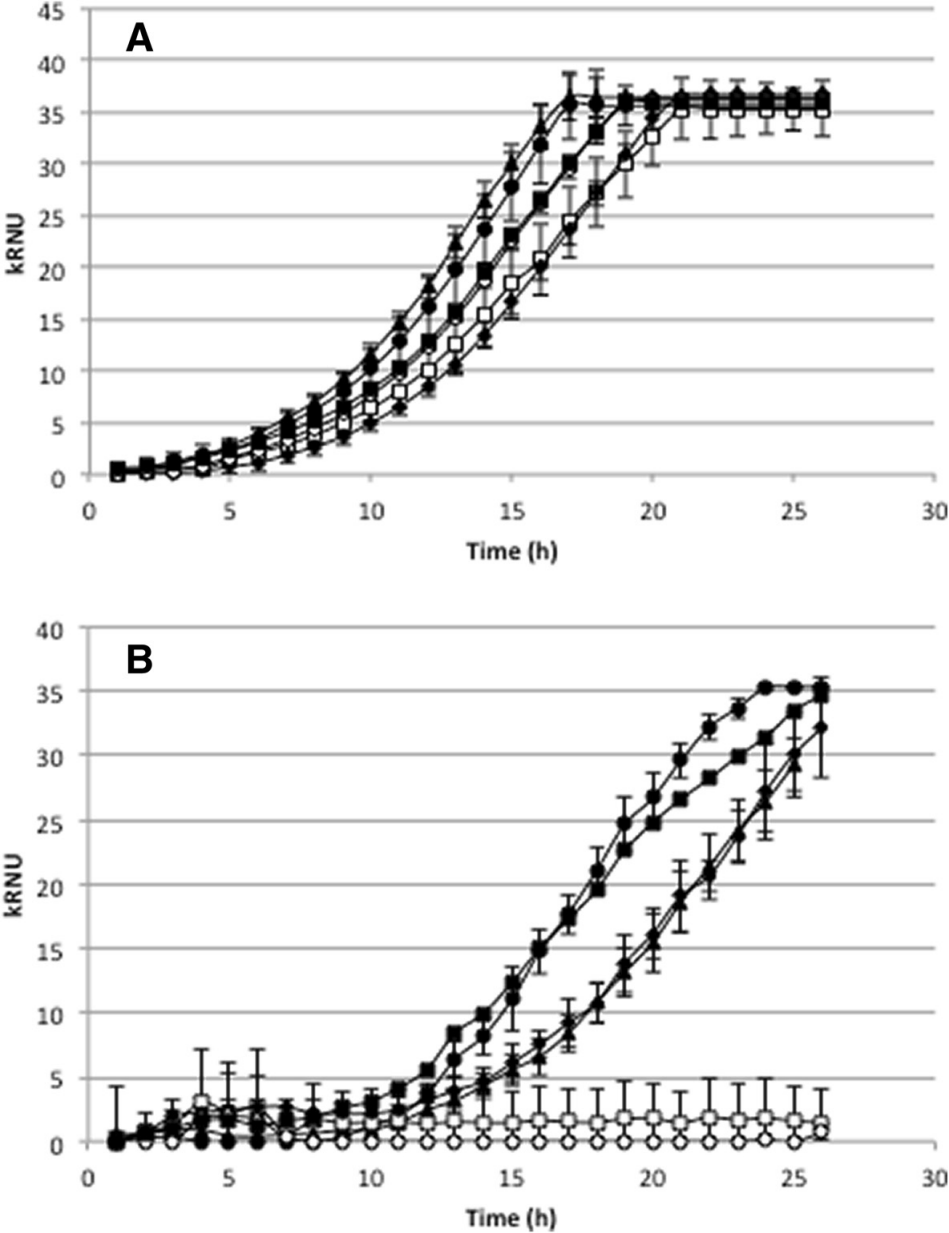
Nephelometric monitoring of growth of the wild-type strain and AbGST deficient mutants. Conidia from the wild-type and mutants were used to inoculate microplate wells containing standard PDB medium (**a**) or supplemented with Ph-ITC (**b**). Growth was automatically recorded for 25 h at 25 °C using a nephelometric reader. Each genotype was analyzed in triplicate and the experiments were repeated three times per growth condition. Black circles: wild-type strain; black triangles: *∆AbGTT1.2*; white squares: *∆AbGSTO1*; black diamonds: *∆AbMAPEG1*; white circles: *∆AbUre2pB1*; black squares: *∆AbGSTFuA1*

The effects of *AbGST* inactivation in *A. brassicicola* on conidia germination and initial mycelium growth in the presence of ITCs were examined. An analysis of the nephelometric growth curves (Fig. 4a) revealed that under control condition (PDB medium), no significant phenotypic difference in conidia germination (based on the lag time parameter) or mycelium growth (based on the maximum growth rate parameter) was detected in any of the tested mutants as compared to the wild-type. In contrast, mutants deficient for AbGSTO1 and for AbUre2pB1 showed an increased susceptibility to ITC compared to the wild type (Fig. 4b). In comparison, ∆*AbGSTFuA1* behaved like the wild-type strain and ∆*AbGTT1.2*, ∆*AbMAPEG1* had an intermediate phenotype.

## Discussion

In *A. brassicicola*, the first characterized GST, initially called AbGST1 [26] and renamed AbGTT1.1 in the present study as it belongs to the GTT1 family, was shown to possess conjugation activity of Al-ITC or CDNB to glutathione. A more thorough examination of the *A. brassicicola* proteome revealed 22 more putative GSTs that could be classified into 7 families according to the proposed classification. Investigation of the GST content in several fungal genomes revealed a huge diversity in terms of GST number ranging from 6 in *S. cerevisiae* to more than 40 in saprophytic Basidiomycetes [30]. However, the number of GSTs encoded by the *A. brassicicola* genome was comparable to that found in other fungal necrotrophs such as *Botrytis cinerea* (24), *Stagonospora nodorum* (23), or hemibiotrophs such as *Mycosphaerella graminicola* (25), *Mycosphaerella fijensis* (28), *Leptosphaeria maculans* (23). Five of the *A. brassicicola* GSTs that were previously shown to be up-regulated during ITC exposure [5] were clustered into five different classes, i.e. GTT1, GST Omega, GSTFuA, Ure2p and MAPEG.

Detailed analysis of the catalytic properties of these ITC-inducible GSTs revealed that, besides AbGTT1.1, another GST, called AbGSTFuA1, was able to conjugate ITC to glutathione. Members of the fungus-specific GSTFuA class have been described as having ligandin properties with various small aromatic compounds and GSH transferase activity with phenetyl-ITC [9]. This GST therefore appears to be a good candidate in protection mechanisms of *A. brassicicola* against host fungitoxic metabolites. However the mutant strain deficient for this enzyme was neither more susceptible to ITC nor less aggressive on *Brassica* than the wild-type and thus did not appear to be crucial for *A. brassicicola* pathogenicity. This could be due to a putative functional redundancy with the second isoform identified in the *A. brassicicola* genome. Otherwise it could be due to the fact that, in presence of high ITC concentrations, the efficient conjugation activity of AbGSTFuA1 could result in intracellular GSH depletion, leading to overall cellular oxidative stress. Indeed, Zhang [35] reported that ITCs are presumed to penetrate human and animal cells by diffusion but once inside the cells they rapidly conjugated *via* their -N = C = S group with intracellular GSH. Such conjugation, which takes place spontaneously but is enhanced by GST, may explain the rapid accumulation of ITC within the cells (up to 100-fold the extracellular concentration) and the rapid and marked depletion of GSH observed after ITC exposure [36, 37].

AbGTT1.2 is a second member of the GTT1 class of ITC-inducible GST in *A. brasiscicola*. However, unlike AbGTT1.1, AbGTT1.2 was able to conjugate glutathione to CDNB but was not found to accept ITCs as substrate and had a highly efficient peroxidase activity against cumene hydroperoxide. In *S. cerevisiae*, GTT1 was shown to catalyse the reduction of hydroperoxides [38] and to be involved in xenobiotic detoxification [39]. Strong *in planta* AbGTT1.2 gene induction was observed during fungal infection, but the *∆AbGTT1.2* mutant did not show reduced aggressiveness on its host plant or increased susceptibility to ITC compared to the wild-type. As five members of the GTT1 class were found in *A. brassicicola*, the absence of a marked phenotype for the AbGTT1.2-deficient mutant suggested that these GSTs have partial functional redundancies in this fungal pathogen.

By contrast with the two above-mentioned GST-deficient mutants, ∆*AbMAPEG1*, ∆*AbUre2pB1* and ∆*AbGSTO1* mutants were highly impaired in their pathogenicity, with a severe reduction in their ability to form extended necrosis on host tissues. The two latter mutants were also found to be much more susceptible than the wild-type to ITC exposure. AbGSTO1 belongs to the Omega family, a member of the cysteine-containing GSTs superfamily that is widespread in several kingdoms and phyla [40]. The conserved cysteine residue in their active site modifies their enzymatic properties as they do not catalyze conventional conjugation reactions but instead have glutathione-dependent thioltransferase activity like many other cysteine-containing GSTs [41]. In line with its apparent protective role against ITC toxicity, AbGSTO1 protein may therefore be involved in the reduction of sulfur bonds formed after reaction between ITC and cysteine sulfhydryl groups of target cellular proteins, thus restoring their enzymatic functions. Indeed, in human cells, ITCs were found to covalently bind to multiple cysteine residues of different target proteins such as α and ß-tubulin [42], Toll-like receptor TLR4 [43] or cytochrome P450 enzymes [44].

AbUre2pB1, as noted with AbGTT1.2, was found to function as GST and peroxidase although the measured activities were low. However, contrary to the ∆*AbGTT1.2* mutant, the ∆*AbUre2pB1* mutant showed increased susceptibility to ITC and decreased aggressiveness compared to the wild-type strain. The first structural and biochemical characterization of a fungal Ure2pB GST (PcUre2pB1 from *Phanerochaete chrysosporium*) was recently published [33]. Unlike PcUre2pB1, AbUre2pB1 did not depict any activity in deglutathionylation tests with β-ME-SG. Based on our results, it is therefore difficult to come up with any convincing explanation concerning the role of this enzyme in protection against ITCs and in pathogenesis, but they raise interesting questions concerning the evolution of these enzymes. It should be noted that, besides small molecules, PcUre2pB1 was also shown to be involved in glutathionylation/deglutathionylation of proteins, particularly to interact with a GST from the omega class of *P. chrysosporium,* PcGSTO3 [33]. As AbGSTO1 and AbUre2pB1 have comparable expression patterns and mutants deficient for these two GSTs have similar phenotypes, it would be interesting to check whether these two proteins could also interact and cooperate to protect the fungal cell against ITC toxicity. We have not been able to produce the soluble active form of AbMAPEG1 in a bacterial expression system. In fact, active recombinant forms of eukaryotic members of these membrane-associated GSTs have mainly been obtained in yeast or baculovirus/insect expression systems [45, 46]. However, careful inspection of the protein sequences derived from AB08663.1 (corresponding to the A2H5 ITC-induced EST, [5]) revealed the presence of a sequence pattern similar to that described by Bresell *et al.* [45] for the MGST3 sub-family of microsomal glutathione transferase (MAPEG) and a typical MAPEG hydrophobicity profile (Additional file 1). This protein has been shown to exhibit peroxidase activity and the corresponding gene is up-regulated in *P. chrysosporium* in the presence of toxic oak extracts, suggesting a putative role in the oxidative stress response [47]. The phenotype of the mutant defective for AbMAPEG1 suggested that this protein had no function in ITC detoxication while being important for full pathogenicity. Examination of microarray data from *A. brassicicola* exposed to brassicaceous indolic phytolexins (N’Guyen, unpublished) revealed that among the 23 GSTs, 6 were up-regulated in the presence of brassinin but of these only AbMAPEG1 belongs to the ITC-inducible set considered in the present study. This suggests that AbMAPEG1 could be involved in brassinin tolerance during host-plant colonization.

## Conclusions

Three of the ITC-inducible GSTs were found to be essential for the normal pathogenicity of *A. brassicicola*. So far functional characterization of fungal GSTs has mainly been performed with saprophytic model species such as *A. nidulans* and *S. cerevisiae* or wood-degrading species like *P. chrysosporium*. A mutant deficient for a GST (∆*BcGST1*) was obtained in *B. cinerea* but did not show any reduced virulence [48]. To the best of our knowledge this study therefore provided the first evidence that some GSTs play an essential role in the pathogenicity of a fungal necrotroph.

## Methods

### Fungal strains and growth conditions

The *A. brassicicola* wild-type strain Abra43 used in this study has previously been described [23, 24]. For routine culture*, A. brassicicola* was grown and maintained on potato dextrose agar (PDA). The method based on microscale liquid cultivation (from conidial suspensions) and automated nephelometric recording of growth, followed by extraction of relevant variables (lag time and growth rate), was described by [49]. To study the susceptibility of fungal strains to ITCs, allyl-ITC (Al-ITC), benzyl-ITC (Bz-ITC) or phenetyl-ITC (Ph-ITC), all purchased from Aldrich Chemical Co. (Milwaukee, WI), were diluted from stock solutions prepared in methanol at the final desired concentrations. Solvent concentrations in controls and assays did not exceed 1 % (v/v).

### RNA isolation and expression analysis by real-time quantitative PCR

Total RNA was prepared according to the TRIzol reagent protocol (Invitrogen). Additional cleanup and DNase treatment were performed using the Nucleospin RNA II kit (Macherey-Nagel) according to the manufacturer’s protocol. Complementary DNA was synthesized from 5 μg of total RNA using the reverse-transcription system [50 mM Tris-HCl pH 7.5, 75 mM KCl, 10 mM DTT, 3 mM MgCl_2_, 400 nM oligo(dT)_15_, 1 mM random hexamers, 0.5 mM dNTP, 200 units M-MLV reverse transcriptase, Promega]. The total volume was adjusted to 30 μl and the mixture was then incubated for 60 min at 42 °C. Aliquots of the resulting first-strand cDNA were used for real-time PCR amplification experiments using the ABI Prism 7000 sequence detection system (Applied Biosystems) and the SYBR green PCR master mix according to the manufacturer’s instructions. After 10 min denaturation at 95 °C, the reactions were cycled 40 times at 95 °C for 15 s and 60 °C for 1 min. The absence of contaminating genomic DNA in the RNA samples was checked by direct amplification of non-reverse transcribed samples. The synthesis of a single specific PCR product was verified by melting point analysis after the run. For each condition, all amplifications were performed in triplicate from two separate biological samples and the mean was determined for further calculations. The relative quantification analysis was performed using the comparative ∆∆Ct method as described by [50]. To evaluate the gene expression level, the results were normalized using Ct values obtained from actin cDNA amplifications run on the same plate.

### Generation of targeted gene replacement constructs and fungal transformation

The gene replacement cassettes were generated using the double-joint PCR procedure [51]. The selectable marker inserted in the PCR constructs corresponded to the *Hph* gene cassette (1436 bp) from pCB1636 [52] conferring resistance to hygromycin B. The sets of primers used to amplify the 5’ and 3’ flanking regions of each targeted gene are presented in the Additional file 2. The double-joint final PCR products were used to transform *A. brassicicola* protoplasts as described by [53]. The *A. brassicicola* wild-type Abra43 was used to obtain the hygromycin-resistant strains deficient for each GST. Potential transformants were prescreened by PCR with relevant primer combinations (Additional file 2) to confirm integration of the replacement cassette at the targeted locus. Two putative gene replacement mutants for each construct were further purified by three rounds of single-spore isolation and then confirmed by PCR.

### Infection assays

For plant infection assays on *Brassica oleracea* plants (var. Bartolo), 5 μL drops of *A. brassicicola* conidia suspension (10^5^, 10^4^ or 10^3^ conidia/mL in sterile water) were inoculated on leaves from 5 week-old plants. Inocula were symmetrically deposited on the left and right sides from the central vein. The plants were then maintained under saturating humidity (100 % relative humidity). Symptoms were observed and samples collected at 2, 4, 6 days post-inoculation (dpi) for *AbGst* expression analyses.

### Expression and purification of the recombinant proteins

GST coding sequences were amplified from the cDNAs obtained as described above, using the Phusion™ Hot Start High Fidelity DNA polymerase (Finnzymes) and relevant primer sets (Additional file 2). The PCR products were cloned into the *Nco*I and either *Bam*HI sites (AbGTT1.2, AbGSTO1, AbMAPEG1 and AbGSTFuA1) or *Xho*I (AbUre2pB1) of the pET-14b vector (Novagen) resulting in a construction devoid of an His-Tag. The recombinant plasmids were then used to transform the *Escherichia coli* strain BL21 (DE3) co-transformed by the chloramphenicol-resistant plasmid (pRARE) in order to provide the rare t-RNAs for AUA, AGG, AGA, CUA, CCC, and GGA. Cultures were progressively amplified up to 2 L in LB medium supplemented with ampicillin and kanamycin at 37 °C. Protein expression was induced at the exponential phase by adding 100 μM isopropyl β-D-thiogalactopyranoside (ITPG) for 4 h at 37 °C. The cultures were then centrifuged for 15 min at 4400 × *g*. The pellets were suspended in 30 mL of TE NaCl (30 mM Tris-HCl, pH 8.0, 1 mM EDTA, 200 mM NaCl) buffer. Cell lysis was performed on ice by sonication (2 × 2 min at 1 min intervals), and the soluble and insoluble fractions were separated by centrifugation for 30 min at 27,000 × *g* at 4 °C. The soluble fraction (supernatant) was then fractionated with ammonium sulfate in two steps, and the protein fraction precipitating between 40 and 80 % of the saturation contained the recombinant protein, as estimated by 15 % SDS-PAGE. The protein was purified by size exclusion chromatography after loading on an ACA44 (5 × 75 cm) column equilibrated in TE NaCl buffer. Fractions containing the protein were pooled, dialyzed by ultrafiltration to remove NaCl, and loaded onto a DEAE cellulose column (Sigma) in TE (30 mM Tris-HCl, pH 8.0, 1 mM EDTA) buffer. The proteins were eluted using a 0–0.4 M NaCl gradient. Finally, the fractions of interest were pooled, dialyzed, concentrated by ultrafiltration under nitrogen pressure (YM10 membrane; Amicon). Purity was checked by SDS-PAGE. Protein concentrations were determined spectrophotometrically using the specific molar extinction coefficient at 280 nm of each GST as calculated online (http://web.expasy.org/protparam/) using the ProtParam tool: 42 400 cm^-1^ M^-1^, 46 410 cm^-1^ M^-1^, 60 390 cm^-1^ M^-1^, 49 390 cm^-1^ M^-1^ for AbGTT1.2, AbGSTO1, AbUre2pB1 and AbGSTFuA1, respectively.

### Activity measurement

GSH transferase activity was spectrophotometrically assessed with Al-ITC, Bz-ITC and Ph-ITC prepared in methanol and 1-chloro-2,4-dinitrobenzene (CDNB) prepared in DMSO. The increased absorbance arising from the formation of the S-glutathionylated adduct was monitored at 274 nm for ITC and 340 nm for CDNB. Reactions with CDNB were performed in 100 mM phosphate buffer (pH 7.5), in the presence of GSH (5 mM) while the reaction with ITC was performed at pH 6.5 with an identical GSH concentration. Peroxidase activities were monitored as follows: 1 mM peroxide (hydrogen peroxide, and cumene hydroperoxide) in 30 mM Tris-HCl (pH 8.0), was incubated in the presence of 2 mM GSH, 200 μM NADPH, 0.5 IU glutathione reductase. The activity was monitored according to the decrease in absorbance at 340 nm arising from NADPH oxidation in this coupled enzyme assay system. Hydroxyethyldisulfide (HED) was incubated with GSH to allow the spontaneous formation of glutathionylated β-mercaptoethanol (β-ME-SG). Then, the β-ME-SG deglutathionylation test was monitored using the coupled system but by using 1 mM HED instead of the peroxides. The reactions were started by adding the purified enzyme (0.1 μM) and monitored with a Cary 50 UV-Visible spectrophotometer (VARIAN). Catalytic parameters were calculated using the GraphPad® software.

### Sequence analysis

Amino-acid sequence alignments were done by CLUSTALW and the tree was constructed with the neighbor joining method in MEGA 5.0 software [54]. Hydropathy curves were generated according to Kyte and Doolitle [55].

## Additional files

Additional file 1:
**Structural features of AbMAPEG1.** (A) Protein sequence of AB08663.1. Amino-acids in red are conserved in the specific pattern characteristic for MAPEG members belonging to the MGST3 subfamily [45]. Underlined residues are conserved in all members of the MAPEG family [46]. (B) Hydrophobicity plots according to [55] of the protein sequences from AB08663.1 (left) and human MGST3 (Swissprot identifier O14880) (right). Additional file 2:
**List of primers.**
Additional file 3:
**List of sequences used to generate the phylogenetic tree of GSTs.**

## Abbreviations

ß-ME-SG: glutathionylated β-mercaptoethanol
CDNB: 1-chloro-2,4-dinitrobenzene
Cu-OOH: Cumene peroxide
GSH: Glutathione
GST: Glutathione-S-transferase
HED: Hydroxyethyldisulfide
ITC: Isothiocyanate
