# Supplementary figures and images for: Characterization of glutathione transferases involved in the pathogenicity of Alternaria brassicicola
Source: BMC Microbiol. 2015 Jun 18;15:123. doi: 10.1186/s12866-015-0462-0 (PMC4470081; doi:10.1186/s12866-015-0462-0)

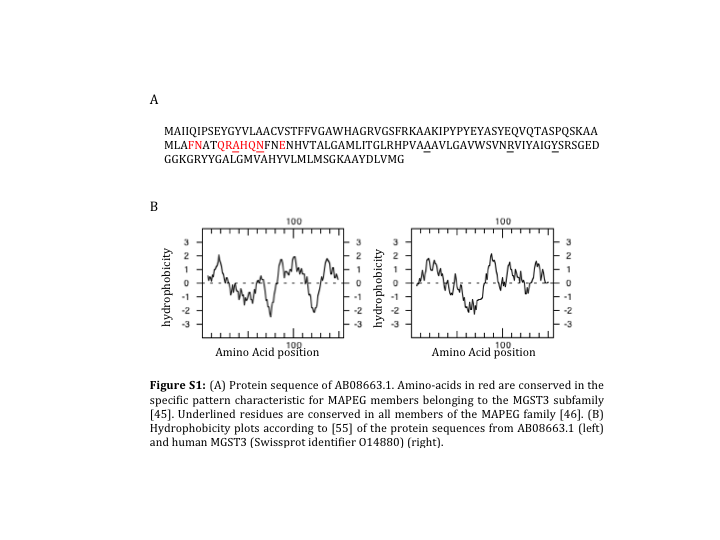

Supplement: Additional file 1: — Structural features of AbMAPEG1. (A) Protein sequence of AB08663.1. Amino-acids in red are conserved in the specific pattern characteristic for MAPEG members belonging to the MGST3 subfamily [45]. Underlined residues are conserved in all members of the MAPEG family [46]. (B) Hydrophobicity plots according to [55] of the protein sequences from AB08663.1 (left) and human MGST3 (Swissprot identifier O14880) (right). [file 12866_2015_462_MOESM1_ESM.tiff]
