# Supplementary material for: Characterization of glutathione transferases involved in the pathogenicity of Alternaria brassicicola
Source: BMC Microbiol. 2015 Jun 18;15:123. doi: 10.1186/s12866-015-0462-0 (PMC4470081; doi:10.1186/s12866-015-0462-0)
Supplement: Additional file 2: — List of primers. [file 12866_2015_462_MOESM2_ESM.docx]

**Additional file 2:** List of Primers

| AbGTT1.2 | FP1 | AATACCGCAATGTGCAAGGT |
| --- | --- | --- |
|  | RP1 | TCCTGTGTGAAATTGTTATCCGCTCCTCAATGCTCCCACTTTGT |
|  | FP2 | GTCGTGACTGGGAAAACCCTGGCG GCCCGAAACCCAGACTTATT |
|  | RP2 | GATCGTCTCGATGCTACGC |
|  | FN1 | TAGGAGGAGCATCCATACCG |
|  | RN1 | TGTCGAAAAGAAAGGCCAAG |
|  | FqPCR | AACTTCACAGCCGCTGACATCT |
|  | RqPCR | TGGTCGTAACGATGAAGACGG |
|  | Frp | ACCTGCCAGCCATGACCCTGATCGTACATCAC |
|  | Rrp | GGATCCTCAGTAGCTTATCATCGGCC |
| AbGSTO1 | FP1 | TGACGGCATAATTTGCTGAA |
|  | RP1 | TCCTGTGTGAAATTGTTATCCGCTGATGCTGGGTAGGAGGCTTA |
|  | FP2 | GTCGTGACTGGGAAAACCCTGGCGTTCGCTCATCAGCGTACATT |
|  | RP2 | GCGACGACAACCATACACAC |
|  | FN1 | GAAGACTGACCGACGAGCAT |
|  | RN1 | TCGTGGTTCGAGGTTTTAGG |
|  | FqPCR | TTGCTGAACCAAGTGTCGACG |
|  | RqPCR | TGACCCGAGCGAGAATCAACT |
|  | Frp | ACCTGCCAGCCATGGCTCAAGTCAATGGAAATG |
|  | Rrp | GGATCCTCACTTGGAAGCTGC |
| AbMAPEG1 | FP1 | TGGCCAACTTGACAATTCAG |
|  | RP1 | TCCTGTGTGAAATTGTTATCCGCTAGCCGTCTGTCTGCCTTTTA |
|  | FP2 | GTCGTGACTGGGAAAACCCTGGCGCTACATGTGGAAAGGCGTCA |
|  | RP2 | ACAGCCCTGGTTTCCTTTCT |
|  | FN1 | AAACCACTGTTCTCCCCACA |
|  | RN1 | AACTATGGAAGCACGCCAAG |
|  | FqPCR | AATCCAGAGCCGCAATGCTAG |
|  | RqPCR | AAGTTTTGGTGTGCGCGCT |
|  | Frp | CCATGGCGATTATCCAGATCCCC |
|  | Rrp | GGATCCTTATCCCATGACAAGATCGTAGG |
| AbUre2pB1 | FP1 | TCAAATTTGTGCACGTGGTT |
|  | RP1 | TCCTGTGTGAAATTGTTATCCGCTTTGCAGAGAGGGCTTGAAAT |
|  | FP2 | GTCGTGACTGGGAAAACCCTGGCGTCTTCCCAAGTGGAATTGCT |
|  | RP2 | GATCTGGAAGATGTGCGTGA |
|  | FN1 | CAAAAAGGGTATTGCGCTGT |
|  | RN1 | TTCTGAAGGATCGGGGTATG |
|  | FqPCR | TGGATGGAAGGCTAGCATTCTG |
|  | RqPCR | TTTCCGTAGGCATCCTTGAGC |
|  | Frp | CCATGGCCGACCAAGAACCCAC |
|  | Rrp | CTCGAGTTAAGGGCTGGAGTACTTGTAGC |
| AbGSTFuA1 | FP1 | ACGCCAAGGTGCTTATTGAG |
|  | RP1 | TCCTGTGTGAAATTGTTATCCGCTGAGTGTTGGGAGAGGACCAA |
|  | FP2 | GTCGTGACTGGGAAAACCCTGGCGCCATCTGGTAGGTGCCATTC |
|  | RP2 | CGTCGCCTTGCTTGTAAATA |
|  | FN1 | CGTTGGCATCCTTTCCTACT |
|  | RN1 | GAGCTCGAGAAACAGCATCC |
|  | FqPCR | CATTTCGTCAAGTGCATAAGCG |
|  | RqPCR | CGAGAGCCATAAGCTCCTTGAA |
|  | Frp | CCATGGCGTCTTCAAAAGTTGTTTTG |
|  | Rrp | GGATCCCTAATCTTCTCTCTCCAGCCAC |
| Hph | M13F | TCCTGTGTGAAATTGTTATCCGCT |
|  | M13R | CTCCTCGCCCTTGCTCACCAT |

For each target gene, primer sets FP1-RP1, FP2-RP2, FN1-RN1 were used to the generate by double-joint PCR the gene replacement cassettes carrying the *Hph* resistance gene amplified from pCB1636 with the primer set M13F-M13R. Primer sets FqPCR-RqPCR were used for quantification of *AbGst* genes by real-time PCR. Primer sets Frp-Rrp were used for amplification of full-length coding sequences (underlined nucleotides correspond to start codons)
