# Supplementary material for: Characterization of glutathione transferases involved in the pathogenicity of Alternaria brassicicola
Source: BMC Microbiol. 2015 Jun 18;15:123. doi: 10.1186/s12866-015-0462-0 (PMC4470081; doi:10.1186/s12866-015-0462-0)
Supplement: Additional file 3: — List of sequences used to generate the phylogenetic tree of GSTs. [file 12866_2015_462_MOESM3_ESM.docx]

>jgi|Phchr2|2971108|fgenesh1_pg.12_#_292=GSTO4

MSVPDVQIFPHATGEAAKTVEKHQAPQDLVFYSGWFCPYVQRTWIALEERNIPYQYVEVNPYKKEKHFLEINPKGLVPAIEYKGRALYESLILCEFLEETYPEHTPHLLPADTVERAYARIWIDFVTKNIVPNFMRLVQAQTTEKQDAARADLYRGLRTFAEKVRGPYFLGDDFSLVDVALAPWVVRDWVIAENRGYDRAAVGDVWKRYAEIVEQRDSIRKTSSERQHFTDIYGRYLRDEAQSEAAKAIREGRAIP*

>jgi|Phchr2|2982482|fgenesh1_kg.7_#_1005_#_Locus3358v2rpkm76.97=GHR1

MSFTTGSTLQHQEAQLQGAKDETVTQLEQRAAGGASHELQSDISKMKTEDDGSFKRKAASFRNWIQPNGDFTPEKGRYHLYVSYACPWATRTLIVRKLKGLEDFIGVTVVSPRMGSNGWPFANVDPFPAADSDPLNNAQHVKDLYLKVKPDYDGRFTVPVLWDKHTGTIVNNESSEIIRMFNTAFNHLLPEDKAKLDLYPESLRAKIDEVNDWVYDTVNNGVYKSGFASTQKAYEAAVIPLFESLDRLEKMLEGQDYLIGGQLTEADIRLFVTIVRFDPVYVTHFKCNLRTIRDGYPNLHRWMRKLYWGNPAFKDTCNFEHIKTHYFWSHTFINPHRIVPIGPIPDILPLDA*

>jgi|Phchr2|2624184|CE1624184_2627=GHR2

MTSLSASGSAIHITFLTNGLLDTLTRRFTSTPLRLLRPASYVQAPYISLRKANMSTGSTLPHQHTALQQANEQRLPARDPGQSDVSKMKMQADGSFDRKPSTFRHWISQDGPFKPEKGRYHLYVSYGCPWATRTLIVRQLKGLEDVISVSVVSPRMDANGWPFANVDPFPGADEDPLHGARHLKDLYLRVEPEHAGRFTVPLLWDKQQDTIVNNESSDIIRMFNTAFNGILPQDKAALDLYPESLRAKIDSLNDWVYNTVNNGVYKAGFAQSQKAYEDAVIPLFESLDRLEKILTGKDYLVGDQLTEADVRLFVTIVRFDVGYHTAFKCNIRSIRADYPALHRWLRRLYWTIPAFKDTCNFQHIKVGYFGLTMINPFKIVPVGPLPDILPL*

>jgi|Phchr2|3006031|fgenesh1_pm.11_#_400= GTT2.1

MSPKPVLYTFGLSVWSAVVELAALELGVEIESKIINVVAGENFSPEFLKINPAGTIPAIVAPDGKVYGNTVESLRYILSVAPKKVTPGHAEIIDKVHDDDHDPNFALLTARNEEELKKAAEGFPFTFVTNRQNALEKYSSSAEGAAFKDWYAEKLQKNGGLLKVYKGEVPATDFFAISTKLWENLTSYILHDLPQILPESGFIAGAEPGEDDYHVAAWLARVTWVTGGNKELDGYKALEKELHTDVPPKVVAYWQLWASRPSWSKVYAETLH*

>jgi|Phchr2|2934909|estExt_Genewise1.C_9_t20397= EFB1

MSLGTLYTVPQQAQGKRIRAAAAFAGLKVDLPESYVHFEDNKKPEFLAKFPHGKIPALDGADGFRVFETSAIARYVASLAPNSTLLPSDVKEAALVEQWISVADNEIGSYVGLINQLCRNIIPYSKSVHQTFTERAIRGLKTVEQHLATRTYLVTERITLADITLASVVQRAAAIILDAELRPQFPNVIRHAETIVNHPAMKEAFGPIEWCEKALQFIPPAKEKKEAKPAAEKPKAEKKPKKVEEDEEDDDLVPKEAPKEKNPLDLLPKSTFNLEDWKRAYSNKDTRGPGGSIEWFYQNFDKEGFSVWRVDFKYNNELTQTFMSSNQIGGFFNRLEASRKYLFGSMGVLGQTNDSIISGVLIARGQDIKPVVEVAPDFESYEYKKLDLENPEDKAFFEAALAWDLEIDGKKWVDGKNFK*

>jgi|Phchr2|2687275|CE1687275_16632=GSTFuA2

MAQPIVFYDIPSNERIKHSPWSPNTWKIRYALNYKGLKYKTEWVEYPDIAGVVQKLGGKPTEKTPDGRDHYTLPVIYDPNTKKVVEDSAAIAKYLDETYPDTPKLFPAGTDAFQAAFLDFAWPVLGFPVFMLVILDTANSLLPRSHDYFRSTREQKFGKKLEELATEEEWAKVEAGLAKLKGYLDANGKGNDLLLMGAQGGITYSDIQIASFFVWAKIIWGEGSEKWKRLISLHDGKWAQFYAQFTKFEQVDV*

>jgi|Phchr2|2959905|estExt_Genewise1Plus.C_12_t20365=GSTO3

MPIPDEKIFPHATGAAAKTVEQHQDPQELVFYAGWFCPFVQRTWIALEERGIPYQYKEVNPYKKEKHFLDINPKGLVPAIEYKGKALYESIILCEFLEDAYPNYKPKLLPEDPFERAYARIWLDYISKSIIPANFRLIQAQTPEKQQEALADFNKALKQFAEKIKGPYFLGEQFSLVDIAIVPWIVRDYIIAENRGFKREDIGSKWVEYASKLEKRDSVARTSSEHYAEIYGRYLRDEAQSEAAKATRAGRDIP*

>jgi|Phchr2|2913441|e_gw1.11.1113.1=GTT2.2

MSSTQPLPKASLYYYKNSIWASVPLLALEEKGYGADEVDLKEVDLSKGENFSPAYLRINNNATVPTLVVPLENTLEPEIESRYKAIKDTKAIVEFLDRSRSPQSRTHSTSTAPAPSLAPATIAFSSISNKIIELLHSEAGDPNALTFMNARNDAQLKALAAERKQMLIEKCDALDHLIEENKQADVKVSEKTVRFWEMKRVASSMILDVLEDGEKPTEELNDEAKGKRDEFFKAAAQAWAGLKDVLVQLSKEIIGPYTLGDQISIADLHLAAWLARIAKLSGGQASDDGNTIVQKIEEHIGDSFSLPKDFSVAEARRRAGLPATNIPPTERQARLAAFWDAIKERPSWKKIYADGLH*

>jgi|Phchr2|2908587|e_gw1.8.23.1=GSTFuA3

MSLEPIIFYDIPANEPRQMAWGPNTWKTRYVLNFKGLKYRTEWVEYPDIEAVCKQIGAPATEKKPDGRDHYTLPVIQDPNTKAVVADSDAIAKYLESTYPDTPRLFPEGTRAFQHAFYQLARPSVLMPIFNIVVARVWKLLRPRSQEYFRATREQMLGKKLEEIGSEDDWNALESGLARIKSSLEANGAGKDLLLMGDRVTFADLQLASLFIWLRVSAGEESEDWKRFLSLHEGKWAKFMQQFAAYEFVDV*

>jgi|Phchr2|3028385|gm1.6602_g=GSTFuA4

MADVITLYDIPGKSDQCKAWSPSTWKARFALNIKGIPYKTEWVEYPDIATVYEKHGITAKYTGDDGLPLYTLPVIYDPATRRTVAESSDIAKYLDRTYPNTRTLFPTGTAALHEAFAGAYTAVHKALYNLVCCAACYALTERSQAFYRTTREARLGKALEDVCTAQDWVDAEAAFGRLAGWLDANGAGADAEWVMGAGTLCFADVRVVSGLMWARTTLGEASAEWKRVCGWHGGRWARVVANFGPYAHVDL*

>jgi|Phchr2|6880|Phchr1.fgenesh1_pg.C_scaffold_12000064=GSTO7

MAQKEQITFYTHFYSPYCDRVHLALEEVKADYTVYTVDVMNKPKWYTEQINPVGKVSRALSPSAHLTLVQIPAITYGGPKVKPEEPSPESCKLRESLVILEFLADLFPEAGLLPTDPVLRAKARLFASDVDAHVFEGFKAYFFMREPASKLLDALDRFQQQLPARGFAVGDKWTLADMAAAPFLVRTYLLLEHDLGVYPAGEGPKTLALLRGERFARLNQYLADLRAQPSFKATWDEAAQVAIWKSNPMFKRE*

>jgi|Phchr2|2914013|e_gw1.12.474.1=GSTFuA5

MAQNLITLFDVPSTTPQPWAPNIWRIRFILNFKRLPYRTTWVELADVETTLRSLGAPPSSVRADGRPVYSLPVIVDPRQGQQPVVLSRADHIAEYLEATYPARAIFPEGARAVQSLFVHYVHEVLAKPLLPILVPLSHQRLPERSQAHFPAGVAVPPPLGAAEREHAWRAAKDQFAFLAALMDKNAADPACDGVVVMGRSVTYADFALCSVLIWIEQVSPRDGWARVRTWDNGRWDRLRAKCREYMDVY*

>jgi|Phchr2|2974058|fgenesh1_kg.1_#_815_#_Locus2995v1rpkm89.75=Ure2p1

MFSILRQASRLSPPPLLARTTLPAHRALSAMATNTDKPVVHYTAPTPNGWVPAILLEELKAVYGGPDYETVKMSIRDADIGKVHNQVKSDWFLKICPNGRIPAITHEGFPVFETSAILLYLAQHFDKENAFSRDPVKDPKGYSEELQWLFFAHGGIGPMQGQANHFNLYAPEKIPYAINRYLNESKRLYRVLDDRLKGREYILGTYGIADIKIFGWARIAPRTGLDLDEFPNVKAWVERIEKRPAVQAGINSCN*

>jgi|Phchr2|2977129|fgenesh1_kg.3_#_506_#_Locus6880v2rpkm1.53_PRE=Ure2p7

MSHGKQFTLYSHKGGPNGWKVAFVLEELGLTYETIYLDFQKNEQKSPEFLKLCPNGRIPAIVDHKNGDFVVWESNAIVQYLVDKYDKEHRLSVAPGTDEYYTQLQWLYFQASGQGPYYGQAGWFTFYHPEKVPSALERYRNEIKRVLGVLESVLSKQEWLVANRPTVADLSFIPWNVAAVTRLLENFDFEKEYPATAKWHKTLLERPAVKKVWEEREKVLARQ*

>jgi|Phchr2|2057875|CE1057875_1927=Ure2p6

MSHGKQFTLYTHKGGPNGWKVTIVLEELGLTYESIFLDFQKGEHKAPEYLKVNPNGRIPALIDHKNNDYTVWESNAIIQYLVDKYDKDRKVSVAPGTNEYYTQLQWLYFQASGQGPYYGQAAWFSVYHPEKVPSAIERYRNEIKRVLGVLESVLSKQEFLVDGKATVADFSFLPWNEGAAKFLLEGSQFEEEFPATAKWHKKLLERPAIAKVWEERAKVSAH*

>jgi|Phchr2|3020858|estExt_fgenesh1_pg.C_160152=Ure2p3

MSHGKHFTLFGHRRAPNAWRVPVILEELGLPYELVLLDFTKSEHKAPEYTRYNPNGRVPAIVDHQNGDVVLWESDAIIVYLVDHYDPTHTISVAGGVDKYFQLQWLFFQASGQGPPFGNAAWYALFSPEKFPPVLERFRAEVRRVLGVFESVLSTQEWLVGGKVTVADLSFVSWNNFVIPDAMEPGFNFDKEFPATAAWHKKLFERPNVQRVYAHRAALIAEQ*

>jgi|Phchr2|3031824|gm1.10041_g=Ure2p9

MSHGKQFTLFTHKLGPNGWKVAIVLEELGLSYESVYLDFNKGEQKAPEHTKYNPNGRIPTIIDHKNNDFALWESDAIILYLVQKYDTEKKISATTEEERFKELQWLFFQASGQGPYYGQAAWFSLFHPEKLPSAQERYKTEIQRVLGVLDGILTGQEWLLGSRCTVSDLSFIPWNNFAIGRLLEGVNVAEKFPNAYKWHMKMAERPSVKTIQATQAAVLSAQ*

>jgi|Phchr2|2685965|CE1685965_102905=GSTFuA1

MSQPIVFYDIPSNDTLKQSPWSPNTWKIRYALNIKGIKYKTEWVEYPDIEDVVKKLGGKPTGKKPDGRDHYTVPVIYDPNTKTVVEDGIKIAKYLDDAYPDTPRLFPAGTDAFQAAFDDFVWSVTLAFPLLSLLLLDVSNSLPPRSSAYFRATREQQFGKRLEEQGGEERWQQLEAGLGKFKGYLERNGAGNDLLLMGTQGGITYSDVQIASLFVWAKVVWGEGSEKWKRLMGFHGGKWAQFCAQFAEYERADD*

>jgi|Phchr2|2971755|fgenesh1_pg.15_#_34=Phi

MVLKLYGNPMSTCTKRVATVLHEKGVPFELVSVDFAKGEHKSAAFVAHQPFGQVPYIDDDGFVLFESRAIARYIALKYRAQGTPLVPDPADLRATALFEQAVSIENSNFDVYASGLAAEKVFKPMRGGTTNQAHVEFLAATLDAKLDGYERILSKQKYLAGDEITLADLFHLPYGAMLKQLDYNYLEDAAKRPNVARWWKDITSRPSWQAVKDKVQ*

>jgi|Phchr2|2948390|estExt_Genewise1Plus.C_3_t20316

MQSDDVIWSVINTQFCSYKVKTTTQNFCRNEYNVTGFCTRQSCPLANSRYATVREHEGVLYLYVKTIERAHTPKNMWEKIRLSNNYSTALEQIDKELIHWPNFLIHKCKQRVTKITQYLIKMRRLRLRQEPKLVGVKKKLERREATRERKALSAAKLERSIAAELLERLKSKAYGDAPLNVNEDVWRQVLDREKAGKEGVELEDEESEEEDEEELEEEDEGWGDREFVSDISGDEDDLSDLEDAFEGSDEEEGSDEDEDEPSEEEEGGPKSKVALGKRKAPAGPTKPPKKGPEKKARRGPRVEVEYEHEMETTPLTKEALANW*

>jgi|Phchr2|2977134|fgenesh1_kg.3_#_511_#_Locus4386v2rpkm0.58_PRE= Ure2p8

MSHDKQFSLFLHKASAHGWKVAFVLEELSLSYEIVLVDVAKNEQKSPEFMKLNPNGRTPALIDHGNSDFVIWESNAMVQYVADKYDTERKISMAPGTDDFYIQLQWQYFQGTGQGPYFGQLVWFTLYHEEKIPSAVTRYKEEALRVFSVLERVLSNQEWLVGGKMTIADISFVSWNDMIVHFLDNFDFEKEFPATAAWHYKMLKRPTIKRPWDERRKLMSRQ*

>jgi|Phchr2|2966581|fgenesh1_pg.3_#_219=Ure2p4

MSHGKQFTLYTHNSGPNGWKVAIVLEELGLSYEPVFLDLMKGEHKAPEYLKINPNGRVPALIDHKNNNYTVWESNAVTQYLVDKYDNDRKISVAPGTNEYYTQLQWLYFQASGQGPYYGQAAWFSVYHPEKIPSAIERYRNEIKRVLGVLESTLSKQEWLVGNKATVADFSFLTWNDIAANLLLENFRFEEEFPATAKWNKKLLERPAIAKVWEEKAKAAAH*

>jgi|Phchr2|3031848|gm1.10065_g= Ure2p2

MSHGKQFTLYTHKKGPNGWKVAIVLEELGLTYESVYLDFQKKEHMASEYTKYNPNGRIPTIVDHENGDYLLESDAILYYLAERYDPEHTISVGDAGEKFRVLQWLFFQSSGQGPARERYQNEIKRVLRVLEDVLAKQEWLVGGKATIADLSFIPWNYFAVITIVDSLDFDAEFPATAAWHKKLVGRPAVKKVCYQRRASCMSSEY*

>jgi|Phchr2|2980056|fgenesh1_kg.5_#_788_#_Locus21215v4rpkm0.34=GSTO6

MPEQITLYTAKICPYAHRAEIALALANVPYKRYEIDLKNKPEWYIPKVNPAGKVPAIAYGGPDVPPDEPSPESVKLNESLVLVEFIADLFPEAGILPADPVLRAKARLFIDAVSTKFAPANFSVLHNGGDPAPLVDALAHLQALLPPHGFAVGEFSAADIAIAPFIARTELNLENDLGGFPAGKGEGQRILELIRSPKFARWQEYSKAVLAHPAVASTFDRDHVLESSKRRFAELRATKYSS*

>jgi|Phchr2|3030485|gm1.8702_g=GSTO5

MPMYDPTLTPHATGAALKTVEAHQEPQDVVYHASWYCPFTHRGWITLEEKQIPYQYHEINIYKRDEHYQHFLDINPKGLVPALEYKGKALSESLIIAEFLEDAFPTHTPRLLPTDAFERAQARLALDVVSKSVVPAYFRLITAQGADKQCDAREEFYGVLRAFVDQVRGPYYLGEEFSLVDVAIAPFVVRDAVLKQKRGYDRADVGPKWQEYAEKLETRESVARTTSEMKDYIQFYGPYLRDEADSQIAKAIRKGNVI*

>jgi|Phchr2|3030166|gm1.8383_g=GSTO8

MVQNGQITFYTYLYSPYCDRVLLALKEANADYTACTVDLLNRPKWYTEHINPVGKVPAITYGGPKVKPEEPSPESFKLRESLVILEFLADLFPEAGLLPTDPVLRAKTRLFALAVDTHVFEGFKAFFIMREPVDKLLDGLAQLQQLLPAQGFAVGDKWTMADMAAAPFLLRIFMLLEHDLGVYPAGEGPKTLAILRGERYARLNQYLADLREQPSLKSMWDEAAQIAFWRTTPFLQRS*

>jgi|Phchr2|2978588|fgenesh1_kg.4_#_628_#_Locus39689v3rpkm0.00=Ure2p5

MSHGKQFTLFNHQVGPNGWKVDMLLRELGLSFETVYVNLGQREHKSPSFTKYNPNGRIPALIDHYYNDFVVWESDAILLYIVEKYDPEHKFSVSTFDDKIIMTQWLFFQASGQGPYFGQAGWFLAVAPPEERNPTIAERYQKEILRVFGVLESVLSQRQWLVADKLTIADISFVIWNATAVNLLVKGYKGFDFEKDFPSVHRWHTALITRPAIAESLKTKAEAIAQMNR*

>jgi|Phchr2|3028214|gm1.6431_g

MEPIVFYDIPANNDKCMAWSPNTWKIRYVLNYKCLKYRTECVEYPDIEDLCRAIGAPPTDTKPDGRAHYTLPVIQDPNTKAVIADSDAIAKYFEETYPHAPPLFPNGTRAFQYAFCLPQEYYKRTREQAFGKTLEEIPSQNDWTALERGLETVRDCLDANGEGRDTLLMGEKITWADFQLASLLVWLRVATGQDSEHWAKLLELHGGKWGRFMQQFEKYESVDI*

>jgi|Phchr2|2903766|e_gw1.5.423.1

MPKSITLYTAKICPYAQRVAEIALALANVPYTRYEIDLKNKPEWYLPKVNPVGKVPAIAYGGPDVPPDQPSPESVKLNESLVLVEFIADLYPDSGILPKDPVLRAKARLFIDAVSNKFNPAAGPVVQNGGDTGPLVAAVEHIQSLLPPQGFAIGEFSAADIAIAPFIARAELRLENDIGAYPEGEGQKILNAIRAPHLARWQEYSKAVLAHPAVASTFDREYVLEAFKKRFADLRAKTA*

>jgi|Phchr2|2929653|estExt_Genewise1.C_5_t30038

MMSNPAAQDSAPLTLHYLNDSRSQRILWLLEELEMPYKLVKYQRREDKSTPDELKAISPLGTAPVITDGD

VTLAESGAIIEYILDKYGNGRFLPPESGKIHDLFFRHYAEGTVMPLLVQKLIYGLVPQRSPFIIRPLVRR

IFSTLDSLVVDPRLQIQATFIEEHLSKCGDWLAGGQGPTSADFMMSFALEAWADRSPEMLGPKIREYVKR

IHARPAYQHALAKGGEYKYAKPAL*

>jgi|Phchr2|2932699|estExt_Genewise1.C_7_t30315

MMSKEVVAEPASKKQKKEEYTLYYWPELPGRGEHVRLAFEYAGIPYKEVNDPDKMLSAITEPKKSGFPPH

FAPPALKLPSGRIISQTPAILNHIAPKLGLAGKRDGEDEEETRSIVNQLVLTALDLNNESHDVHHPIDVG

EYYENQKEAALERAKAYRASRLPKFLAYFQKVLESNPEAKTNGGTYLVGSSTTTADLVLFQVMDGVSYAF

PRRIAALKKTGKYDNVFALKERVENEVAIKEYLASSRRKKYGMGIFRHYEELDGEQ

>jgi|Phchr2|2903888|e_gw1.5.402.1

MSNKSDTGKSYHLKCTGDALRTADAHSGPSELTLFGGCFCPFVQRVWVALEYFGIDYQVYEVDPYKKPPE

LLEVSPKGLVPALKFNNLEEPRSLNESTVILEYLEDVAVLQHRGSLLPPIADPYARALVRLQVDHVNRNL

VPAFYRYLQAQDSEKQIEGGKEFLSAIEGLVKLFERTTAETGTDMGLWTEDGKLSLADVMAGPWLFRATN

VLVHYRGFTMPEGTKFRAYIDRLINHNAFKSTCSTEELYLESYERYAYNRPNTSQVANAINSGRGLP*

>jgi|Phchr2|3019931|estExt_fgenesh1_pg.C_120283

MSSSSLKVSTNAFPYAAAAVACLTGDAALDFTNDRHQPVLERPDGTRITGEFDIISALAQDGGVAAGSTQ

SPYFIGLAQSLRGPNSYSSISRTFDILDNHLGYRTWLDGHEMTTADWILWGALKGSPMSVGLLKLGRHRH

LLRWFTHIESLDQTQAALRALADARAQEAELRSRTPTPYTPRSPAVLDAETAAAERVLAERAVADKVGAR

PSVHPMGEAKPVASNPYFAGSAS*

>jgi|Phchr2|2986296|fgenesh1_kg.12_#_38_#_Locus3872v1rpkm66.34

MTFLNRLLVASPKFCRNTHSLLQPYSHIYRGMSVAASIAKLHSPLKELVSNVADAHHGKSEKDKAEVTEW

IEKVAAGDAVKPDGLKDLDGQLTPKTYIVSNYFTAADVALYGALHPFLAQLQPAQYYAHPAVTRYFDHIQ

NRPSVREAAAPLAPAFALVPFDLEHAPSIERKADPPKEKKKAPKAAEQPASSAAPAPADKKSKKKEAAPV

ADEEANKDASAPVPKEKKEKKEKKPAQEGAKKAANAPKAAEDAGEPVPSMIDLRVGHIVDVKKHPDADGL

YIEQIDFGEETGPRTVVSGLVHYIPIEEMRDKYLVGVCNLKPANMRGVKSFAMVLAATHKDGKEAGIELI

QPPPGSQPGDRVYFEGPEFENAQPLSQLNPKKKIFETVQPGFTTLETREAAWVNPATKSVHRIRTAKGVC

VAPTLVGASLS*

>jgi|Phchr2|2973331|fgenesh1_kg.1_#_88_#_Locus18034v1rpkm1.14

MAIEIPTAVKSIFSHFPLHTYPATYSPYQAQPIRTPTLWVRPPRSAKAHNEPGSPDVLSGDVECLKWQAY

LALRFSQAPQPTRVAVRWDVSVSGALDGRLPNLHVPLDTVPASLIGSSEGREKKVKDDGEGEVLPAHHIP

EWVNGRLGEENGELEGYRDPDARDESRAWVALLEGNVHAALLAFSPAETSLLEKISPDPSSKSLLTGGSL

SVSLIPPPAPLTGLLSLLPAYGERIDLHAIEEKYRGAIKALSDRLGEDTWILGSANPTPLDALLFAYLHA

LLHAKDKLLRFEVERRENLVAYEGRIRAIVTNAFVPYRK*

>jgi|Phchr2|2975028|fgenesh1_kg.2_#_79_#_Locus4622v1rpkm52.62

MQLPRPEITELLGLTYRRIPILAVGNDIYCDTSLIASALERRFPASAGFNTLFPPRAGGGRTDGALARQF

SRYWADGVLFRLAADSLPYAKFDQKFLADREAFFGGKIDAKAIAEGQGMRSSAIATHLAMLEEQLADGRQ

WLLDTESIGLVDISGQLFCSWMKQFKHLKEIFSPESFPKTLAWMDRVAKHLNEAEKKGVATFDKLTADEA

AKVIASSSHEDVKAIGFEAAEGARLGVKLGEVVSIMPLDSGRVPTSGPLLALNREESVIEVKGKAGTFHV

HFPRLEFSVRPGQPTAKL*

>jgi|Phchr2|2973443|fgenesh1_kg.1_#_200_#_Locus6298v1rpkm30.40

MAAHTALPVLHIWPKTEQNLSFDPTCVAALLYLQLAIPNQFELDYTTNPDPSPSGQLPYLSHGLHQVAGF

EPIVRYVERLAVEGKRDLDEGLSPSEKAQNAARVAHIESTLGDFVAHEYYSLSANWTRQTRHTLATVLPF

PQKYYVPERIRQSWKPRLEAAELWDVVGIEEEEEKERQRFSFRLQKKRTKKAQEKLRFKETFERKKVIDK

AKAFLDIYSRMLGDRSYFYNVNFPTSLDVVFAAHIHLLDARQPDPLFSKLIEESYPSLFAHCNRVFAIAF

TDNFPCTVTHTGFSLRSIFPHLPAPRTHQKLHSPAWEAQERQFRLMRWGFFGGVGLFVTTYIYLSGFIGM

WLDGYAQIRALQAAEREAGEQADAVDDEEEEEDEEEEEEGGEEEALLEEEMEAEVASEV*

>jgi|Phchr2|3032803|gm1.11020_gMAPEG

MSIVLPEGYAYTAAAVVSTFWLTTFQSFRVGRARKAAKIEYPQPYAEKAEAAASKEAQIFNCAQRAHQNT

LEWLPQVISATFIVGLKFPRFAAAACGTWTLARFLYTIGYASGDPKKRNWLGAGYVNGLTVLSFVLGAST

YTAFKLVTA*

>jgi|Stano2|7496|jgi|Stano2|7496|SNOG_02412.3:1-414

MSFGKLYSYSGNPRTTSLLAVAKENGLDIEFVDTEPAKGVSTEYLKLNKLGKVPTFEGADGFVLSECIAIAVYLASQNEKTSLLGKTKQDYATILRWMSFANTEILTPLGGWFRPILGRDPYNKKNVEDSQKAALKAVHVLEEHLLTHTYLVNERLTLADLFSASIIARGFQYFFDKQWRDSNPNVTRWYETVCNQASYSAVAGKLEFITEALKNVAPKKEGGEKKKDAPKAAKPKKEEPEEEEEEAPAAPKPKHPLEALPRATFVLDDWKRKYSNEETREVALPWFWENVNFEEYSLYKVDYKYNDELTLTFMTSNLIGGFFARLEGSRKYLFGCTSVYGESNNSVIKGAFLVRGQEALPAFDVAPDYESYEFTKLDPTKAEDKEFVNDQWSWDKPIVVDGKEYPWADGKVFK*

>jgi|Stano2|8800|jgi|Stano2|8800|SNOG_02588.3:4-330

MTTEIHKYGTDDGWHGVIKEGGEFPPEKDRYHLYIGLFCPFAHRPNLVRHLKHLTSTLPISVVRPYPKGEPGWRFDETYPNATPDHLFNSRFMHQLYFRDDPAYTGRYSVPLLWDKKTNRIVNNESAEMLKWLPHAFDSTIEDQKIREIDFYPQDLRSKIDEISLWLTSLICSGVYKAGFVSTQEEYEKNVVPLFAALNKLEDLAHKNGGPYILGEKMTELDLLAYPTIVRFDTVYVQHFKTNLGMVRHDYPVLNNWLKNLYWNVEGFKESTDFKHIKENYTKSHSDINPHAITPLGPYPDVEEDYESDWSKLKPGKVAHPRVLEAMSKL*

>jgi|Stano2|5281|jgi|Stano2|5281|SNOG_11730.3:3-254

MSSQNSDITLYTTQTPNGIKISITLEELGLPYKVHKIDISKDTQKEPWFRAINPNGRIPALTDTFTDGKPINLFESGGIMQYLVERYDTEYKISFPKGTREYYEMNNWLFFMNAGVGPMQGQANHFTRYAPEHIEYGVNRYQNETRRLYGVLDKHLAADNRPYIMGEKCTIADLAHYGWIASAGWAGIDIDEFPTLKAWEERMTARPGVEKGRHVPDPHTIKELLKDKTKMEEYAAKSRAWVQKGMKEDAAKSEKL*

>jgi|Stano2|3095|jgi|Stano2|3095|SNOG_09550.3:3-275

MSSDERPTGLKANKGIELLTFGTPNGWKASILLEELKETYGKDFTWQSINISQNIQKEEWFTKLWPQWPHPSDCRPRPGWFRRAGRSCQAYEHPAILTYLTRNYDPEYKFTFKDPLDYSRAEQWMAWQHGGLGPMQGQANHFNRFAKERIAYGMQRYTGETERLVGILDTQLKSNDYLVGNKYSIADIASFGWIHMLRFSGVDLDRFPNLKAWWERINARPAVQKGLNIPSKPGIGNDGYLQKLKDDPEFAQKEKELAEQIQKAKEQYGYKYSSP*

>jgi|Stano2|11672|jgi|Stano2|11672|SNOG_05786.3:1-251

MSATKKARTHPQYELLYWPGMPGRGEFIRLAFEAAGRDIISALVFSNSKGDSDSNPPVFAPPALRIPGAGKNGKPLLLYQTPAILNYLGASLGLAGDDEAEKAWVLSHTLTALDMNNEAHDTHHPVATGKYYEDQKDESLKKAEDFRQNRIPKFLGFFERVLQGNEKEGQGKHLVGAKLSYADTTLWHVLSGLQFAFPKELEVRKKEFPALFETFYPSVQEHKGLKEYLASDRRMPFSMGIFRHYPELDRQ*

>jgi|Stano2|7078|jgi|Stano2|7078|SNOG_01866.3:1-257

MANQQGAKITVYWLEKSRGQRAVWLLEELGLEYELKVFKRDENFRAGDDLGAVHPLKRSPVVGITPAGSEKEIILAESATIVDYVSEHFGKQLIPQRYPDGKEGVLGAETEEFMRHRFLMDYAEGSFFTILMIALVIGNVKNAPVPFFIKPLTRGIANKVETTFINPELKKHCDFLEDFMSKSSGEFFVGDKLTGADIMMHFGLEGACQRVPLTESTYPKLYQYMRRMQKRDAYERAAKRVSEASGETYVPYSDLKIMKREEYESSINKDTNINRIRGEGFKTWLNTLGAKRSEWTAGRQQHVVPLSEIARRQAKRFYFASSTISVRPASPASSSPLSTPSAVAHLSAANMATSPPPTSTGSSKVDAAKANIAANIDPTAPTGLALYSRFAFAGAVCCSVTHGALTPVDVVKTRIQLDPATYNNGLIGGFRKVIANEGAGAVWTGFGPTAAGYFLQGAFKFGGYELFKQQAINMVGYETASNNRTAVYLASAACAEFFADIALCPLEATRIRLVSEPTFANGLIGGFGKILKNEGVGAFYSGFGPILFKQVPYTMAKFVVFEKVNEAIYQVVDKTKTSNGMQTVYNLSSGLVAGFAAAIISQPADTMLSKINKSKGLPGEGTTSRLIKIAKELGLRGSYSGIGARLFMVGTLTAGQFAIYGDIKKALGATGGVEIAKTN*

>jgi|Stano2|7066|jgi|Stano2|7066|SNOG_14093.3:13-199

MDKKPSGSILDWADKSGEFKRQQSVFRSWIENKPNAEFPPEKGRYHLYVSYACPWAHRALIVRELKGLNDIITYNSVHWHMGEKGWRFATADEKVPGETTPDPLHKDFTHLRDIYFEQNPEYEGRFTVPTLYDKKTNRIVSNESSEIIRMFYTAFDDLIEEQYKNVDLFPKALQKDIEAMNEWVYHDVNNGVYKSGFAT*

>jgi|Stano2|616|jgi|Stano2|616|SNOG_00776.3:1-281

MGLSSLDVDTTTGEAFAPGRGTASSDYEAAKRAAGIPTGGQPSALRMGRGKGIGGFLGTPAYAARFRLDSLSNELLDPVADLLGKTDFLLTEDRISSLDCLAFGYLSLLFYPAVPQAWVKETIQTKFPRIASYIRRLRKDIFCDEDIKPADVWTISSGPPQASSGMLLPWQPRSQGFMSSALDGAREIAGNLPLVSWFTQGTSVVHMDYPLSSKNMRSSMPSPVFINTLLGATAAATIGFASLAIHHRRSPREGALIFWALRPSTGFGEAGDILSIFANQMPTHLSKQNICFFADIVGQSGEDNLYDQGRNPGTTNTNNFVLIYPNLSTFNRTPANKMHRTYSMRQSRAPTASQIQNPPPPSSSTKSGRFFGKANIGHTFRHKSAGAFGPDLAKKLSQLVKMEKNVMRSMELVSRERMEVAQQLSIWGEACDDDVSDVTDKLGVLIYEIGELEDQYVDRYDQYRVTIKSIRNIEASVQPSRDRKQKITDQIAQLKYKEPNSPKIVVLEQELVRAEAESLVAEAQLSNITREKLKAAFTYQFDAMREHCEKLAIIAGYGKHLLELIDDNPVTPGETRQAYDGYEASKAIIQDCEDALTNWVSQNASVSSKLSQRSRTLSQRRRNATRRGEGVDLSGQDQTMDRESGLWIPASEHQGNGYDDRDELDDDEAHSTIASEQRGREEERVIAA*

>jgi|Stano2|10223|jgi|Stano2|10223|SNOG_03759.3:14-233

MNNNPINRNPLTKMAQPKITLYVDIVSPFAYIAFYILKNSPVFKQCEITYVPIFLGGLMKACGNTPPLHIKNKDKWIDAERLRLCKYFNVPMSQNTPPGFPINTIAIQRALASLEISHPQSMPQAIGLFWENFWVQYNDPMKPENLSAIVRTIVGSEEGAKKVLESTKGEEVKKRLSENTDKALKGGAFGLPWFEATNAKGETEGFWGVDHMGQMCDHLGLERPSGKGWKALL*

>jgi|Stano2|806|jgi|Stano2|806|SNOG_01009.3:1-224

MSGRITSYLDCVSPYSYFALLRLEENRALLKEHNVEIDIVPVFLGGINVGSGNKPPWTLPAKGAYGRYDSERSKRYFGVPNIKTPEFFPILSLLPQRALVYVKEAHAQKFIQAFKDIFQGIWEQGLDVSKPELLAQVLSKQFSETEVRDILEKANSAPYKQRLNDNTKEALDLGAFGCPWHVLRNSKGVEEPFFGSDRFHYMWEFLGLPWKDIELQAPGSAKTKARI*

>jgi|Stano2|1098|jgi|Stano2|1098|SNOG_06949.3:414-606

MLDAMQVDERLSPVPALQQLQALHPNALTSSSAFSRWVAICSPVQYAAFPGYQHASSPRYGPVQQLQGSPQLAHHQPLPRPPPVRAAPQQPIVQPFQLPVQQHAHADTAVQAQGADADDAPASNHGQFHGLRLIPDPPDLEAWRQKLFDVDEMITLNEDEFKIYFPHIDNVYSHRSTQRHKRKRFVSHYWDCRLKGRPPGTKKSTDPDKKKRKRVARERDLCDVKIKITEYFDHQEYVEQVGHDPPDTGGDATSQSQNFFGQTQMGQQSRDVDGWDMPTNMAQSIPQFATGPGSPSTSRSSSKKYYTFQRVNGNGGNGKGDGVAGPHKHTLEESDRVKKNSVFRWLAKREKGDRRKSQVSVSSFSGVIVCAVHLLSKRSLLQAKATSYIGSLQTLLELAASGPAENLETIRSNMTDTNQGGDPTKKTYHKKATGNAWTTVKNHSQENDLKLYGSAFWSVSLSLSLAPNFPLLTPHSPFVQRVWISLEHKNIPYQYIEVDPYRKPQSLLAVNPRGLVPALRHGPTWSTHESTVIMEYLEDLHAGPPLLPADAQSRATQRLWADHVNRNVIPCFYKLLQAQAEREQVEHAKELREQINCAVGVAAEEGAGAV*

>jgi|Stano2|173|jgi|Stano2|173|SNOG_00220.3:861-1101

MKASLFGIGALAGLASANINFEWVQPICEFSSLLSNQCLTGQHCSEVNACVADLKSDFSKISARSRSSIRFPEKRQSGAKYSTDGKCGPANGNLLCDPNSTAYTGSCCSQYGWCGNTPAHCGDGCLSGCNNGAASTPTKQTTPTQPATGNSGAAAPRADGRCGKDFAGASCDAKGAYGGCCSSYGYCGSTDGHCLVANGCQNGCKDSAPASSAPVSSPSNTRPPSVTSTGEPVLGKPSTPASTKPTGVPTVDGSCGAKFGGTVCGNWAQGSCCSMYGYCGNTTSHCGEGCQNGPCAKAPSQPAPAASPAPAAQKPGTLVKKGRSGVPAMHAGLMPNGKVVFLDKVENYTELKLGNGQYAYSSEYDPNTQKLTPLAYKTNAFCSGGIFLADGRFVSLGGNAPLDFIDPTVGDGFKGIRFLSRTADGKLDGQAWNEPGTHLDTPRWYASVQIMPDNKIFVASGSLNGLDPSKPENNNPTYEILNADGTPQGKSINMEILSKNQPYYMYPFMHLMKDGNLFVQVAKSAEIFNVGTGQAVRQFPDLPGSYRTYPNTGGSVMMPLSSANDWNPDIIICGGGPYQDITAPGDPSCGRIRPLDANPEWEMDSMPEGRGMVEGTLLPDGTSVWVNGAQEGAQGFGVAQDPALEVLLYDPNQPKGKRWTTGPKSDIARLYHSVALLLLDGTLLISGSNPVEQPILTPNAKNPFVTEFRNEIYTPPYLQGNPTRPSDVVLSSKNLKADGSKFTIKFTAPANNKAVKVSLYYGGFVTHSVHMGHRMAFLDNTGFKAGSTTQTITVTMPPNRNVAPAGPYVVYVLVDGVPAMGQFVMVPKHMTGSAIAYICKIEDIEDTNSKGFNTIRFTMAPNIELLTAATPNGQKISIFLEELGLAYTTTAIDLGKDEQKSSSFLKTNPNGRIPAIVDHSRSSFPVFESGAIFLYLAEHYDKDFRFSFQDADEKSEMIQWLFFQNAGLGPMQGQANHFFRYAPEKIEYGIKRYQNETKRLYSVLEARLKGRKWLVGEKYSVADMSTFTWVRWAPWAGVELDEFPGLKEWAERIEAREAVKKGLKVPGGEDQIDRLRRDPNVGDPFQEWVMKGQNEIAEKHGK*

>jgi|Stano2|1974|jgi|Stano2|1974|SNOG_08085.3:1-148

MTTSKPLIFYDISSPIQPRSYAPNPSKARLALSFKQVPFKSTFIDIPDIPEVRKGLNCPATRKFDDGSDYYTLPVLQDQSSNKVIGDSYDIANYLEDTFPNSGGCLFPKDSTGTGLDYESPNKDTQFYAPITTNKGSKNEAYARFNLHVDATFSANVILVTHRLPFNPESAEATKALFVKRAHMNSWDDMSVHGEAREKTRAGFKEALKSLAELFTVHQGGPYLEGEKANYADLIVGGWLNMLSETMPEDEWKDFRTWHGGVFAKLHDALQEKYYVCE*

>jgi|Stano2|926|jgi|Stano2|926|SNOG_06740.3:1-216

MILNFKQIPYTTSWIEYPDLAPQLSSLGIPPNDRSAPDYKTDYAIPTITMPDGTHMMDSWPIAHKLEEIHPSPSLRLDDPIVLKVRDQIANIMKPLTGFVIPKVPKRVLNERSAVYFNETRKERFGMSLVEVEETMATGEKWEEARVPVLEAVGWLEESGGPYFLGETVSYADFIFVGMMHMIKRLDEDVFQRFLAYDPALPKLYDACKPWLEKDD*

>jgi|Stano2|12114|jgi|Stano2|12114|SNOG_06360.3:1-236

MAQVNGNAPKITLYTNHKCPYAHRAHIVLKELGLEYKEVIIDLDRPREDWYLKINPRGLVPAIDFNGEILTESGIVATFLADAYPSHLFPAAGNPKDALTRARINFFADTWSSKAGSYWFQILKADGAEKDSLVQAFVDVVGKEVEPLLADAAPFFGGSKQVTLAEAMTAPFILRAYALTKHGLLPQAVVDGFDKLPNFSKWAREVSKLESVTFIWDEQATMEATRKRIEKMKAQAK*

>jgi|Stano2|6876|jgi|Stano2|6876|SNOG_13853.3:1-152

MAIISIPGDYGYVLLAAVSTFVVGAWQGGRVGAFRKAAKVPYPFEYASYEQIQTASPASSKAMLAFNSAQRAHQNFNENHPTALGAMAIAGLKYPTATAVLGAVWSVNRVIYAVGYTNGSEGGKGRYYGILWMLAHYVMIGMAGKAAWDLAMA*

>jgi|Stano2|4339|jgi|Stano2|4339|SNOG_11211.3:4-127

MSHVFRRGLTRGCVGEDDPRYKGNDARVVIDLKHLDNRLLTTNAWLAGEDFTAADVMIGFCLTTMRKFEPIDLTEYKGILGWLKRVGERDAYRRAMKKSDPDLDIDAGLSAKGPEVIQMFVNAMALKK*

>jgi|Stano2|9369|jgi|Stano2|9369|SNOG_15716.3:25-232

MVQGHPDADLHPHATGPAAHIVKPLKLYSGWFCPFVQRVWIALEEKKIDYQYIEVNPYNKPKSLLDLNPRGLVPTLQYQNKPLFESTVLCEFLEDAYPDHTPHLLPKDPYDRARTRIWTDYVGSRIIPAYHRFLQHQGEEGLKDKQQEFLNHLKEFTKEMDPEGPFFMGQEFSLIDIVIAPWANRLWVFDHFKGGSGLPEEGKGGEDEEVWKRWRKWLSAVENRKSVKETLSEQRGGEGD*

>jgi|Stano2|8983|jgi|Stano2|8983|SNOG_02826.3:4-190

MPPTLHLYMTPGAVSLASHIALRETGLEFTTTDLEAIRGYPSEHLHINPKGRVPVLELDGERITESPAILSVISALAPEKKLLGATILEQARAQEWMAWLCGTVHGQAFGCIFRPMRFVGGEEGMYEIVRAEGRKCAKECFDFIEGRLKGRIWGNILKMGMRDNYPNYTRLVEEVVKREAVKRTVEVEGLSLFNE*

>jgi|Stano2|118|jgi|Stano2|118|SNOG_00150.3:1-197

MPPITLYFLQASRCIRTAWLLEELGLDYELVFSDRVNQKAPEDFKLASGNPLGKFPTIKDGKLTIGESDPAQRVKVRQWIHASEATFALHALAILYARWNVKDVPEGALEAAEERMAVNVQNDMSWLETELSLSPGQFLCGDHVTAADIMMQFSAQFILARDLGTLGKEWPNINKWLEACKKTDSYQRAVKKTGYEL*

>jgi|Stano2|1611|jgi|Stano2|1611|SNOG_07604.3:6-154

MSIKPIEIHGKHGPNPPKVRMIAEELGIPYNLHDVQFSDVKSPEFTKLNPNGRMPAIVDPNTDLTLWESGAIIEYLVEKYDKDNKVSFPAGSKEAYLAKQWLYFQVTGQGPYYGQAVWFTRFHPEQLDSAKERYYKEIQRVTSVLENHLKSQPKGEDGPWLVGGKYSFADMSFVPWQNYASQLTDVKEYTVVADWLERMKKRAAIKKTLDDQ*

>jgi|Stano2|11681|jgi|Stano2|11681|SNOG_05798.3:13-202

MQPIKVHVIPAGPNPWKPILVLEELGVPYEINSFGFEVVKQKPFTDINPNGRAPAIKDPNTGITLWETGAIIQYIIEQYDTKHVLSYDTLKEKHLCNQWLAFQISGQGPYFGQAGWFNVLHPEKLPSAITRYNDELKRILGVLDGALTGKQWLVGDKMTFADLSFVTWNDRIDSLIVCAPDKKFEGFPNVQAWHERMTGRAAWKNIMKKRDQLMDDQGLQPNGMPKGINSFEEYEAMIKANSKA*

>jgi|Stano2|7577|jgi|Stano2|7577|SNOG_02514.3:69-119

MYAAEKSGNDSMYPKDLKHRADINRWLLWEASSWFPTTYVYLVENVVKPLMKGQPDQKVIDAEADKFHRSAGILEARLSKSKWIAGDHVTIADIALAAPMQTYKDMKLPLENYPNLRRWMSEGVEQLDSWKGTAAAVEKALFPDRVRASNSVRTVVNYTKAVDRLTEIYFYESDKAKDVHEPGDAPVEISVSNGWPMAKGFSLDKNGFSVHDFRSKHEVWDDDAAVKSQFYPEVVDFLKKTTGAKRVLVFDHTIRSERNSQKKLTDEKNTSQRTPVMLVHCDYTAESGPVRVTQLLGEEAGNLLSRRFAFLNVWKPLNIVEERPLAMCDVKSCADKDFFKLFLRYRDRVGENYVMSHNPEHKWWYFPMMTPQQAILLKTYDSATDGTARFVGHTAFEDPTSKPDAPMRESIEIRTICFF*

>jgi|Stano2|8032|SNOG_14644.3

MSTKDQLPVVLFGYDSIGREVYCDTSLIIEALEHFFPSSSGYGTVYPEFPGLDSWSYKGLVRGFASFWTD

KPLFRSTTGLIPSSVWETSFGTDRSQLIGHQLDPEKLGAKIPQNLANLDMHLAMLEPTFKTKGKWAIPTP

TPSLADVSLYYQLRWGVDIANGRGIENLSGGGTKDTNREDVTAKVWNEQRYPGLWSWFHAFEKYIDGLPD

LQKTNEDGWLDAVKESKPLSEEDLLVPAAVDGPADVQRDLVPGASISVAPDDTGRDNPTLGTLVKFGVEE

VVIKPHEKAEVDVRVHFPRLGFVVKTVEGAKL*

>jgi|Stano2|119|SNOG_00151.3

MAPTDKPILFHYPPSIYSHRVLWYLWLRGIAYDECVQPPVMPRPDLASIGVGYRKIPILAIGKDVYCDSR

LIISKLEELYSGSTLTPSTPGEAGIRKLFENLSVDGGVFANVVRLMPYWSDSGLLQNKVFLDDRQKLSGG

RRMTKEAMEAGRPDGLQNIRNVFDLFESTFLADGREWILGTNEPTVADIDAVWPFEWMIVDPYMKECLPQ

QNFNDRIYPKVYAWVRRFMDLVAEKKQAYAMPTTLDGEAMASQTLSASSPADDIGFINDDPLDFKQGDEV

QIFPSDYGQMGVSVGKLVGLSTNEVVIENDKGLHLHFPRWNFSIKKVSTSIARAPSTISEAQAIPKMRLI

YHHQSPYTRKAFMLAHELGLAKHITLQKVVVCPVPIAGWSDNNDDVSVFNPMTKIPCLVPDNVPDGIYDS

RIICEYLEHMASVTRTKDAQYWQLHTLHACADGIMDAAILITYEVRIRKERNLYFDEWVEGQKQKIVRGL

DRLQVAAKDGILPDPSAAPATADEVAVAVATAMTGNMGHLGIDWSKGRPQLEAWMKKWESRPSFVATPPL

KEWGTSVDIKTASKM*

>jgi|Stano2|8200|SNOG_14899.3

MSATKPPNDIILYTYAFSPFGKRVAAYLALRGVGYGLCEQPFTMPRPDLALLPVQYRRIPILTIGSDVYL

DTRLILRALEAHPGLTGAPLGASKPADKFVGQLIEKYIVEGPVFGMAAGLVPVKMAHEPTFFKDRQGMLG

RNWSKEELEGGYAECLNYIRNMVSFFEETIMADGRSWVFGEEGPKLADINALFMLDFAAGLQLPEDFISA

KLYPKVFAWFDRYRGGRRYSKVECAPTLQRSTAKLRRLSSHSSGSASAHVSVDAKDPTGLKHGAEVEVYA

ADWGTEYRDRGRLVGLTPDEVTIAVKTKGKAEIRVHAPRTGFKIREI*

>jgi|Stano2|7023|SNOG_14044.3

MSSSTLDESLTTFLKTHAPNNAGAETDAVKASQTLFPEVAYTDAEKAELSQWLITASHIASSSEDAAKSS

ERLSSLNTHLSSRTTLLGAKPSVADIAIYQKLAPVVSKWSAEERTGEQGYHHIVRHVDFVQNSPLFGLKL

DEKVNVELDNVVFKIKPVDAKAEKERKKKEKEAAAANAVASGATPTTLTGEQGATKGGQGGKSAKEKAKG

LAQAAGEAVAGTVTGKPTSGGAPEGAPTQKKEKKEKKPKPQKAAPVEKPLSPALIDLRVGHILKAETHPN

ADSLFVSTIACGDAPGTDNTSEYDGQVVRTVCSGLNGLIPLAEMQNRKIVAVCNLKPVTMRGVKSCAMVL

AASPRVAEGEDSHKGPVELVNPPEGAKAGDRIYFEGWEGEPEPVLNPKKKVWEMIQPGFTTTDALEVAFD

VGVVPQLAGEGAEKKSGVAKLRTKEGLCSIPTLKGAVVR*

>jgi|Neucr2|7749|NCU01320T0

MAITLTLPDEYGYVLLATVSTFFANSFHSINTGRQRKAAGVKYPLAYAPQEVAEKDPKAFAFNCAQRAHANFTENLTPAIGAMLIAGLKYPVLAGALGGLWSLTRVLYTIGYTKKGPQGRTKFGIASSLSLLALKLMAAYTAVQIAFH*

>jgi|Neucr2|7748|NCU01320T2

MAITLTLPDEYGYVLLATVSTFFANSFHSINTGRQRKAAGVKYPLAYAPQEVAEKDPKAFAFNCAQRAHANFTENLTPAIGAMLIAGLKYPVLAGALGGLWSLTRVLYTIGYTKKGPQGRTKFGIASSLSLLALKLMAAYTAVQIAFH*

>jgi|Neucr2|7747|NCU01320T1

MAITLTLPDEYGYVLLATVSTFFANSFHSINTGRQRKAAGVKYPLAYAPQEVAEKDPKAFAFNCAQRAHANFTENLTPAIGAMLIAGLKYPVLAGALGGLWSLTRVLYTIGYTKKGPQGRTKFGIASSLSLLALKLMAAYTAVQIAFH*

>jgi|Neucr2|9898|NCU09570T0

MASKDNEGSGAFIRSESAFRHFISPDPNAQFPADKGRYALYISPTCPWAHRTSIVRILKGLEDIIDLYELHASMGPTGWYFSGKGSSFPADPFYGFKYLPDLYKKADPSFTGPFTVPMLWDKKTHTVVNNESSEIIRMLTTAFDHLLPEHLREVNRPGGGLYPKHLQSSIDEVNSWVYNLINNGVYKTGFATTQQAYDANLYPLFEGLDRVEQLLSSPAPSDGGSPETKSRKYLLGDNLTETDVRLYTTIIRFDVAYRPVFQCSLKSIRHDYPNIYSWLRRLYWDQDSRETNSAFHSTSAPYLNMYGPSYAAARHKKVFGGQGPFIVPGGPTPLIDQL*

>jgi|Neucr2|8100|NCU04109T0

MNPTVLHTQSILKASLLETHHKLQSSTKHLTHSTSDTMASQNSDIHLYTAQTPNGIKVSILLEELGVPYKVTAIDISKDVQKEPWFLEINPNGRIPALTDKLEDGTPIALFESGAIMQYLVERYDKDHKVSYPQGSKEYYQTQSWLFWQMGGLGPMQGQANHFTRYAPEKIEYGINRYQNETRRLYRVMDAQLAKNEYLVGDRPTIADFSCWGWVAAHGWCGIKNFEAQFPHLNAWLNRLLERPGLEKGRHVPSKHTALELNKLSEEELEAKAVSSRAWVQKGMAEDAKK*

>jgi|Neucr2|10649|NCU05780T0

MADSSSLKPIKVYGHTGPNPPKVIMVLAELGIPYDLDNIQISQAKSPEFVKNVNPNGRLPAIQDPNTDLTLWESGAILEYLTEKYDKELKLSFTPGTNDFYLARQWLYFQTTGQGPYYGQVAWFKRYHPEPVPSAVERYVKELNRVSSVMEDHLQTQKEKYGTEEPWFVGNKFSYVDIAFAPWQHIVGVMLTKEEYDEDKYPLIHAWLERLRARKPIKDALDNMIPAGGPPPKQNE*

>jgi|Neucr2|3908|NCU05706T0

MATTTDHPKITLYWLNESRAQRMVWLLEELGVPYDVKIFHRGKDMLAPAELEKVHPLGKSPVVTVQPTEPGAKEIVLAESPFIAQYLCEHFGKDTTLVPNKYKDGQEGKLGGETEEWMRWQHLLYYSEGSLMPPLLVALILSLMSGPKVPFFIRPITATVAGRVNNFFVVPNIEKHCAFIQQLLETSPNGGKYVCGDKLTAADILISFPLLMVPRAEVLAGEAAKGKLKEKFPKVFDYVARLKEEEGYKRAEKKIADLEAEAKQ*

>jgi|Neucr2|9854|NCU10521T0

MAPFATIYTYPNNVRVQRAQAVAKLNGLEIVEDSDFQLANPSDPKWAAILAKFPYGKVPALSTTDGSLNLTEGQAIVRFLADSGPKSEQLLGRTAVDRALIEQWACFAEQELLTNLYPCMLMVHRPDLVPYSPEGYDASAKKFERAVKHVENTLAKGNGKFLVGEEVTVADVMVAGALILASKLLLDAEMKKNAAPSVEGYLKGLLEIPELKESFGELVTVQERLRGKTE*

>jgi|Neucr2|7024|NCU04676T0

MVRIITRPLLSVGHCTQLVQLRAVTPVPFPLPISLASLPLLSFPSHRALFSTTTIAPTLSSAAERVKMSSEGAAVKRQRSNKDVPYNLIYWPGIPGRGEFIRLTLEEAGAEYVDTAQVEGGIDEVMAYVKGEKPDDKTNPPIFAPPILKHGDLVISQTPNILLYLGPRLGLVPGVEDDPDALYKVNELALTALDGLSNEPHDCHHPIASELYYEDQKEEAKRKVEHYVKTRLPKFLGYFERVLNSKASGEGPYLYAGKLSYADLVLFQCLDGLKFMFPKAMAKLNKEGKHSKLFELYDAVGQRPKIKEYLGGPRRQKYSSGLYRYYEEFDIEG*

>jgi|Neucr2|2188|NCU00549T0

MLPICIPLPDRNPKLDTLLSALTYLHVPTLSKLPRTLSSSIRTPFVRKLNTRSPPVPLQVHHCTMFSASSSNPSTQKPASVDTSLQGSPTGAAALFASSHSSPHPLKLYGGWFCPFVQRVWITLAEKNIPHQYIEINPYHKAPEFLALNPRGLVPTLAVPTSTDPKTGKVKEVKPLYESLVLCEYLDEAYADENVYGDRLLPQDDAYERARCRLWIDHISSRVVPAFYRFIQHTPDKPYTIDEIRTEFHGHLKAFAKEMLHASSPSSSPGPFFLGDKFSLVDIMLAPWAKRLFLIDHYKPGGVGIPPSGQRGSEVDEEIWKRWEEWYKAVTERESVKKTWSEDEQYVGAYKRYAEDTTQSEVGRATRSGRGLP*

>jgi|Neucr2|7402|NCU03826T0

MAFGKLYTYEANPRSTAILAVAKANNLDLEVIKVDLEAAIEEYKKVNPLGKVPTFVGADGYTLFECIAIAIYVASQNEKTTLLGKTKQDYASILKWLSFFNTEVLPPLAGWYRPLLGKAPYNKKAVEDAQATALKAISVAEAHLKNNTFLVGERITLADLFATGIIARGFEFFFDKAWREQYPNVTRWYTTVYNQPIYSAVAPPFALLDTPKLTNVAPKKAEAPKPAAPKPAAAPAAAAEEPAEAPKPKHPLEALPRASFPLDEWKRQYSNVDTPEALKWFWENVPFTEYSIWKVNYKYNDELTLTFMSNNLIGGFNNRLEASRKYLFGCASVYGTNNDSVIQGAFVIRGDDWKPVFDVAPDYESYEFTKLDPQNPEDRAFVEAEWSWDKPALVNGKEYPHASGKVFK*

>jgi|Neucr2|5952|NCU06307T0

MAALDTYKYTPAEEKEVQQWIQKADTIKASDDKQSVLDALNADLATRTTVLGTKPSKADIAIYEAVAPLVKAWSPEERTGQQGRPNIVRLVDFVQNSPLFGLNVADADKIAIDADEILYVKPPVDAKAEKERLKKEKAAAAAAAAQGAASAVQGAAATVVDRTKEAVAAVVEKAVEVKDQVVQAATDAAPAAAAPKKEKKEKKVNENRRPKATPPPPAPLSPGLIDLRVGHILKAIKHPEADSLFVSTIAVGDAPGTEDTAEYEGQVCRTVCSGLNGLVPLEEMQGRKVVVVCNLKPVKMRGIKSCAMVLAASPKPQEGVEDDHKGPVELVNPPADAKAGEKVYFEGFNAEPEKVLNPKKKIWETFQPGFTTTGALEVAFDVDVCKDVKDLEGKSGIAKLVTASGGVCTVPTLAGAQVR*

>jgi|Neucr2|3369|NCU01636T0

MVPKPKITLYLDTVSPFAYEAYHILRNDPIFKNVDIKYVPIFLGGLMNKCSNTPPIKIKNKDKWINVERLRWAHAFSVPIVTDMPPNFPPNTLPVQRVLAGIEASSSQSQSAVIAALDALYKAFWALGQPIYEPAQLRSVLASSLGGEEAADKVLGAAQTAEVKQRLVENTDKAFAEGAFGLPWFTCTNTKGETEGFWGVDHLGQVVQFLALDKEASGNGGWKSVL*

>jgi|Neucr2|2408|NCU02888T0

MANQDQQITFFDIPSKDGRTWTLNPWKTRFALNYKSLPYHTQWLEYPDIRPTLSPHLPPAAPEPSTSSYTIPAIRFPDGTYMMDSKPIAVALEERYPAPQYPSLHLDTPALAKLESLMPGMMGKLVGVFVPGAVKNILGPKSQPYFISTREAAFGMSIDELQKTQGGVQAYEKIREELHEATALLKQNAAGPFFEGDKVSYADFVWAGFLLFMKYADAEGFDKLLEATGDKEANERLLEGVKPWARDDI*

>jgi|Neucr2|7778|NCU01347T0

MLSSSSTSGSQQQPPIILYHYPFSPYAKRIVWYLHLRGIPYTQCLQPMILPRPDLSSLLGIKYRRIPLLSIGRDVYLDTRLILSKLEDLDLPSLNPKLSAAPGTDALALQSLLSHYSIDGGLFSRAAQLLFSLDNPLLRDPQFLADRADFVAGGGAGNAPPSQFSSREEMAALGPEALADLRDYCQMLETTVLADGRNWILKNSTDTDNDNNNNRPGLADIEAVFVLHWVMSLPGALPKEQFNDDKFPRVWKWVERFRVAVGETTTPTQQTAAQKKGDGNNKVATIKGDKARDLILGARWNEDSEKKEVEAGEAIVQFHGLKKGTLVEVWPTDSGSAPAHRDVGRLVSLDSREVVIENEKGVRVHAPRHGFRVKPSLQAVASSL*

>jgi|Neucr2|8039|NCU01457T0

MNSDTRRIQSHHPHRAPGHPKNYYHAYPADRQLRDYVTSPTIIVSAILLFLSALYQLLISDRNTRTRLKNIIWARVVDIIPATLLFKVDGFLNPPLFSRPMTPAEIDDSHEAKSQALGRILGLDKPGGVMESVTSAGLKGLSTLSSVGWNFKNSTSDRPAGLGNNDNSCYQNSILQGLASLQGLPEYLARVSQLGSNAKSTMPTTQAMAGLIATLNDKANYGRSVWPPNVLKNMSTWQQQDAQEYFSKLLDQIDREVAKATATYKKSLAYDGDPPPDDGVSSHHSDDSGYHSSSQSSSVPDIQLSRNPLEGLIAQRVVCVKCNHFEGLTMIPFNCLTLNLGSGQLGYDLYERLDYNARVEFIEGVHCPRCSLLKMQQKIKGLIGMVATDEARVSEFRERLAAVEEALEEDMLDDKTLAEKCKVPAKQRVESTKTKQTAISRPPKSLVIHINRSVFDERTGYMYKDSSAVRFPSILDLGPWCLGSAKKRADLEGSNGVLPSSTSNGEQTAEDEEKWNVEPTASMVAGSQRPSALSGPLYELRAVVTHQGRHDSGHYVCYRKHYISPPEKENQPEAPPPQLVADDEVAAPKIQALDQKSISEQDTTDEEDAPTPTSEDDEKTLMREDEEEATSQWWRLSDTDVFKVDEENVLERGDVFMLFYECVEPEMVRIPEREVTRTLSPQAEPGNHVHDRSESVTLAGSEKPEDVEMVKAEASEAGKDMTTPTGTVAGQSPDTMVQVQHADSVAASGGMEQVD*

>jgi|Neucr2|10681|NCU05807T0

MFPINVAAMPALPTRKALIVVDLQNDFVSPDGALPVTKPDGFVDRILELVKIFRDSGAGDVVWVRSEFEQHRALDTEGDTIIASDIPMRPSPTRRRANSKDHDGKLMGADEEAFLTVVGGLDKPACVRKGTPGANFPPNIEASIMRGRDFITTKSHYSAFPPGQSQLVQQLRMRFVTDMYVCGSLTNVGVFATALEAGKHAYDITIVEDCCGYRSYARHVNAVQQLEKLTGCDLLNSSALIDKLQPTQPRNPAKRGHRTKPSSGSRDVKKPGKAEESKQDRDRLSTAPPKASTNHSTGLSPTMARVSIDLSNLSPIEPLAPSRRTTNSGSSTTPQPRQATSNANVPLTQPMVTHGDLSNPLADSLAPLEAASEADTDNIESEVLSIKRRNVGRISQSLSGSPGVLGAKPVDGADSSPSSQTTRVAVKPRLRRDKTSTSSSSSSPSHFQGNDKDRNSAEALEQIKDTPSTPAISNQPDPNTPQSSTDSPESHTKTSSTTTMENYQKLTVSEPLCEGDTTVITNALSPDLAADAFERLLEEVSWAGMSHLGGEVPRRIAVQGAVDDEGNMPVYRHPADESPPLLPFSPTVLAIKNEIEKHLGHPLNHVLIQHYRNSSDYISEHSDKTLDIARGSYIANVSLGAERTMVLRTKRPPKDKDRKDAPAAAAAAAAGTSESPSAAEKAKRQIQRAPLPHNSLLRMGLQTNMRWLHAIRPDKRSDRDKSSSELAYSGARISLTFRQIGTFLNTSQTKIWGQGAVGKTKEEAQPVVNGQTDEAVRMLQAFGHENHVSEFDWEKNYGAGFNVLHMGSPKRFFAGSVQGRACTVENTRVALALADMGIGVARGAIDASGGSGEDEEEQSERRGRKVKVGVKFVDNDPARTEVVGDVGILRYLDAVYGAGRRYDQMTPAQVAKRFARLEEADELWTAWNWLLNRSNRKTEESGEDKVSSTSRAEKVKQAIREKKEALLKQWEEYAQEAHTAAAKAVVTPTASEAETKTQPEEPAAKDQHNKRQLTPFYICGGEAPSPADYALWPVLHELVIYADGDEEVLRIGKGYLAKYYMAFKQRSAVAKVVLSGGNTGPASANGGEQKRDVATDAEKKEKDTAPGAAPASAAPASVTPAAPAVAAEVPLRKTIGTVVDVDAGEEAKVNKTNGSVSVDEKVKEKM*

>jgi|Neucr2|10194|NCU06035T0

MGFTDLLTDAGLAVLNNWLLTRSYVTGYTASQADVAVFKALKEAPSAEKYPNAARWYKHIATYEDEFATLAGDSSAPYTTYGPDVAEVTINPAKAPEAAAEEEEEDVDLFGSDDEEEDAEAARIREERLAAYREKKAAKPKIAAKSIVTMDVKPWDDETDMVALEAAVRGIEKDGLVWGGSKLVPVGFGIKKLQINMVIEDEKISLDELQEEIAGFEDYVQSTDIAAMQKL*

>jgi|Neucr2|6155|NCU04368T0

MSLRFRLNLAAVRSPTLLLPRSIKSSSLVSTVVVRTSTRNISTLLQPFPLPHATTPNNLPTITKRALNTT

TPTMASSDTITNWVDPNDKTGEFKRQVSSFRSFISRSDPSSPFPPEKGRYHLYVSYACPWATRALIVRKL

KGLEDIISFSSVHWHMGPLGWRFPLAEEGKEGGDAAGDQVVPDPVGGKQYMREVYFGVEPEYNARFTVPV

LFDKKEGRIVNNESSEIVRMLGSEFNEIVEDAKARELDLYPEDLRAEIDEVNEWVYHDINNGVYKSGFAT

TQEAYERNVIKLFEGLDKVEKHLKEVQKKGKGEFWFGDRLTEVDVRLFPTIIRFDPVYVQHFKCNLRDIR

SGYPAIHRWMRHLYWNVPAFRETTNFLHIKKHYTCSHPQINPKGITPLGPVPDILPLDEEVEAVKAAKN*

>jgi|Neucr2|1774|NCU00923T0

MTLELHVWGPAFGLPSIDAECLATVTYFAQTLSAADYLLVQSSPSAVPSHHLPALYNPSTATWISGFDPI

VNYLSTLQPPSYHHPDVTTLPSRVYADSQAYKALLTSSAAPLLALSLYVSSANYSETTRPAYSAILPFPL

PWTEPLAVRAAMAARAAHLGMSSLDTDAEMERLEREEREREAAGWVQIPKALRKAVGGQNSGVKGQLSPE

MKRRIKLEGLAAEVFDVLGEVDFLEEEDGEEEEEEEEEAKEGGARIKVTLETKCLAFAYLALMLLPEVPR

PWLKEVLQKKYAGLCKFVLEYRRKTFPDSGKVLPWADRESDPAVSACDSALSIVGRFVRAVIDDIPMLGR

EWSRWWALRQRRVAEENSAETQLVVRRSVGESERSLLLAGAGLTLLAINVAGLGIYWYRYRGLLGAPLQT

WHRPLVGLGSFGAAGAMFAGLA*

>jgi|Neucr2|10681|NCU05807T0

MFPINVAAMPALPTRKALIVVDLQNDFVSPDGALPVTKPDGFVDRILELVKIFRDSGAGDVVWVRSEFEQ

HRALDTEGDTIIASDIPMRPSPTRRRANSKDHDGKLMGADEEAFLTVVGGLDKPACVRKGTPGANFPPNI

EASIMRGRDFITTKSHYSAFPPGQSQLVQQLRMRFVTDMYVCGSLTNVGVFATALEAGKHAYDITIVEDC

CGYRSYARHVNAVQQLEKLTGCDLLNSSALIDKLQPTQPRNPAKRGHRTKPSSGSRDVKKPGKAEESKQD

RDRLSTAPPKASTNHSTGLSPTMARVSIDLSNLSPIEPLAPSRRTTNSGSSTTPQPRQATSNANVPLTQP

MVTHGDLSNPLADSLAPLEAASEADTDNIESEVLSIKRRNVGRISQSLSGSPGVLGAKPVDGADSSPSSQ

TTRVAVKPRLRRDKTSTSSSSSSPSHFQGNDKDRNSAEALEQIKDTPSTPAISNQPDPNTPQSSTDSPES

HTKTSSTTTMENYQKLTVSEPLCEGDTTVITNALSPDLAADAFERLLEEVSWAGMSHLGGEVPRRIAVQG

AVDDEGNMPVYRHPADESPPLLPFSPTVLAIKNEIEKHLGHPLNHVLIQHYRNSSDYISEHSDKTLDIAR

GSYIANVSLGAERTMVLRTKRPPKDKDRKDAPAAAAAAAAGTSESPSAAEKAKRQIQRAPLPHNSLLRMG

LQTNMRWLHAIRPDKRSDRDKSSSELAYSGARISLTFRQIGTFLNTSQTKIWGQGAVGKTKEEAQPVVNG

QTDEAVRMLQAFGHENHVSEFDWEKNYGAGFNVLHMGSPKRFFAGSVQGRACTVENTRVALALADMGIGV

ARGAIDASGGSGEDEEEQSERRGRKVKVGVKFVDNDPARTEVVGDVGILRYLDAVYGAGRRYDQMTPAQV

AKRFARLEEADELWTAWNWLLNRSNRKTEESGEDKVSSTSRAEKVKQAIREKKEALLKQWEEYAQEAHTA

AAKAVVTPTASEAETKTQPEEPAAKDQHNKRQLTPFYICGGEAPSPADYALWPVLHELVIYADGDEEVLR

IGKGYLAKYYMAFKQRSAVAKVVLSGGNTGPASANGGEQKRDVATDAEKKEKDTAPGAAPASAAPASVTP

AAPAVAAEVPLRKTIGTVVDVDAGEEAKVNKTNGSVSVDEKVKEKM*

>jgi|Neucr2|10194|NCU06035T0

MGFTDLLTDAGLAVLNNWLLTRSYVTGYTASQADVAVFKALKEAPSAEKYPNAARWYKHIATYEDEFATL

AGDSSAPYTTYGPDVAEVTINPAKAPEAAAEEEEEDVDLFGSDDEEEDAEAARIREERLAAYREKKAAKP

KIAAKSIVTMDVKPWDDETDMVALEAAVRGIEKDGLVWGGSKLVPVGFGIKKLQINMVIEDEKISLDELQ

EEIAGFEDYVQSTDIAAMQKL*

>jgi|Mycgr3|55916|jgi|Mycgr3|55916|estExt_Genewise1.C_chr_22005:1-416

MGFGKIYSYEGNPRTTAILAVAKANGLDIELVNENPADGLSADYLKINPLAQIPTFEGQDGYILTESVAIAIYLTSQNEKTTLLGKTKQDYASILKWMSFGTSQFLPGLGAWFRPLIGREPYNKKNIDDAIAKTNKNVDLLEKHLTANTYLVAERLTLADLYVAGIAARGFQYVFDKEWRNTHPATARWYDTIANQDIWTAVAGKVAYIDKAIPNTPPKKEEKPKEAKKQAAAKPKAVAAGNDEEEDDVPAQPKAKHPLEALPKATFVLDDWKRKYSNEETREVALPWFWENCKFDEYSIYRVDYKYNDELTMTFMTSNLIGGFFTRLEASRKYLFGAASVYGVTNDSVVRGAFVVRGQEALPAFDVAPDFESYEFTKLDPTKKEDREFVDDMWAWDKPIDVSGKSYEWADGKVFK*

>jgi|Mycgr3|95660|jgi|Mycgr3|95660|fgenesh1_pg.C_chr_9000184:21-341

MSSQTDDNKDGNGKNGILSWADKSGEFKRQTSSFRNFISKKPGAEFPAEKDRYHLYVSYACPWAHRALIVRKLKGLEEFLPFTSVHWHMGEKGWRFATPDEKNLPGENTVPDPLHPEFKHIRELYFESDPNYDQRFTVPALYDKKQKKIVSNESSEIIRMLYTEFDDLLPEKYKTLDLLPADLKDQIEETNDWTYNDINNGVYKSGFATSQEAYEKAVTTLFKSLDRAEEHLANNPGPYYHGERVTEADVRLFTTIVRFDAVYVQHFKCNLRDIRSGYPALHKWVRHCYWNNEAFGKTTDFTHIKGHYTQSHKQINPYGITPLGPEPAILPLEEEVAAAKAKK*

>jgi|Mycgr3|33235|jgi|Mycgr3|33235|e_gw1.1.3508.1:12-274

MSTNGNSNKALDPNQGGDATKKTYHKQPTGEAKKTADAHSKENELKLYGSCFCPFVHRVWISLELKGLDYEYVEVDVYRKPKLLLDINPRGLVPALRHGDWGMYESTVVMEYLEDLNEGKALLPRDPKLRAHSRLWVDHINRHIIPAFYKYLQAQEPADQVKFAGELKDHISKAVDAADEEGPFFLGSEMGFVDVQLAPWVVRLEKVLKPYRGWPSPEPGSRWEKWVNAIEQSEPVKRTTSNDELYLDSYERYAENRPNTSQVRDAINSGGGLP*

>jgi|Mycgr3|103891|jgi|Mycgr3|103891|estExt_fgenesh1_kg.C_chr_30405:5-267

MSDRPNGLIAKQGLELLTFGTPNGHKASIILEEIKEAYGKPDYVYQSINISQNIQKEKWFTDVGPNGRIPVLVDHDAGGLGIQEGQAILSYVTRHFDPENKFSFKSDPELSLCEQWVAWQHGGLGPMQGQANHFYRLAKERIPYPTQRYVGETERLYGILDAQLASKEYLVGGKYSIADIANFSWVNVAYFAGVDLDQFPNLKKWWERINARPAVKKGVAIPNESTITNEAYQRRLKEEDGFKEKEDELKELGKKAKEQYDYKYSSP*

>jgi|Mycgr3|102530|jgi|Mycgr3|102530|estExt_fgenesh1_kg.C_chr_10601:4-250

MSSNIHLYTTQTPNGIKVSITLEELGLKYEHTKIDISKNTQKEPWFLEINPNGRIPAVTDTFTDGKPIRLFESGSIMQYLVDRYDTEYKISYPKGTREYIEMTNWLFFQNASQGPMQGQANHFFRYAPEKIEYGINRYQNETRRLYGVLDKHLSDTGSKFLVGDKLTIADIAHWGWISAGPWAGIDTSEFPTLEAWEKRVYEREAVQKGADVPEKYKLKELLNDPAAVEKKANAARDWIQASMKAEAERK*

>jgi|Mycgr3|70572|jgi|Mycgr3|70572|estExt_Genewise1Plus.C_chr_32118:11-337

MADDTSSWHAGPDDSFHGKITLNGRFAPEANRYHLYVGYFCPFAHRAMIVYKLKQLEKYAGIEMSIMRPYPKGDENGWPGWRFNVKEEEDQYEGATVDKLFGSKYLHEVYFKADKEYKGRYSVPVLWDKKLSTIVNNESHELLRDLQTEFNPLLPRELQDITLYPEDLREEVDTLGQQLQRDLNTRVYKTGNATTQEDYEENLPIVFAMLNKLEKVAAKSGGPYILGKHMTEVDVRTYASLIRFDTVYVQHFKCNLGMIRYSYPILHNWLKSMYWNEHAFRSTTNFRHIKENYTKSHYHINPLAITPVGPWPDIESGVEADWSKLRVGGIDMPAVLEFERTQTDRE*

>jgi|Mycgr3|86002|jgi|Mycgr3|86002|fgenesh1_pm.C_chr_5000087:2-255

MSQPKYELLYHPGIPGRGEFIRLAFEVTGIPYTDIANSQKDGYATVQKTCMNTTSTASEGSNPPMFAPPALRIPGAGKNGEALVIAQTSNILLYLSEKLSLLPEGDENGKYFVNQLVLTALDLNNEIHDTHHPIGPSLYYEDQKDASLLKAKDVRENRIPKFLSYFERVLKGNGDGKYLVGERVTTADVTVWQVLDGLQFAFPKEMAVRRKEFEALFKWYEGFKEEKGLKAYLGSERRLPYSQGVFRYYPELDRQE*

>jgi|Mycgr3|103202|jgi|Mycgr3|103202|estExt_fgenesh1_kg.C_chr_20222:11-253

MADTQAVKPTVTLHWLELSRSQRIVWLLQECKDIDFKTEVYKRQSDKLAPPELKQIHPLGKSPLVTISSPNIAKPIVLAESGNLTEYLADHFATHLIPTKWQAGKENQVGGETEEWMRYSYYLQYAEGSLMSLLMVGLFIDQIRNAPVPFFIKPITKTIAGRVDAAYLTANYATHFAFLESQLASSPQGGKYLCGKDLTAADILMSFPIMAAKAQLLKKAEYPKLYAYAEMLEGHEGFKRSVKYIEEVTGEPFKATFR*

>jgi|Mycgr3|108479|jgi|Mycgr3|108479|estExt_fgenesh1_pg.C_chr_30174:7-237

MATATAPKIVFYTNHGCPYAHRADITLRELDLPYEEVLIDLDSPRPQWYLDINPRGLVPAMKFTLPGSVDQEQIITESGVVAEFLCDAFPSHLLPATTSPADALTRARIAFFHQTWTDKVSPAQMAILRAATEEERDAKSKETVQILKKEIEPLLSDAAPFFGGSAELTFAEVMCAPFVMRLLAMAEDGELVPQALKTEMEKLPNVGRWTKAILGHEKATRVFEKEKFNEAVKSKLDKIRGVKM*

>jgi|Mycgr3|43227|jgi|Mycgr3|43227|e_gw1.6.1492.1:1-258

YTAGTPNGQKISIALEELGLEYKVHHVDLERDEQKEEWFLKLNPNGRIPVLTDHSPPMHPEKEVHLHEGLAILLYLAGTYPLKGKGSLTFPPESKEYWELLVWLSFAQSHLAPMQGQANHFHRYAPEKIQYGIERYQTETKRLYGVLNTRLVEQESVGEGLWLVGGKFTVADICAFSWVNWGEWAGVRLEDGGGEGWEGLRRWVDVIQGREGVKRGVDVPEKFVMKEKMKTKEGEEEFARYHGNWVVKGMEEDREKHK*

>jgi|Mycgr3|45618|jgi|Mycgr3|45618|e_gw1.7.474.1:4-218

MAKKVTLYVDIVSPFAYIAYHITRTSPVFKDIELTYIPIFLAGIMQQAGNTPPFKIKNKDTWINVERQRWTKLFNVPSIGMPSPFPQSTVTAQRALCYIESVHPDKLVASIDALYAAFWAQGKPIGKNETVIEALSGVFGEQEAKKIVEVEIKKDETKKKMNANTQLAWEAGAFGLPWFVATNEKGEKEGFWGVDHLGQMLNFLEVKRVQEGGWKGML*

>jgi|Mycgr3|31926|jgi|Mycgr3|31926|e_gw1.1.3663.1:3-225

MGLIVHHLGHSQSDRVVWLCEELGLTYELKKYDRSPVLSPPELKALHPIGASPVIEDDGGVKLAETEACVEYICNIHAGGKLLVQPGAKNYADYLYWYHVTNGTLQPAVGRVMALSMAGLKEDNPTLARYQAKVHQVLGYMNERLGSVPFFAGEEFSAADIMAIFSLTGMREFCPVDLSEYKNLLSYMQKIVKRPAYKAYLSKGDPGIDIEQFIQGPPPPLFKGFAGHK*

>jgi|Mycgr3|33631|jgi|Mycgr3|33631|e_gw1.1.3635.1:1-222

MPETKITLYTDVISPFGYLAWNILKNSPVFANTKIVLVPVFQGGVMQMNDNRPPMTIKNKGPYQAFERAYFAKRFDVPFAASGKMPQPFPQNTLPTMRSVCALSLVKPEKVDDYMTALCKRFWIDLEPVSKPEVFGKVLAEVLGSEDEAKKVLEKMGEAEAKDLLKSNTDEAFKSGSFGAPWFEAVNAKGEKHGFWGINHLGLMCEFLELDRNKDGAFRSLL*

>jgi|Mycgr3|108974|jgi|Mycgr3|108974|estExt_fgenesh1_pg.C_chr_40106:1-278

MQLFILGPAFGLPSIDAECNAAVALLQLHGKNEYELVPAHEQRERLPYLIDGEQRIHGFSNIARHLNDQHDPLTSRQRADSVAVTSFLESNAHILLDISLYVGFENYRLTTRPAFTKILPWHANYIIPPQRRAAARSRTEHLGISSIDVDNVHEDMSNRPPGYEAVGKEEGFEKEAQARASLLLPKKNTLKSLLQTPERASIFKLQALADNFFGPLADVLGEKEYLLGTEEGTSVDCLLYGFLALMLFPKVPQDWLAQTMRQRYGKLAAFVERVHTTLGLTTDTEEVVKLLQNRSKAESLSQREASKSSLPWTIEPQMNIAETFKSIGYALIEQIPFIGSPNSLQLMSTHKASTLSRHMRAVVLSATASIALSGYIALHTGLLVWPHGEEIHIFGRKRFSDLGHLGAALAAPSIPAPKKRKRPTKKKDDPIKAARKAFEELVGNEVATSELAETADSLSDAAVVDVSSDDKDKDKDEELATDCNLDKLSETKQQFVLAKRHKKAYKAAKEAAVINKKAKKRMHRAVDPKERLHKKTISARIKPFAKSLTALTRNEEFLKEVAFEEKCEVLSDLCRVDYIANIAAGMLSKPA*

>jgi|Mycgr3|105101|jgi|Mycgr3|105101|estExt_fgenesh1_kg.C_chr_70091:7-236

MPYEPYTDETPADVKNAKGLHLVTQNTPNGQAVQIFLEELKDAYGTEFTTTVIDISTNEQKKDWFLRLDPNGRIPILIDNTQSPPFPVHETSAELFYLLKFADKSDKFGFSSPLERNQCLQWTFFWHGSGAPYQGQVTHFTRAAPEKIPYAIDRFRNETLRVFGVLEIHLSGKYTGEEKEYLAGNGKGRYSIADIKTYPWVKNWERSGFSKEEMAFPHLLKWVDRIAERPAVQRGVGEAYVKK*

>jgi|Mycgr3|83481|jgi|Mycgr3|83481|fgenesh1_pm.C_chr_1000191:17-199

MSTEITLFDLPSKGTPTCWSLNPWKARMVLNFKKIPYKTEWVEYPDLASTFKSFAIAPNAPPQAEYTSPVVRLPDGRYVMESRAIARELEKLQPEPSIQLDSPQVQKTFDLLGQIWPPLRSIVLPRVPVMLLPPRSAEYFERTREEQFGKPLAEVAETDGAGEGPWQEAEKGFQGIKALLAENEGPYVVGKEASFADFVLAGLWRMLERVDRDGDVYGRIMKDETFQKHHDACRRWQEKDD*

>jgi|Mycgr3|103524|jgi|Mycgr3|103524|estExt_fgenesh1_kg.C_chr_30003:17-242

MSSKIILYDLPCKTAAGQPYRCWSPNVWKTRLVLNYKKIPYQTEWLKHAEIEPTLSALGITPTPVKYTVPAIKQTDGTGVMDSAAIAPVLEKHVPQPSLHLENNLHNQLGPLVGRSLGPLMPVIYAQIGRAIILESYVEEWKKGKEAAFGCTMEEYEEREGGEKAWKTAEPGFKAMGEFMQQNKKDDGPFVLGSQVCYADFVLAALVECVSKLGGGLYERIVALEPQLKALHDACKPWLENDQ*

>jgi|Mycgr3|69648|jgi|Mycgr3|69648|estExt_Genewise1Plus.C_chr_30719:11-137

MSTLSTTALGLALPREYGYVVTVTAATFFLGLWHGFRVSMARQPAGIKYPKAHADTADMSSATPEQKQAMHIFNCAQRAHGNFLENHPGVAIAMLISGLKWPVVTAGLGATWVVGRIVYQIGYTSKTAENGKGRLPGYILGAIPQLALYVMAAWTGVSMVR*

>jgi|Mycgr3|48689|jgi|Mycgr3|48689|e_gw1.10.898.1:58-258

MPAATTPAVADVPATKAPVAATAPATTTPATTAPATTTTAATKKAEKPIKLYSHAGGPNPWKVAIIMNELSIPYETILKDFADLKKEPFEAINPNGRVPAIEDPNTGATVWESGAIIEYLLETYDGAHTLSYTDTTDRIESRCWFHLQLSGQGPYFGQKAWFTLYHPEKLESCQERYGNEIKRVIGVIDLHLKKTGNEYLVGSKCTYADLAFVPWHWLILFPPHLMGGDFAAEWEKEFPAAWAWNERLQARPSVAKAREERKTAMGQ*

>jgi|Mycgr3|105887|jgi|Mycgr3|105887|estExt_fgenesh1_kg.C_chr_100070:11-210

MSKPIKLYSHTLGPNPWKVAIILEELKVPYETEYMDMSVLKQEPFINVNVNGRVPAIEDPNTGITLWESGAIIDYLIDTYDKSNTFSYSESPQKYYEKQWEHFQMSGQGPYFGQKAWFTHFHSEKNITSAIDRYGNEIKRVLSVIDLHLKRTGQPYLVGDKVSYADLMFVPWNMMFGFIMSEEFDQEWKSSYPKCYEWHQKLIARPAVKKVLDTKAAKLAESK*

>jgi|Mycgr3|80402|jgi|Mycgr3|80402|fgenesh1_kg.C_chr_4000157:2-108

MIVIHHLGISQAERIIWLCEELSMPYEIVNHTRDPVTSPQSLKSVPGNGLGKSPFVHDTETGVNLSESLAISDYIIYKYAGGKLALKANDPHFADYLYWYHFSNSTMQANFVTSMFVNNSGLAPDHPMQHFADQRLEAGQREQMACG*

>jgi|Mycgr3|42293|jgi|Mycgr3|42293|e_gw1.5.1424.1:1-108

MTSENPIVFYDIASDAPRRTFAPNPWKTRFALNFKGVPYRTEWTHMPNITSVREKLGVPANRTLPDGTPYHTLPVIHDVARGEVIGDTFEIALYLDRAFPDSPSLFRPSTTGLTAALNQHIDGLFTKHVGLCSTMPFDPSVKDECMAIFAKRAGVKSLEDVKMTPKQREAMFVSFEAALGELAKRSGRQGAAMFLDGEGPAYGDFVVGAWLKMMEASMATEDWTRVKGFQGGFWGRVVDALSPWTEIK*

>jgi|Mycgr3|100643|jgi|Mycgr3|100643|estExt_fgenesh1_pm.C_chr_70022:15-207

MSSNLKPLVLHAHGTGPNPYKIAAALEFLDLPYEVKLWQFGDAANGVKGPEFLKINENGRVPALEDPNTGVTSWESGAVMNYLLRVYDKENKLGPRGNSEQDRVDFEKWEYFLLSTLGPMMGQVNWFRHYHSSKNEDALKRFEEQAYRCFGVIDAQIKKTGDKFILPGSTPTAVDLHVYSWVYQHSYAGLSMDNYPSTKKWLENVSALKEIKAAYEKVPKGKEM*

>jgi|Mycgr3|35698|jgi|Mycgr3|35698|e_gw1.2.2033.1:64-238

MGIEISPWSLIRVVQTAWKCIPASDICTANDITADQTSYINYIKPIILAEELKVDYTMSIIDTKTEWAYRIHPERMVPALRDYDLATGQVARVFESTACLYYLAEKFDTDGTWTGRGPAERAQVHSWMALQTAGLGPTAKFWLYFLRGYPTRESPVQLPRTIDKFREATMKQWDLLDARLSQPEHLYIALPGRPTLADVAFLPFAMPAIFEIFGVDISRWPKIAAWSTRMMERPAVKKVLDMAPNIGQ*

>jgi|Mycgr3|94490|jgi|Mycgr3|94490|fgenesh1_pg.C_chr_7000293:6-176

MSAPKLFLYDHPVSSYAQKVRMALRHKQIPFEKETPKNLGSGEQNDDFASANMRMEVPALVDGDFKIFGSSAILMYLEDNFPTTPKLLPESPQARAEARMIEDVCDSQYEAVNWCIGEIKWFERATGPEAERLLAALVDQTKQLQVWLDNLLGSKPFFSGDSVGYADFAVAPIVNRSVHYGGAFGPADGSALQQWHARVREIPVVRETFAEMVEGTKMMSAAGPNSFKPHGGRRREYRDHRLEVLIKIGGINIVQKGLEDDNIRFSWPQPK*

>jgi|Mycgr3|78009|jgi|Mycgr3|78009|estExt_Genewise1Plus.C_chr_130569:13-225

MTAILYTSAASGNSYKIRLLASILHIPLQEVEIDLQADEQHSEKYLTINPRGEVPCLVDDGKLFNDSSAILVWLAGKYGNAGKQDGPSSYWSKDVGEQAEIVNWLSFANSWIQYGVFTPRAMLSYGGPFNGLGPASNWSKPELETLFKEGVIRSKKSLQVLDAHLAGAKWLALSRPTIADLAVFVYVALAPMGDVSLEPYGHVEAWIDSVKGLDGFVGIEGLKDPLVHVERWR

>jgi|Mycgr3|48689|e_gw1.10.898.1

MPAATTPAVADVPATKAPVAATAPATTTPATTAPATTTTAATKKAEKPIKLYSHAGGPNPWKVAIIMNEL

SIPYETILKDFADLKKEPFEAINPNGRVPAIEDPNTGATVWESGAIIEYLLETYDGAHTLSYTDTTDRIE

SRCWFHLQLSGQGPYFGQKAWFTLYHPEKLESCQERYGNEIKRVIGVIDLHLKKTGNEYLVGSKCTYADL

AFVPWHWLILFPPHLMGGDFAAEWEKEFPAAWAWNERLQARPSVAKAREERKTAMGQ*

>jgi|Mycgr3|77856|estExt_Genewise1Plus.C_chr_130362

MVLQVYLDPCTINCRKVLAGLDMMDVKYEEHFVSFFKGEQKGEDYVKNINPHATVPAAVDGDLKLTESNS

ILMYAADVAGSDAYPKDLKLRADANRWLLWEASVWFGTNYTYLVENVVKELMGGQPDQSVLSAEEPKWNK

AASILDARLGETGKWIIPGPNPTIVDIAVASPMHLHGAAKVPLDQHRNLKRWIADVEALPAWQKTQPAVD

SALLPNKGKEVRASFNYTKDLGSKLTEIYFYEDPKAVNIHEPGDDPFEVAVHNGWDRVADFSVDKEGFAV

KDFNPSFPNNAFNDDSAVREKFYPEVVEFLKKELGAKRVLVFDHTIRTKANQAKPITDQSNTSQRAPVAL

VHCDYTAESGPVRVKQLLPDEADDLLARRTAFINVWKPLNKVEEYPLAMCDVTSSPPEDFFKLYLRYQDR

TGENYVMRRNEKHDWVYFPDMTPDKSILLKTYDSDKQRAQFVGHSAFVDPTSKPDAPARESVEIRTICFF

*

>jgi|Mycgr3|100257|estExt_fgenesh1_pm.C_chr_50231

MSGVKLHFLQASRCIRAAWQLHELDVPYEFTFAERINGTGPAPPEFKEKAKGLGKFPTLEDGLETFYESG

NICEYLCDKYDKQNRLLPAVGDPKRYKVLQWVHASEATFMLHGLAVLYAKWNQKDGDVEKTVAGLSVNIC

KDLDYLESELGKSSGKFIVGDSLTAADIMMQFSVDFVLVRELGTKGKSWPNVDKWLEQCKSTPSYQEAVK

NTGYKL*

>jgi|Mycgr3|69696|estExt_Genewise1Plus.C_chr_30788

MSSTQVPNEIVLFWYPSSPYGRRMTWYLALRNIPYAECIQPHILPRPTLDALGLKYRRSPVMAIGRDLYV

DTRLMIAKLEQMFPPSAEHPALSTPETAGVASLLQKLTIEGSVFREVVKQMPSAVWKDPKFAKDRAEFMP

ESGMKTDGKTTRAEGITHMRAMFAIIESMFADGREWIAGTEKLSLADLEGLWAIDWFIKDLRASTDYFTA

GKYPKLFAWRDRFRAVLKQAREQAPKAVSLKGEDAVKFVTSASFTDQATTIDESDPLNLKQGATVELYPT

DGGGYTHQDHGKLVKLTKDEVAIAIQAKSGEEIRVHAPRWQFRVRAVDAAKL*

>jgi|Mycgr3|89451|fgenesh1_pg.C_chr_1000868

MEESNSQPATDRRVTPAAKKFSIFDVPAPLRELFTRFPLYTYDANELPLNAPTRRTEHSLHIFSTTDDAA

KGRPSFNPSCLKWQAFLRFAGLPFRLVPSSNHASPSGALPFLQPALSSPESSPVPPIPSNKLKKWLAAQK

LGQNIGEVEDIRYEAYASLLDNRLRKAWLYQLYLAPENSPLVHRLYVAPCSSQPFVQMSIAQQLRTAAEA

ELVKSAATNIVSAPDLIREADEAFESLSNLLGKERWFFGQDEPGLFDASVFAYTHLLLEERLGWRHNPLE

EVLQRWPNLVWHRDRVVVRYFEG*

>jgi|Mycgr3|41684|e_gw1.5.463.1

MPPQEPILFLWGLSAWASKVTAYFALRGIPYTHCEQPITLPRPDLASLGINYRRIPLLSLGRDIYCDSLL

ILEKLELQYPAGGAYPSIAATDAKDRALEKLLEKWTDVVVFRSAAAVISTDLDLMKDPGFQKDREELWGR

SWSKETQDALRPAALAEMRANWTFLEELLRDGREWVLGNERKGPGLADIHACWIFDWLLMLPGSFPEDYF

NEKRFPNVLAWRERYKAAIEKAKEAAPKPTEIEGKECVQKILGSDFNDDDLKVESDDPSGLKEGQQITMA

PIDTGYDSADSGKLVGLTNMEAVISTTSKEGGKEMRVHYPRWNFTFKET*

>jgi|Mycfi2|70922|jgi|Mycfi2|70922|Mycfi1.estExt_fgenesh1_kg.C_140008:1-419

MPFGKLYSYEGNPRTTALLAVAKANGLDLELVHTEPAKGVSDDYKKLNKLGKIPTFQGSDGYVLTEAMAIAIYLTSQNEKTTLLGKTKQDYASILRWMSFANTELLPRLAAWFRPLIGRDPYNKKSVDDAQKATAANVKALEEHLTVNTYLVSERLTLADLFTASIASRGFEFFFDKQWRSQHPAVTRWFETVANQDIFKAVAGEPKFIETAIPNTPPKKEKEEKPKAEKPKQEPKKKEVAAAAADEEEDEPAPAPKPKHPLEALGKSTFVLDDWKRKYSNEETREVALPWFWENCNFEEYSIWQVDYKYNDELTMTFMTSNLIGGFFTRLEASRKYIFGAASVYGQTNDSIVKGAFVIRGNEALPAFDVAPDYESYEFTKLDPKKAEDREFVDDMWAWDKPITVSGKTYEWADGKVFK*

>jgi|Mycfi2|54955|jgi|Mycfi2|54955|Mycfi1.estExt_Genewise1Plus.C_11182:4-252

MASPNIHLYTTQTPNGVKISITLEELHLPYEFTKIDISKNTQKEPWFLKINPNGRIPALTDTFTDGEPIRVFESGSIMQYLVDRYDKDHVISYPPGSREAVEVTSWLYFLNAGVGPMQGQANHFFRYAPEKIPYGINRYQNETKRLYAVLDKHLADSKSEYLVGNKCTVADIAHWGWISAAGWAGIDIEPFPHLKAWEERMWARPAVKKGANVPDPYRMKELLADKEAMEKHAAESQKWIQAGMKDDASKHQK*

>jgi|Mycfi2|55413|jgi|Mycfi2|55413|Mycfi1.estExt_Genewise1Plus.C_12290:11-273

MSTNGPSKELDPNQGGDASKKTYHKKATGEALQTAVSHAASKDLKLYGSCFCPFVQRVWISLELKGLDYEYVEVDVYRKPKLLLDINPRGLVPALRHGAWGCYESTVLMEYLEDLHEGRALLPEDPKSRASARLWSDHINRHIIPLFYKYLQAQEPDDQVKFAGDLKGQIAKIVEVADQDGPFFFGSEMGFVDVQFAPWIVRLEKVLKPYRGWPDAEPGSRWDRWVRSVVEAEPVRKTTSTDELYLDSYERYAENRPNTSQVRDAINSGGGLP*

>jgi|Mycfi2|163170|jgi|Mycfi2|163170|estExt_Genewise1.C_31006:1-268

MSDDRPTGLIAKSGLELLTFGTPNGHKATIVLEELKEAYGKPDYVFQSINIGQNIQKEPWYTEKGPNGRIPVLVDHDNGDLGIMEGLAILSYLTRHFDPEHKFSFTKDPELSLCEQWVAWQHGGLGPMQGQANHFYRLAKERIPYPTQRYVGETERLYGVLDIRLKGRDYLVGDKYSIADIANFSWVNVAYFAGVDLDQFPNVYKWWERINARPAVQKGIAIPNKPTFTNEVYKKRLQEEPEFKQKDDELKEICKKAKEQYNYKFSAP*

>jgi|Mycfi2|44255|jgi|Mycfi2|44255|Mycfi1.e_gw1.26.180.1:13-349

MASTSLPKEWHSGPDDSFHGKITDDGPFNPEKDRYHLYIALFCPFAHRVNLVLHLKQLHKYAGIETSIVRPYPKGDAKGWPGWRFNVEDKKDEADKADYEGATVDKLFGSKYMHELYFKADKDYKGRYSVPVLWDKKLNTIVNNESHELLRDLQTAFNSLLPKDLADITLYPEPLRAQIDHLGEQLQQHLNTGVYKAGFAPDQATYEKNLPPVFAILNKLEKLTSSNAGPYILGPKLTEIDIRTFCTLIRFDVVYVQHFKCNLGMLRYSYPTLYNWLKGLYWNLPEARDTTDFRHIKENYTKSHYDINPRGITPLGPWPDIDRGFERDWGMVGVGGIEMEEVLEFEERLGKEVVG*

>jgi|Mycfi2|43687|jgi|Mycfi2|43687|Mycfi1.e_gw1.22.110.1:1-331

ILNWVDPKDKSGEFKRQQSQFRNFISRDPKAEFPAEKDRYHLYVSYACPWAHRALIVRKLKGLEGFITFTSVHWHMGEKGWRFSTKDENEPGENTTADPLHPEFTHLQDIYFSNDKNYQGRFTVPTLYDKKQKKIVSNESSEIIRMLYHEFDHLLPERYAKLDLLPDDLKEEIESTNEWTYNDVNNGVYKSGFATTQEAYSKAVTQLFKSLDRIESHLSSSPGPYYYGDRVTEADVRLFTTAIRFDAVYVQHFKTNIRDIRSGYPAIHKWMRHCYWNNPAFGETTEFTHIKAHYTKSHGQINPFGITPVGPEPDVLPLDEEVPAVKAALKK*

>jgi|Mycfi2|36185|jgi|Mycfi2|36185|Mycfi1.e_gw1.9.686.1:5-230

MSSSNITLYTGQTPNGIKISITLEELGIPYTVRKVDVANNEQKSEWFEKINPNMRIPAIADTFSDGEEICVFESGSIMQYLVERHDPEHRISYPRGSREWVETNNWLFFMNSGVGPMQGQANHFFRYAGEQVPYAIERYQTETKRLYSVLDKHLRDSAHPYLVGEKCTIADIAHWGWIALARWSLGGEDYPKSPLDAFPALRAWEERMFDRPGVQKGRQVPDKHQREMLMDPEAMRAFEERGKAFYRKMEEEKSAKEAGRGQEEKVA*

>jgi|Mycfi2|209463|jgi|Mycfi2|209463|estExt_fgenesh1_kg.C_10034:87-344

MSSSRLLTRQSVATHYLFHPRLATKHIRTASNNIRLARNRLPGIKDSPAGIRSFASSSGKLYATLSKTPIFVLQAKPRGVEMADQPAKKLRTSHPTYELLYHPGIPGRGEFIRLAFEAAGVPYTDIANEDHKNGYAEVQQVCMNDGIESSDGNPPVFSPPALRVRDGEKALVISQTPNILIYLGEKLGLVPAGDEKFYVQQLALTALDLNNEIHDTHHPIAASKYYEDQKDAALEKAKDLRATRIPKFFKYFERQLKWNSSAGQHKYLVGDRLTYADLTVWQILDGAKYAFPKELEVRRKEFPELLGTFYESVKEEEGIKEYLASERRLKYSMGVFRYYPELDRP*

>jgi|Mycfi2|88341|jgi|Mycfi2|88341|Mycfi1.estExt_fgenesh1_pg.C_140375:14-258

MAETTAAAAIEKPKITLYWLEKSRSQRIVWLLQEAKGVDLEIQIFKRGKDMLAPPELKKIHPLGKSPVISIKSQNTPNGLVLSESGALTEYVVDHFAQHLAPKRYQEGKDGQIGGETEEWLRYRLYMHYAEGSLMTLLLIGLFVDQIKNAPVPFFIKPITRTIGSRVEGQFLNQNYATHFAFLDSQLASSPGGGPYICGTQLTAADIMLSFPLIAGKSKIDKSKYPKLGAYMELLEKHEGYLNSIKKIEEVTGEKFAPML*

>jgi|Mycfi2|101422|jgi|Mycfi2|101422|gw1.4.471.1:9-249

VSGNAVASPKITLYTNHQCPYAHRAHIALKELNLPFEEVIIDLSTPRPQWYLDINPRGLVPSIKYSVEGVYDEEILTESAIVAEFLANSFPGKLLPGLRDSPRAPLERARINFFTDTWNNKVSSFWFQTLLAPAGSAEKEAKAEEWFKAVEKEIEPLLSNSAPYFNGSETLTFAEVHAAPFIVRWYALGKHEDLIPTSLIRNLDSLPKFSVWAKAIRENESVTKVVDWEASVDAMRAKVEQFRAKAAAK

>jgi|Mycfi2|187791|jgi|Mycfi2|187791|fgenesh1_pm.3_#_735:9-277

MSRPTHSHADADGHFRRKASSFRNFISKEPGAQYPPEKGRYALYMNYGCPWAHRASAVRALKGLEDVIQMIPTDFDLTENGWTFTGKHGSEEKDPLYGFTGLKQLYLKVEPEYEGRYTVPCLFDKKTETIVNNESSEIIRMFYTEFDDLIAEELRESSKGDQGLFPEHLRKDIEEMNEWVYNTINNGVYKCGFATTQAAYDANIYPLFESLDRLEAHLTSKGTPFLFGDFITEADIRLFTTLIRFDTAYHTIFQTNLKSLRHDYPKLNLWLKRIYWDEGKLTNGGAFKKTTDFQAYKEGREEKGKERK*

>jgi|Mycfi2|36633|jgi|Mycfi2|36633|Mycfi1.e_gw1.9.740.1:3-225

MVLTIHHLGHSQSDRVVFLCEELGIDYTLKKYSRSPVLSPPEYKALHPIGAAPVIEDDGGVKIAETAACFEYIINMHGKGKLWVRAGAKNYPDFLYWFHFVNGTLQPGFSRTMAMTMAGVPEDNATMGRYNQRNQMALKFVDERLGEVEFLAGEEFTAADAMIIFSLTTMREFCPVDLSEYKNILAYVQKIVARPAYQRYLQKGDPDIDIQQFIRGPPPPVFAAFRQHK*

>jgi|Mycfi2|87873|jgi|Mycfi2|87873|Mycfi1.estExt_fgenesh1_pg.C_110046:5-214

MGKSITLYVDIVSPFAYIAYWLTRNSRAFSSIQIKYVPILLGGIMQSTGNTPPINIKNKDKWIGKERVRWAKTFGVPMSDSMPEPFPQPTVNAQRALCYLYNKSQDDVIKGIDALYEAFWVKSKKFNDMGTIEEALGTKFGKSEVQEILKGMKGDEAKKALRENTDEALKEGCFGLPWFVAENEKGQKEGFWGVDHIGQVMEFLGVELESEGGRYRSML*

>jgi|Mycfi2|37457|jgi|Mycfi2|37457|Mycfi1.e_gw1.11.507.1:7-237

MSHNLYTDETPADVKNAKGLHLITQNTPNGQAVQIFLEELKDTYGTEWTTTIIDISTNEQKKDWFLRLDPNGRIPVLIDNTQSPPFTVHETSAELFYLLKFADKQDKFGFTNDLERNQALQWTFFWHGSGAPYQGQVNHFTRAAPEKLPYAITRFRNETLRVFGVLEIQLSGKYTGEPKEYLAGNGKGKYSVADIKTWPWVKNWEKSGFTQEEMDNFPHLLKWIDRVAERPAVKRGIGEGYLKK*

>jgi|Mycfi2|7829|jgi|Mycfi2|7829|Mycfi1.gw1.23.108.1:4-167

MSKLRLWLSPGACSLAPHVLLQASGLAFEILVIDITKGGFPEKYRAINPKKRIPILEFDGQVLTETTAIMTLTSRLAPEKHLFGKTDWDVARTYEWLNWLSGTVHERGLGALFSPEWYSDDQSAHDGIRKKSREWVEVCFGLIEERLQGVGGEHAVKDVFTAADVFL

>jgi|Mycfi2|136482|jgi|Mycfi2|136482|e_gw1.4.469.1:14-247

MSKEIIFYDLPSRGSKPTAWSLNPWKTRLVLNYKNIHYKTQWVEYPDIATTFKSLNIPPNDPALNPNAAYSIPAIALPDGRHIMDSLAIAHELENLYPSPPLTFDKADQMQSAVLKVNKALAPIIIPRVPELILNDSSKEYFLTTRAKRFGMPLPELAKTADVEKCWEGAKEGLREISALLNGTHDGGSFVLGGDQEPGFADFVLAGFWRFCERVDVDGDLWARLKGFDGKFGRHRSAVAKWLERDD*

>jgi|Mycfi2|213777|jgi|Mycfi2|213777|estExt_fgenesh1_pg.C_11897:1-290

MQLYTLGPAFGLPSIDAECSAAVALLQLYARGKYEIIPTHDTRTPLPRLIDGTARIFGFNNIVRHLVDKRIAISETSLDKGQRALCTAIASLVESQAQTLLDISLYVGFENYRLATRPAFSKILPWHANYILPPTRRSAARSRTEHLGISSIDVDDVHEDMSNRPEGFDGVGKQPQFEAETQKRASLLLPRKETVRSLLQRPEHAAIFKLNALADNFFEPLQEILGDNVYAVGGELAAVDCLVYGYLSLMLYPELPQDWLSATMKRKYKSLVAYVERIHAKFSLNTNVEAVMDLSKCESHAAVLAKREASKTVLPWSSPHTSNASEALQTLCTGIWDQIPLLGDPNKLQLWHAKKLPFLQSNLTFLLLSTTTLLALAGYLSVSTGLLLWPRGEEVHIFGRRRMSDLGHLGAALAGVNFISRPSAPQR*

>jgi|Mycfi2|142152|jgi|Mycfi2|142152|e_gw1.8.1914.1:1-193

MSSIKLFFLQASRSIRTAWQLEELGLNYELKFAPRENGVAPPAFKKEAGGLGKFPSLEDEGIMYYESGDPQRYKALQWVHASEGMFLLHGLACLYAKWFQQDGDVQKTQEGMSKNLIKDMDYLQSELEKSSGKFLLGDKLTVADVMMHFSARFIIVRELGTMGNRWPKIEEWLDLCEAQPAYQAAVKKTGHQL*

>jgi|Mycfi2|145126|jgi|Mycfi2|145126|e_gw1.10.1131.1:10-249

MASHPDADLHPVATGPAKSIVDAHQKEEPLKLYSGWFCPFVQRVWSIFEEKKIPYQYIENKSQSLLSLNPRGLVPTLQFSDKPLFESTVICEFLEDAYPESTPKFLPEDVYLRAKTRLWIEFFSRSLVPAFHRFLQVTRVSNFEDKQLESETIDKDLSKEDEDLSSNEEGEEPTLIDFAVAPWIVRLWGSDQFKGGLGIPGEGKGGRDEVVWERLRRWKMALENRPSIRDTTSEREYYLPIYQRYAEDRAQSELAKSIRKGQGVV*

>jgi|Mycfi2|209914|jgi|Mycfi2|209914|estExt_fgenesh1_kg.C_10495:1-220

MPSNKITLYDLSSKVSKQCWSPNVWKTRLVLNYKNLPYDTEWLTHITIEPTLSSYGIPPNEPPKQGPPQSAYTLPAIRLPDGTFVMDSAKIAPVLEEKYPENSLRLDTEMHVKAQEVLGQVAPPLLGVLFPMIMRRMIPEDVVPTWRAKKEAGFGMTVEEFEAAKGGEKAWEAVKPGLENLSKFLKEKKKDEGPFILGGQVCYGDFVIAGLAEACKRIGEEEIYSRLVRECPEVKSLHEACGKWFEKDT*

>jgi|Mycfi2|191416|jgi|Mycfi2|191416|fgenesh1_pm.12_#_4:11-208

MEILLARYMLTPNPWKVLIILEELQIPYDLKEIPFADIKQEPYISLNPNGRVPAIEDPNTGITLWESGAIIEYLIETYDEAKTLQYTSFPEKHLQSQWSYFQASGQGPYFGQLGWFSFYHPEKLPSVLDRYKAEVRRVLGVIDAHLTKTRQEYLVGEKLSYVDLMFVPWHEIAPKLLVGEGFLEEWEKTWPKTWAWHQKLMQREGVQRTLALRESK*

>jgi|Mycfi2|37449|jgi|Mycfi2|37449|Mycfi1.e_gw1.11.286.1:13-210

MSPKPLTLHAHGTGPNPYKIAAALEFLQVPYQVKLWQFGAAKNGVKGPIFLKINENGRVPALEDPNTGVTSWESGAVMNYVRRVYDTQKKLGPRGSSEQDIVDFEKWEYFLLSTLGPMMGSLNWFRHYHSSKNEDALKRYEEQAYRCYGVLDGQLEKHGGPYILPGNTPSAVDLHFYPWVYQHGFAGLSLESYPNVKKWLEGVASLPEIKAAYEKIPKGEVV*

>jgi|Mycfi2|152707|jgi|Mycfi2|152707|estExt_Genewise1Plus.C_31188:5-132

MSVTIELPKEYGYVVATTAATFFLGFWHGLRGGLARKPTGLKTPKAFADSGDIAAAKDKEQAKAMHVFNCKQRAHAHYNEVQPSTALAMLIAGLAYPRTTTGLGIGFIIGRIVYAIGYSNVDKPNGSGRVLGFVISQPIALALWGLAGWTGVKLVL*

>jgi|Mycfi2|36038|jgi|Mycfi2|36038|Mycfi1.e_gw1.9.736.1:2-203

GPNPQKVGLILEELQIPFDVKIIESASQNSDRHERSSDEGAPPVEDKNTGMIILEDTKKYITLWESLAIVEYLIQTYDKERKLHYAPDTQKWWMCQQWLAYQMGAQSPFFSLKAWFSNQHNDKVPSVIQRFSDDVVRISSILEHYLKRNGTGYLVGEKCTFADLVFVPWNNKIGKSQALEIFQKYPCYHSWHQRLAKRPAVQRILQNKDISIQ*

>jgi|Mycfi2|25832|jgi|Mycfi2|25832|Mycfi1.e_gw1.1.607.1:240-272

MAQPLVFFVGAASSGFGKGVALEALSRGHKAIATSRNSSKLADLKEKGAVTMDLDVTQPLSEIQAIVAEAHKVYGRFDVLINAAGYILEGAVEEASPEETYDSFNTNVFGVINLTRAITPYMRQQHSGTIAHFGSVGSWHGAAGAGVYVAVKWAITGFSESLALELKDFGIDVISIEPGYFRTGFLNPGARQLTAKRLKEYDATSVGVVRKVFNEQNNKQLGDIEKGCKVIVDVLTKKDGKDVPMRLVLGSDAYQMIKEKCESTIALLEEWKDVTCRTDHS*

>jgi|Mycfi2|211935|estExt_fgenesh1_kg.C_70055

MSSKLFLYDHPMSSYAQKVRMALRHKGLAFKKETPENLGAGNPNTAFKSANPRMEVPALIDGDFKIFDST

VILMYLEDAYPDTPSLFPEGGNDAKAKAEARMIEEMLDTHYEAINWALGEIGWFKRAEGAEAERLKAAAR

EQIQQIQEWLATKLGSKAFFSGEKVGYADFAVAPILNRSVINGDGPAPGSALHAWWKRCAEVPAIKQTWD

EVAEAAPKMAAMGPEAWKKGSGRKREYRDHRLEFLIKNGGIGIVQKGLEDDNIRFSWPQPRKGGKL*

>jgi|Mycfi2|62532|Mycfi1.fgenesh1_pm.C_scaffold_1000189

MPYTLHTNWRSTCSSRLRIALNLKHIPYKPIYVDLDRGEQHHESYLAVNPSGTVPCLQSEDGKVLITQSL

AAIEFLDEVHPDSTPRLSSRASFTPLTSIHHCHRLTSTRVSKDMVALGGFGERIFEESLSEGTCCVRSSV

IGGKIGILLVISSLWLMFAWFLLFGRLGCLSLMLRLFFPKIWSVYLLMMERRVDAEAHWKQQPDCPGGDF

T*

>jgi|Mycfi2|28422|Mycfi1.e_gw1.1.4214.1

MPNDDRPDAPNERAGNAFAPTPSSAKRSIFALPTSIKRVFDTFPLREFPANALPIRAPRHREEHVLHVFA

TDEGAKLSKPSFNPGCLKWQTYLLFSGVPFKLTSSSNHASPSGSLPFLQPAGTGTETNDTPQPIPSSKLK

KWLASQGFEKISESSDIRYEAFASLVDTRIRKAWLCQLYLAPHNAELMHRLYVAPCSSHTLVQMAIASHL

RSAAEAELVKSAASNIISTFDLLKDAEEAIEALSILLSDDDYFFGQQKPGLLDASIFAYTELILDDRFGW

KYNPLLEHLSKHENLTKHRNRIRELYF*

>jgi|Mycfi2|133216|e_gw1.2.3384.1

MYGADVAKNSAAYPTDLKLRADANRWLLWEASVWFQTNYVYLVEYVVKPLLGAQPDQNIIAKEAPKWHKA

AAILDARLKETGKWILPGAEPSIVDISVASPVHLYEAQQLPLEQYSGIKNWLKELEKLPAWQKTQGAVDK

ALLPNKSSEVRAEFAYTKDLGDKLTELYFYEDSKSVGIHEPGDDTHTMSVKSAWGKDWDVDINGFALKDF

QPSYNHSWEDEEKVRTEFYPEVVEFLKKELGAKRVLVFDHTIRTRRNQNKKLTQENNTSQRAPVRLVHCD

YTAESAPTRVRQLLPEEADHLLARRTAFINVWKPLGPVKENPLAMCDVASAPPEDFFKLYLRYRDRTGEN

YVMRHSDRHQWYYFPDMEDTQSILLKTYDSDTTKAQYVGHTAFDDPTSAKDAPIRESCEIRTICFF*

>jgi|Mycfi2|28695|Mycfi1.e_gw1.1.2704.1

MPPTNEPILFLWSLSAWASKITAYMALRKIPYSRCEQPITLPRPDLTALGVRYRRIPLLSCGLDIYCDTL

LILQKLESLYPENTPTHKKISADTSTEKALEYLFEKWTDTVVFKSAAATISSDLDLMQDPKFQSDREALW

GRPWDKATQDSLRPSALAEMRENFEFLENVLSDGRTWILGRGTEEKEGGPTLADIHAAWIFDWLGMLPGS

FPEEEFNARKFPGTVGWLGRYKTAIEKAKESAPKPTELESVECVDRILNSGFEETELEVEENDLSGLKAG

DEIKMAPVDTGFGSSDVGKLIGLKRNEVTISSLTQQGGKEIHIHYPRWNFSFEKI*

>jgi|Mycfi2|151894|estExt_Genewise1Plus.C_22912

MSYKKGLLNGHPGDIILFTYPESVFGRRMVRYLNLRRLPFSQIRVPPYMPRPILQERLGVNYRRIPIMAI

GRDIYIDTRLMLAKLETFFPENRLGADDDFGSGVEDLLENLIIDGGPFWRTAGCIPPTAPLMTSEVWTKD

RSDGSGGAFTKQALLENRSWSISQLRIYYDIVEKMLRDGRQWILGGDNPGLAEIHAGWVFDWGLNMAQDM

GDGSNGATADMKRALSDVEFPKLHDWVKRFRDLAEQAGEDHAGGGRLQEGADAEDQVVDRILAADFTEQE

PYFDESDVLGLQRGQRVTIAPVDFGFTHKDEGTLVGISKNEVVIEVQVPQGKGSLRLHYPRINFKILRMS

*

>jgi|Mycfi2|152431|estExt_Genewise1Plus.C_30674

MSELGLTLYTYFRSSCSCRVRTACHLKDIPLDYKFVNLVAGEQKAGPYIDQVNPSGLVPALVVRDASGKT

VATITQSVAILEFLEEAFPTSRPLLPRASQPLLRARVRELVGIIACDIQPPTNLRIVKRLNGLGVTNQQW

FQEMMESPLTAYESFLKGTAGKYSVGDEVTLADVCLAPAIENALRWEVDLSKVPNVVRVFDALKVLPEFV

KGDWKHQDDTPENLRAP*

>jgi|Mycfi2|82743|Mycfi1.fgenesh1_pg.C_scaffold_16000318

MPSLPPVIAWDYQFAPNAQKSRNYLYATKTPFKVCEQPFFIPRPILRNLGITYRRVPVLSIGKDVFPDNA

SFIEAMQSLLEQQGKALKTSPADRSFEAWGYRSFWVALPCVPVDFNNKQLQNDRKDLFPLFGRADYSRLQ

TNAASELRSLIETVENDFLSQGPWIAGQECGLADIHAMWMLKWAMKTLDMEKQPGLGRDEWPKFYHWIES

IPHHAPELEQNDKINEQEARNLLFASEYAVPHVGIDPKDPLQYKAGDELYVEPTDAEPGQHPQHGKLVGL

NTNKVVIELENGLRVHFPRIGYIIHRSADLPVKGVVEKVMDKVGLGQSSSVSAI*

>jgi|Mycfi2|188248|fgenesh1_pm.4_#_336

MMPRPTLRDSFNLTYRKIPVLAIGRELYCDTSLICEALEHFFPPSEGYQSIYPEALDGRTYRPLIRGFAS

YWTDRPIFRIMTGLMPASIWRSSFGTDRAQLIGHKLDPDKLEKKLPENLTKYDHQLSMLEPLFAETDGPW

IFSTPTPSLADITLYYQFRWGNKIASGEGVYSLTMKDARDSKENGSAPVFNPQRYPGIHAWYKRMERYFD

ELPLTEEDKSNDLESVLEQMKKAPTPGPKSMLLPTPRSVHKELSEKIGLKEGARVSVTPSDTGRADPTIG

TLVALSPEEVVIKPKQLEKAAAVDVRIHFPRTEFVIRPVNEANL*

>jgi|Mycfi2|43451|Mycfi1.e_gw1.22.262.1

MAATQIDDKVKSLIAKSFPNASDQENEPAKLSAAIFTSVEYTPAEKTEIEQWSTTASHLASSNEDAAKTA

ERLSHLNTHLASRTTILGSKPSVADVAIFSRLAPVVKDWSPEQRTGEQGYHHIVRHLDFVQNAELFGLKL

DAGEKVDIDPSKVVAPIKPIDAKAEKERKKKEKAAAVAAGADSAAAAGGTVTSAAGEDTKTAENQKPKKE

KAKDRGESIAAAVGASTPTEKKEKPKKEKQPKQPKAPAAPEKPFSPALIDLRVGHILKAEQHPNADSLYV

STIACGDAPGTDNTSEYEGKVVRTVCSGLNGLVPLGEMQGRKIVAVCNLKPVTMRGIKSAAMVLAASPRV

APGEDDHHAGPVELVTPPADAEAGERVYFEGFEGEPEPVLNPKKKVWEENQVGFTTTEDKVVAFEPTKVE

KLKESGKTEVAKLRTKQGVCTVKTLTGATVR*

>jgi|Lepmu1|1836|jgi|Lepmu1|1836|Lema_T087920.1:1-414

MSFGKLYSYSGNPRTTSLLAVAKENGLEIEFVDTEPAKGVSADYLKLNKLGKVPTFEGADGFVLTECIAIAVYLASQNEKTSLLGKTKQDYATILRWMSFANTEVLIPLGGWFRPLLGRDPYNKKNVEDSQKAALKAVHVLEEHLMTHTYLVGERLTLADIFATSILARGFQFFFDKQWRDANPNVTRWYETIYHQPNYSAVAPKFEFITEAMKNVAPKKEGGEKKKEQPKAAPKPKKEEAEEEEEAPAAPKPKHPLEALPRATFVLDDWKRKYSNEETREVALPWFWENANFEEYSLYKVDYKYNDELTLTFMTSNLIGGFFARLEGSRKYLFGACSVYGESNNSIIKGAFLVRGQEALPAFDVAPDYESYEFTKLDPKSKEDQEFVNDQWSWDKPLIVDGKEYPWADGKVFK*

>jgi|Lepmu1|1180|jgi|Lepmu1|1180|Lema_T011800.1:1-332

MASKEVHKYGTNDGWHGVIEEGGEFPPEKDRYHLYIGLFCPFAHRVNLVRHISHLQDILPISIVRAYPKDEAGWRFDETYPGSTPDHLFNSRFLHQLYFRDDPAYTGKYSVPVLWDKKAGRMVNNESLETMKWLPRAFSSLAQEIPTIRTLDLYPSALRGKIDQITPWLTSLICSGVYKAGFVTEQSAYDANVVPIFAALNKLEAIVHAHGGPFILGSQMTELDILAYPTIVRFDTIYVQHFKTNLGMIRHDYPVLHNWLKGLYWNTEGFKESTDFVHIKENYTKSHKDHNPHGITPMGPYPDVEEGYEEDWNKLKPGQVRHPKVLDAMSKL*

>jgi|Lepmu1|7127|jgi|Lepmu1|7127|Lema_T120720.1:1-432

MVLELHVWGPAFGLPSIEPECIATIAYCQQVLAKGQWSLVAEHVPSVGITASGFEDIVAYLRNHPTAATHDLDAKLSSRQRTDRIASVTDRIPGSTATSLIDLLLYVSAENYRTATSTAYTAILPWYANYTVPPRRRELAKARTSHMGLGSLEVDITAEEAFAPGRGTASSEYEAAKRAAGIPSDGTPRAMSMGRGKGFGGLLSGPVYAARFRLDAISDELLGPLSDLLGKHDYLFRGSAPSSLDCLTFGYLSLLYYPSLPQAWAKETLEARYPRLVEYMRRIRLHIFQDDVTDPSKVWSVMTGSADASRGMLLPWRPRRQALASSAVACTREILGNVPLVSLAFQRRSFVVEERQSSIAQHVKSELWSPLTVNALACTTAAFAIGLVTLAVHHRRSPREGALIFWALRPSVGLGEAGDILSVLAHQMPNGASLSLL*

>jgi|Lepmu1|11354|jgi|Lepmu1|11354|Lema_T072650.1:54-304

MAFTDIDLLRRNRSFKQSRIRNHSWSVIPRLLSTLLQIPLCKIQLNTLQHETKMSTKSDITLYTTQTPNGIKVSILLEELGLPYKVEKIEITKNTQKEPWFLAINPNGRIPALTDTFEDGQTIRLFESGSIMQYLVDRYDPEYKMSFPRGTREYYEMNNWLFFQNAGVGPMQGQANHFTRYAPEHIEYGVNRYQTETRRLYNVLDKHLASDNRPYLVGEKCTIADVSHWGWIAAAGWAGINLNDYPALAAWEERMAARPGVEKGRHVPDPHTIKELLKDQAKAEAEAAKAREWVQSGMKDDAAKHGGGKVNGKI*

>jgi|Lepmu1|9847|jgi|Lepmu1|9847|Lema_T051760.1:519-811

MGTALRFRGSSHQPLQPFLRALTSWSLFCNLHHIPVAIREPGSLPGDLFQTSTTALGSSASSPVLQQQLRSLQQHPGALHTLHQSPDGTLYPQQTVPELQDLHAHNVYHAQQQQQHQHQQQQQQQHHHHHQQQPYALNGPPPPPPPPTAHQLPVHHHQYEQHGLPSSASFPPYPATATYQHATSPRLFVNAPPPPPPLQGPAQQHYHSPQLSTPLPPQTQLPPQRPIQQLSLPAPLPILTLPQSHPHVQHAQPVLQQPQAPDGDESPFTNHGQFQGLKLIPNPPDLDLWRERLFNVDDMITLTEEEFQTYFPHIDNVYSHRSTQRHKRKRFVSHYWDCRLKGRPPGTKKSTDPDKKKRKRVARERNLCDVKIKITEFFDQREYEEQLGHRPPVASELESTSPMTAGSMVGGNLGQTQAFYGQPHQMLPQHQVGSWDMPSNMGEPSMSMSHYAPGPPLSPGPPPKKFYTFQRVNGNGGNGKGDGVAGPHKHTLEESDRVKKNSVLRWQAKMEKDDRKKVQGGDATKKTYHKKATGNALTTVKNHSKEDDLKLYGSAFWQVSLLSYSYQHLLPTHDTSSQSRSTATNPQPSPFVQRVWISLEYKRLPYQYIEVDPYKKPQSLLDVNPRGLVPAIRHGPTWSTHESSVIMEYLEDLAAGPPLLPLDPQSRATSRLWTDHINRHIIPLFYKLLQSQAAPDQHTHAANLAAQIAKLIDAAHPTGPFFLGPNLSFVDVQLAPWILRLSRVLTPYRGWPAPEEGSRWRRWVEGIEAERCVRVTTSGEDLYLDSYERYAENRPGTSLLADAVNAGGGLP*

>jgi|Lepmu1|5472|jgi|Lepmu1|5472|Lema_T014460.1:1-239

MASPKLIVHHLQVGQGERIPWLLEELNLPYELKLYQRAPLLAPPELQAVYPIGASPVLEDLTDPSHPIKFAESGAICEYVIHKYGNGRLALKPEDKNYADYLYWLHFSNATLNAGIFRRAMARNMVGEEDMRYKGNDARVVNTLEHVNNRLKENKWLAGEEFTAADIMTGWCFTTMRKFEPIDLTPYDGIMAWLKRIGERKAYRTAMAKSDPDLDIEAGLSVKGPPMHEVWAKVMGSKPVECFSSGMACADVETTIAGFAPAYAGRSITVSSSRDYRCELHDKKVLIPYQNLEFFHQFVVTIRLDLMNGIWYGSYRKMVPSIDRNISDSADDADEALPDPLRRTQERATRHSSHDTMSM*

>jgi|Lepmu1|7228|jgi|Lepmu1|7228|Lema_T121730.1:4-234

MAENAPKITLYTNHRCPWAHRAHIVLRELGLEYDEVIIALDKPREPWYLEINPRGLVPTIDFNGEIITESGIVVQFLADAYPSHVLPAAGSIEGALKRARINFFVDSWFSKVGSYWIQILKQDNAEEKERLGREFVETVAKEIEPLLKGAGPFFGGSNKITLAEALVAPFILRIYALAKHGRLPQSIISGFDALPNFSKWAAEVMKQDSVTYIWEEESTVAVLMKRVETMKAQAKAGSK*

>jgi|Lepmu1|4326|jgi|Lepmu1|4326|Lema_T099980.1:5-267

MAAASGNKKARTHPPYELLYHPGIPGRGEFIRLVFEAAGVSYKDVANESDEGTKSVYALVDPASTGDKDGNPPPFALPALRIPGEGKNGNALVIYQTPAILAHLGDKLHLAGSDEAQRAWVMGHALTALDLNNEAHDTHHPIAVSDYYEAQKEEALKKAKVFREQRIPKFLAYFERLLTGNREQGDGKYLVGNQLTYADTTLWHVLSGLHFAFPKEMEARQKDYPTLFQTFYPAIQEINGLKEYLTSDRRGPFSMGVYRHYPELDRE*

>jgi|Lepmu1|1901|jgi|Lepmu1|1901|Lema_T088570.1:3-260

MASNQQGAKITVYWLDKSRGQRIVWLLEELNLEYDIKVYKRNEAGRAGPELKKVHPLGKSPTIGITPAGSDKEIIIAESETIVEYISEHFGKQLIPNRYPEGKEGEVGAETDEWMRYKACEFLMDYAEGSLFTILIVALISGKIKGAPVPFFIKPIVKGVAGNIDSSFVDPELKNHFDFLEDYLSKSSGEFFAGSTLSGADIMMHFGLEGACQRVPLSETSYPKLYAYMRRLQQRDAYKRAADRVTEASGEKYVPYSDLKK*

>jgi|Lepmu1|12070|jgi|Lepmu1|12070|Lema_T063220.1:1-220

MPAPKITLYVDIISPFAYMAFYTLRNSSVFKGCEITYVPILLGGLMKLCGNTPPLMVKNKKQWFNKDRLRWASQLNVPMSQATPPEFPFNSMHIQRALTSLALARPELLESAVALFWEHTWVLWREPDKAENILALMKTVVGGEEEARRIVEAMQSEEVKRALVANTEQAFKDGAFGLPWFVATNAKGETEGFWGVDHIGHLCDHLELEREDGKAWRALL*

>jgi|Lepmu1|9904|jgi|Lepmu1|9904|Lema_T052330.1:1-241

MSESEIVLYDLASKHGKAWSLNPWKTRMVLNYKSIPYRTHFLEYPDLAPTLLSLGIPPNPKSTPGYAADYTSPAVRYADGTYGMDSWPIAHTLETQYPEPSLHLEDPIVVQVRDAIMQLFVPLRAHLIPKVPGVLSERSQEYFYRTRRERFGRPLQEVGAEAGEAEWETARKPAEMMGDMLRRNGGPFFLGDKVSYADVIFVTALHCFKCVEEELFTKLVALDPAFSKIYEASKQWLEKDD*

>jgi|Lepmu1|596|jgi|Lepmu1|596|Lema_T005960.1:1-129

MPADIAERTQVRVWMSASEGTFLIHALAIMYGSGAAPDAADKISKGLSPQVHRDFDWLESELKKGGGKYLVGDHLTAADTMMGFSVAFILKMGLGTSGKKWPAVEAWLANIESTEAYQRAVNKTGHTLG*

>jgi|Lepmu1|6816|jgi|Lepmu1|6816|Lema_T117610.1:5-192

MAGGKITGYLDCVSPYSYFALLSLDKNRALLERHGVEIDIIPVFLGNKPPWTLPAKGAYSSYDSARAKRYFGVPNVQTPSFFPILSLLPQRALGYIKEAHPDKFISVFLSIYAAMWQDGQDVSKPELLFQILHQHFSDAEVDRIMKSAKTAPYKQLLNDKTKEALDRGAFGCPWFIVRNAKGEEEPFFGSDRRPIPLSTWSRCMEIDCRRGGGPGHVELTPPREL*

>jgi|Lepmu1|8286|jgi|Lepmu1|8286|Lema_T031800.1:1-141

MQGQANHFNRFAKERIPYGMQRYTGETERLVGILDHQLKDHDYLVGDKYSIADIASFGWVHMLAFTGVDMEQFPHVKKWWARLLERPAVKKGLDLTGKSQFGNDVYWQRLKEDKEFAESERKLMEQIKAAKEQYGYKYSSP*

>jgi|Lepmu1|5957|jgi|Lepmu1|5957|Lema_T019310.1:51-298

MVESTVKTALARQRVFVCLVGHATSKSRHCIPSTLITIWGRMAYSFRGTIPSQRFPAEKGRYVLYFNAVCPWSHRAIIVSALKGLGDIIQTVEVDARDQVRGWHFSGHRGPHCDPIIGAKCLKDLYLLADPEYSGRVTVPLLWDKQQQTIVNNDSGDMMRMLIDAFDHLLPVEKRELSKGKAALRPAHLISKIDALNDWVYDTINNGVYKVGLTRSQAAYTEHITRLFKSLDQLETHLTEPEHHPYLFGPHITESDIRLYTTLIRFDIAYYPFFRCNLKMIRYDYPHLHDWLRRLYWDNGKDTAGGILGFKRVTSSRSSEVMHASMYGTGSYPLDQHHTLCHCDLSLKHAHIVYWCSLAFSISPWLLQLACTTGRLWTFTAIMNAFSLKSPIRYRYLKEQNMFRAMTRGRHEYILTLPRLLNTTQTTTATLKMPTTEEKDTSLDKSKTQKPEIDNEQVRQESSRAACAAMEAQKKADELKKAAAGAGDADERQKLMEQAIEAQIEAESFGKTAKYMRSGAFQGLAMGTGLGAAPGVTLGAITGTLVGSVSSTLLGSIGGGIGAAAGAINGPFWDLSKFAGKGIRKITGDLPSWAASDEQKKALEKMLDQVNDEEMPDKQDLKGFVEDGGGADMDEGWLKKMKSCLPSLPSLTSSSGAKQQDNDAEKKSSDSSDEKQESALKTNDQPRKKPRKLEIKSHSEQELSEKSTEQKQKKPRKLETRSKHVEHEARESEETKAQPPK*

>jgi|Lepmu1|8287|jgi|Lepmu1|8287|Lema_T031810.1:1-85

MADERPTGLKANKGIELLTFGTPNGWKASILLEELKEAYGKDYTFQSINISQNIQKEPWFTTLGPNGRIPVIVDHDQGGFAVQEGAGKFQASNSIPTKAELLIDHPL*

>jgi|Lepmu1|3158|jgi|Lepmu1|3158|Lema_T096250.1:3-217

MVLTIHHLGLSQSERILLLLEEMAIPYTLTKHTRDPTMAPASLKQLPGNLTGQAPLMQDPDTGITLVESGAICDYILAKYKHEAKTKMSREYGEQGYADYVYFFHFANATLQPVMGQVMLLGLVKAPQDHVMVRYATNNMHQSLQILDDHLGHKKWLAGDDFTAADCMMIYSLTTKRYYGPLVSYAKYPNMVRYLKDVGERPAYKRAMEKGDPEMQVLLGAEPPEQSLVEVGGVTSDIWKKKT*

>jgi|Lepmu1|9560|jgi|Lepmu1|9560|Lema_T043790.1:1-207

MSSIKPIKIYGQHGPNPPKIAMLAKELNLPHEIEPISFPDLKKPEFLAINPNGRMPAIYDPNTDLTLWESGAIIEYLVDRYDEQRKLSFEPGSKETWLARQWLYFQVSGQGPYFGQAVWFTKYHSEQLPSAQNRYYQEIKRVTSVLESHLKQQPKGSDGPWLVGGKFSYADLAFVPWQLGAMKMLADKVDLSEFTEVKGWIERMMKKETISGTMAAAVPT*

>jgi|Lepmu1|4089|jgi|Lepmu1|4089|Lema_T105290.1:7-112

MSNPENPIRFFDIASGPPRRTYAPNPWKTRYALNAKQLAYKTEWVELPDVKKVRQQHNVAPVRKMPDGSDFYTLPMIHDGATDTYIGDSFDIAVYLDTQYPDHGLRLFPPASIGVHRVFNAHVDALFTRHIKIASAGLPLNPETAELTKSDFASRFGLGSWEDLVVRGEEREKILQAFEADMGEFATLYRYDDEGPFLEGRTVSYADMVVGGWLGMLKETLPEWGRVCEWQGGRWKRLHEALAPWAEIK*

>jgi|Lepmu1|6382|jgi|Lepmu1|6382|Lema_T113270.1:151-195

MATVNRFCTVVTASDRMVPSLGFPPRTVALSRGSFDGTHWSQFNTRIIGLSYYFQLRFEVLQFKIALSTFNQGNDRPRPFVDNHDSLLLSESHRSPAGLDSATAKQSFVTPQSLRVLKTKSTSIGYHTSLKASISLETIGPKLSRPTPTTMPQITLWFLQASRSIRTAWLLEELGLDHDLKFSERANQKAPEDFKIN*

>jgi|Lepmu1|6381|jgi|Lepmu1|6381|Lema_T113260.1:6-108

MATFADTYDKSDKLLLHDLEERIKVRQWARASEATFLLHALAILYARWNLPKGVHATTVEALESGISINLQKALSWLETELSLASGRFTCGDQITGADIVMQFSADFILARERAVEKAGYTLKLHPNVFFQSYLPSTLVRDANPTCKILVPLSQSLSLFEKIDGVTGYEEDENTIEAAANMAQRMLLRPTGLLLARRPQYAYRSFSTVLDTPIDPRTQQATPPARLTSVFEDALNASGPRTNWTKEEISEVYNTSLIDLTFAASTVHRRFHDPAAIQMCTLFNIKTGGCSEDCSYCAQSSKYDTGLKATKMSSVDSVLAAARIAKENGSSRFCMGAAWRDMRGRKTNLRNIKEMIKGVRGMGMEACVTLGMVDAAQAKELKDAGLTAYNHNVDTSREHYPSVITTRTYDERLNTIKNVQEAGIHVCTGGILGLGEKARDHVGLIHTVATLPAHPESFPVNALVPIKGTPLGDTQAISFDAILRTIATARLVMPKTIIRLAAGRHTMREEKQILCFQAGANAVFTGEKMLTTACNGWEEDKAMFGRWGLRPMKMEETIGEMRKVPGQDEAEAVVAAAEAEATVQALA*

>jgi|Lepmu1|2880|jgi|Lepmu1|2880|Lema_T081810.1:2229-2408

MHITKDIDTIFHRSLEGLTGDDHSPESRRDFPMSQSSGCRNGTDATVCHIFERIASQFPESVAAEDGGRNITYGELHYASNHLANHLSQIGIQSGQKIVIISNRSLEMIVALLGIMKSGACVVPIDFETWSQDRIQTTLETTQCRYAISTKCIEIPNQELILFQEGDLQHVLDNRRDQPASFSTRGFQLPSADDLAYTIFTSGTTSKPKGVMVPHSAIAHYVQQVSDEAPFNLNVQASSRVLLVFSVAFDACLGVVLSTICNGGTLILATSMNFATVATTCTILPLTPTILSTLRPGAEYDSIKSIFLGGESPSPNLLRPWLNGERRIFNCYGPTETTCTSLIKEVLPDEPNHLRYTVAGSSVVLLDGNLREVSEGEIAISGPGLAVGYFNNQALTAEKFIVYKGVRHYLTGDYGRKTSFGIDFLGRKDRVVKNRGFLINLEAEVEAVITNMKLANSAAALMHEGRLIMFVTPETIDVSSLRSRLLEIRDSFLVPDRIYAICSFPITSNGKVDLASLRQLLQEEKFTGVATHQSSPSSNLYVVLEGFSKVLGLPPSALCGSSSFLDNGGNSLSAVSLASHLRERGLSITVREIFESDTAQRICDTLSATILSTSDSEEADLESLRENVVRAGYPLTPRMEVAYMTAIQVNMIQSTIKMPSMNYIQLSITFDLSSGLFKPEVFRRAWEIIVQRHSILRATFIPALEATVIAADPTMDWREQLVDSSEWDSAVADAREKILCSMAPLDAEYLKPRSIFRLITEPKSRTEFIWTIHHSLVDGWSIAVIMRDLQCILSQEELPKVAQFTSVATVQKALAQRSLSRGKQQSWEEKMQNYIPAPRLRLPKPQGWARAARAERRQLLGVHRSQVQRFVQEYRVSDASIFLASWALVLSKYLSTDRVLFGVVLSGRNLPMAAVDQVVGPLLDTVPFPVNTTSTQSTAEFLRTIHGTLHEMNESPWEMKLQKSSMGPESLETLVALQYDLPDSTWNVDPKTWPSPQSMKHNETTELPLHILIDMQNGGDLEARYLYDCSHFEAAMIDQMLSHFSNMLKAILMHPTVELVKSSMMNQLEINDLLYSSPHMHDAYDGPQSLKQAFEEVVDTWPDAIAVESVSDSISYKELDHRSSAISNALLPLVGPGQIVGILSDGSVSWITAILAVLKAGAAYCPIDIALPEERIKVMLRESRCSLLLCTTEDLCELWANHSDLTCFSIGRLLSETLQTPERLPERCSPHDPAAVIFTSGSTGVPKGILLEHIGILSLLDFPNARLRSGPGRRNAQFLSLGFDCCVNEVFATLCYGATLVLRDPLDPVQHIKRVHATMCTPSFLATLDVNDFPNLELIALAGEPVPQKLVDTWGHNRVLLNVYSPSECTISTVYPQLYPGVQVTLGSPVPRQAIYILDKDLNPVPVGVPGEICISGIQVTRGYLNRPEETLVKFLPNPFQKGWRLYRSGDLGRLTNSHEIEYIGRIDNQVKVRGFRIELEEIESTIAALNPEVRQAAVIVVNDVLIGFVTPSSLDTLAIQAIISRHLPSYCRPSYFVALDNMPMSSNQKIDRKKLVSMKAERNHFTKVPIEGTTERIIQEIWKDLIPELGEVSALDNFLQIGGHSLLQARLTRQLGMALGNRIPLRIVIQNPVLRDLALAIDKHILDGGSEDISRGQPEQNTVLSHLEEEMYTVHMLSSEPSAWNIPYIARLTGPLNLAAFEASWNNIIRSNSILRARYQIKDGILTRSISTSISPVTRRYCKVTDDALLDIVNRAFDLANDQPIRLDLCLDRPTMSYVVLNMSHMIGDRSTMGEILRLLEEEYAQMILNDNFNLHEPLSESLPYSVWTAMRRKREVDAGLTHVLQKSLNPSLINPPLFGTFKQELACSAHRDKRIEGDLFSSLKNLRGRFKASGHQLAIAAVGLTLHRLSHREDFIIAAPIEDRTEAGTENMFGLFLDRLLIPLRFNLHSPHSADDLIHMVKSASEQAMANYIPFADLKNVLGMVGKSHSLCEIMVTYHASDLQGPNLTGVDALGIPVQPKGVKFPLMLEFSEFPESIGIDLAYDSHAIDNATMDEFEVQLMAAFRYLADETCSSTCTTYPPRLFPLIWSQKDTNTVAPISEDQEMIDLVREAMAECVGLNRCDISCSRSFFELGGSSVDCLRLQDRLIKSGVSVSLSSIIHLQTAELIAGAMENVNNRCIHRFTMTERPKDIPEDKLVLYVVKATPTSTANTVKPLIVMNELSIDHEIYVVPSPTRDEWFHQINPHKMVPAIESAETRDGKRLNIWESTSCLTYLTDAYDHEGLWKGSDLWERTQVNNWLTLHTAALGATGKYWLYFSAIHPEKIPAVIEKLANNIKVQYDILERRLSEKGQKYIALPDRPTIADVANLPFVTEELALKAGLRLGDWPNLQAWSEKMLARPSVQKALSQVQTFGHD*

>jgi|Lepmu1|10063|jgi|Lepmu1|10063|Lema_T053920.1:33-205

MSPIPTLYSAPESGNSYKARLLIALLKLDVRIVDVDLEHTEWFLKINPRGEVPVLVDGEVTLGDSSAVLVWLAGKAGSRFWSGDVGEQAGIVQWLAFANSWIQFGVFTNRAILSYNGPYNGLGTNASWTAEQIQTFLQEGAVRGNKSLAILQQHLEGEDWLVLGRPTIADVSVFVYVALAPMGDISLEPYPAVGRWIGRIKGLEGFVGIVGLEDPMVRR*

>jgi|Lepmu1|8603|Lema_T034220.1

MSALTWRLALRSKHIVRPLISSTRLFRRTLSTTTTTTSATQPKETAMDKKPSPDTKPGSILAWADQKTGE

FKRQTSTFRNFISRSAGAEFPPEANRYHLYVSYACPWAHRTLIVRALKGLQDIISYSSVHWHMGPKGWRF

ATADEVDSLPGHTIPDPCHTDTTHIRDIYFAQDPDYSGRFTVPVLFDKTTGRIVSNESAEIIRMLYVEFD

GLVGEAYRGVELLPEGLKGEIEEMNGWVYEGVNNGVYRAGFATTQEAYTKAVTQLFDSLDRLESHLASSS

TPYLLSSPHVTETDIRLFTTIIRFDPVYVQHFKCNIRDIRSGYPCLHRWMRHLYWDYPAFKSTTQFEHIK

NHYTKSHGQINPFSITPLGPVPDVLPKEDEVPAVKAVVGGK*

>jgi|Lepmu1|1383|Lema_T074080.1

MTIQIYCDPCTVNSRKVLAGLKQMNADYNHVFINYVTGEHKSEEFAKINPLKTVYDIRHQTSNAILQYAA

DKSGAESMYPKDLKRRADVNRWLFWEASSWFPACYVYLMENAVKSLMGGEPDQKAIDAASAKFHTCASVL

EARLSKNKWLTGDDVTIADIAVAAAMPVPQAMMLPLENYPNVKRWLAEEVQQLQSWKNTQGAVDKALLPN

QSATVKVLIVGLLAGVK*

>jgi|Lepmu1|6383|Lema_T113280.1

MTTPPEKPILFHYPQSIYSHRVLWYIWLRGITYDESIQPPIMPRPLLSSLSLTYRKIPLLAIGKDIYHDS

RLIISKLEQRYPNSTLTPSTPTELGIQKLLENWTVDGGIFAAAVKLMPYWNSESLLQNKAFLDDRQTLMG

GRRMSAESMEKGRPEGLAALRVAFDMLERTFFADGREWVLGSTGPTVADVDAVWPFEWLVWDRGMEGSLR

GGLCGEEEFPKVHAWIRRFMGLAEQRKRDCEKPKRLGAEEVTQRLVQAKGEPEALDFEDNNTLGLRKGDS

VDVYPSDYGQSGKSTGILLGLSTNEVVIQNDENLHLHFPRWNFTIKRISTMTIPKTINSKTPKLTLLYHP

FSPYTRKVYMHAQELNLHTHLTLQKVVVAPINIPGWSDDNAAVAVYNPMGKIPCLVTEDVPDGLFDSRII

CEYLSDLAGQQTQAKKKQDTRYWQLHTLHAAADGIMDAAVLITYEYRIRKERGLYFKEWVEGQKTKITRI

LDRFENAVRMGVLRVPGKGAASPDEIAVAVALALAKGMGELGIEWNRGRSGLARWMEVWEKRRSFLDTPP

TRDWVEEMEISKI*

>jgi|Lepmu1|4675|Lema_T107000.1

MLSSHQTVPETRRQICKSTGLGELLSLLHFAMSCHVMRAAQCSTPQLVLPFLGRRVNGEVVAKTNIPPYP

NGYTSPTNGLGGVGVGIGIGFRRRYWVWVSTAILGLGFDDAILPLGFNDMPSHTHDPSSSPRRSRTLRTH

QTQRDESPISSMSHRSPTASRDAQPLSPAKGKSGDMNSTTSSTATTSTTSIFTVPAPIKHLFDKFPLQTY

AANGLPQRAPRQRDVHVLYVFAGEEGRSFHPACLKWEAYLRFNKIPFRIARSNNHASPSGSLPFLLPAQT

DGKPQSQPVPSAKLQRWAMLNSGMEGGIQEPGDIRYEAYLSLIEHRIRRAWLYTIYLSPPNSPPSTPLYI

LPTTTNPLVRLSLAHSLRQAATSQLLLHTPTIHPSTLLAQADEAFAALDALLGQDSWFFGAGRPGLLDAS

VFSYTDLVMRGVGEGGLGGCVRGRGGLVAHWERCLGWGWGDGG*

>jgi|Lepmu1|8707|Lema_T035260.1

MASTTLDESLVSYLKAHAPNNAGAETDAVKASHALFPQVTYTDAEKTELGQWLHAASHIASSADDAAKAA

ERLSSLNTHLASRTTLLGAKPSVADIAIYQKLAPVVSQWSAEERTGEQGYHHIVRHVDFVQNSPIFGLKV

DDKLNIDQDAVVFKIKPVDAKAEKERKKKEKEAAAARAAASGATPTTLTGEQGGQASKSKKDKAQDKVQA

AGEAIASTVAGKPTAGGPPEGAPTKKKEKKDKQPKPQKAAPVEKPLSPALIDLRVGHILKAETHPNADSL

FVSTIACGDAPGTENTSEYEGQVVRTVCSGLNGLIPLAEMQNRKIVAVCNLKPVTMRGVKSCAMVLAASP

KLAPGEVDAHKGPVELVEPPPDSKAGDRVYFDGWQGDPEPVLNPKKKIWETLQPGFTTTQEGEVGFDVAV

VPQLAGEEPEKKVGVARLRTKDGVCSVPSLKDAVVR*

>jgi|Lepmu1|8899|Lema_T037180.1

MSSLMDLVAQNQPKFQTHQALLIIGMQNDFLLSDGRLPVNIKKGFLERIQTLIPKFRELNGNVIWVQTLY

EADRISSDPNTGEGDALVVGGLVDGDESSTEGGDEEPATPVKEAAKDVPPAQSRSSKHKQRALDLLKRVS

ARRKNIPQEVAKATAEEDDELFLLKSEKKTPACVPNTLGAEFADVIARQFELPADSVIRTTNYSAFQGTN

LLMTLRARLVTELYICGCITNVSVLATVIDAARHGVKINVIQDCLGYRKQTRHELALKRMDEFFDAYLVN

SEEIMARQPPLPQPAPSSPSNGSKNGSRGNEKSCDRTSGRLSQIEETRPTSRTASPRSPTAKVLTGKQRT

LSLVSVAENRKAAQNTGATTTNPTTEVQIEPSDKDFADMLVKGATVPGTQENEAEPEKPKLVQTKIRMRS

KEKRKKKREKEREEAPSNDKAETGGADGELSVPAANGSPIDSPTTTTAKSPQATTDSQRTSWIAKAGSVM

DLREKGNRPQSLKSAASVPVLSNKNEETDRNRLSDLSGRVRLSLSRAPKSESSEPKKSAGTSSTKGPITL

ASTKKGEKQADQSASSLKEESPSATPAPMAETSVKSVDVEESRQGSSILSTPTGTPKVKGSKLPSLATLP

VLGPDDTIGEGDSHIIYDFFPPTLCDPIGSSYPLKDHIFKQLFNEVQWQRMLHQQGEVPRLVCCQGAFGD

DGSMPVYRHPSDQTLPLLHFSPKVQLIRRRAEKIVGHSLNHVLIQLYRSGSDYISEHSDKTLDLVAGSSI

VNVSFGAQRTMRLRTKRASIHPTATPNSSPTNPSDSTKERITQRIPLPHNSLFTLGLGTNASFLHGIMPD

KRAPCDRSPAELDYDGIRISLTFRHVGTFIDASETIIWGRGATSKQQRDAADVINGDAEESEKLIRAFGK

ENAGLAQGKDAWQDCYASGSDVLHLRSVPQGHALPLLFLSGNEIEDKQVQICLAEAKLNVTVIPAPALDS

TTYDNGTRTVLFRDTDPHHTEVLSPSSILPYIDRYHPLDTSPHSRPCTARAHALLHTINALLTAHRASHT

CNLAIHLATLEEQLSTHSGPFIAGPKFSFADAAVWPVLHALARAGADRAEEGGDGMPGAGFLGFPCLEEY

YRGTWRRKASVRKVEEGLVEVGREGEGEDVGAGMEGREGEGGVEDKAKAKAV*

>jgi|Botci1|6061|jgi|Botci1|6061|BC1T_00939:1-415

MAFGTLYTYPGNPRSTAIRAVAKANNIELDIVETEPAKGVSEDYLKINKLGKVPTFVGADGFTLHEAIAIAIYVTSQNEKTTLLGKTKQDYASILKWMSFFNSEVLTSLGGWFRPLMGRDPYNKKNVDDSIKATAKCISVIEEHLLNNTYLAGERITLADLFAAGIISRGFQFFFDKKWRAENPNTARWYATVYNQPIYSAVVEPLALIDEPTLKNQAPKKAEAPKAEKPKKEAAKPKAKEVEEEEEEAPAAPKAKHPLESLAKPTIPLDEWKRQYSNQETPDALKWFWENFNAEEYSLWTVDYKYNDELTQVFMTSNLIGGFFARLEASRKYIFGCASVYGTANDSIIKGAFVIRGQEALPAFDVAPDYESYEFTKLDASKPEDREFVNEQWTWEKPLVVNGKEYPWADGKVFK

>jgi|Botci1|716|jgi|Botci1|716|BC1T_03423:296-550

MDQVNSGLHQQIPTTYRQLQPQAEQALQHGQPHLHHHTLTSQAGLDLSGLVQDDNNSVYHPDLRSLQDPNAGHPIAHQYPSPIPFERNNGHPQMHVQNPGIQNTGLQNSIQNNGSPHTPQQQHPGPGQFGILTAGPSLHHNPIGRLQQGLPQDLQQDDNLFGTPDETDQKSIGHHSHKIVPNPPDLAAWREKLFNVNEMITLSEEEYNTYFPHVDNVYSHRSTQKYKRKPFVSHYWDCRLKGQRYYEIQRVNGNGGNGKGDGVAGPHKHDLARSDEIKKNSILRFLQKREKEDKKTQKTYHKRASGLALSTVKKHTKENDLKLFGSCFCPFVQRVWIALEAKGIQYQYIEVDPYKKPQSLLEVNPRGLVPAIRHGDWGCGESTVLMEYIEDLQTGPPLFPQDARAKAHSRLWADHMDRKIVPTFYALLQSQNYEKQEELTAKLRDEISQIVDVCDPQGPFFLGPTLTYTDVHFAPWILRCRRVLKHYRDWQDPQPGSRWAIWFDAIENNEFVKATTSADELYIDSYERYAMNRPGTSELADAVNGGFNLP

>jgi|Botci1|12103|jgi|Botci1|12103|BC1T_09645:10-274

MTSSAAPNRPSGLLATSGIELLTFGTPNGHKISILLEELKAAYGKQYTYQSINIMENIQKEEWFTKFSPNGRIPAIVDHDRNGFAVFEGAAILAYLTRNYDVERRFSFEDEDDRSRVEQWVAWQHGGLGPMQGQANHFYRLAKERIPYPTQRYVGETERLYGVLDAHLADREYIVGPGKGKYSIADIANFSWVNVAYFAGVDLAKFTNLESWWKRINERDAVKKGTSVPSESRLINEAYKKRVEDDQEFREGEEKLRKLGEEAKVQYGYKYSSP

>jgi|Botci1|2622|jgi|Botci1|2622|BC1T_03433:6-264

MSEQKITHWVALNDKTGEFKRGQSQFRNFIKKGGEFPPEKGRYHLYVSYACPWAHRALIVRKLKGLEDIIPYTSVHWHMGEKGWKFATPDDDVTGENVTASPVEAHKEFTHLRDIYFQVDPEYTGRFTDPTLYDFDDIIAPEYKNVDLFPANLQKEIEATNEWTYNDINNGVYRSGFATKQEAYEKAVIQLFASLDRVEKHLSESEGPYYYGKNITEADVRLFTTIIRFDVVYVQHFKINPFSIAPVGPEPPILREDEEVPAVKWALSLRK

>jgi|Botci1|13044|jgi|Botci1|13044|BC1T_09470:80-255

MAWVGNSPQESDPYPGGNWVIIFSFPNILHQVYFRSQRKDFKGGDSPCLFCGTRRQNKIVCKESLEILRNLNTGFDSILDDEYKNLNFYPDNLAAEIDEMGEWIQSDINTGVYKAGFAPNQETYDKNVVPLFKALNRIEEVIQKNGGPYVLGSEMTELDLRLYPTICRFDAVYVQHFKCNLGTIRHDYPVLNAWLKHLYWEVKGFKESTDFKHIKENYTKSHADINPKAITPMGPIPNIERGVDHDWSKLVAGKVDLD

>jgi|Botci1|4596|jgi|Botci1|4596|BC1T_15218:8-245

MSSPASVLNQSRSQRILWLLEELKVPYELEIIHRLPSKHAPPELKKVHALGKSPVLTILPVGATEPVVLAESAFITEYLLDHFTHGSTLLPTRWKPGQENTLGGETEEWTRFRYYMHYAEGSLMPPLLVALIMSMINSPNLPFFIRPITGMITSKVHESFLEPNFSTHFTFLNEQIQSSPNGGKYLCGEHLTGADILMSFPLVAAKQRAGLTEDKYPELFAYTERLENEAGYKRAVEKIVDIEGEFKAI

>jgi|Botci1|5423|jgi|Botci1|5423|BC1T_04672:4-185

MSCLIPVFLGGINHGSGNKPPWTLPAKAAYGKLDSARTTAYHGLPNLQAPEFFPPVTLLPQRALCFIKSRYSVEVFEKTYLEIFHALWVPPQKNITIPEILKEFLNALGTFDEKEVEEIMQKATEKEWKDKLLENTKVALEQGAFGAPWMWVRNAEGKEEPFFGSDRFHFMWMFLGVEFRDLEIVRGATVGGEGKAKL

>jgi|Botci1|16143|jgi|Botci1|16143|BC1T_11874:2-155

MRVPVLSLEGEIITEVPAIATAISSLAPELHLLGGTTMETIKVYEWMNWLSGTLHAHAFGGLLRPERLSDEKEALPGIEKKGMRNAQNCFDMIEGKLNGFYAVGGAFTVVDSYLFVFYRWGEGYGLNMKSVYPKYTALVENLVNRPSMQKVLELEKSQAKL

>jgi|Botci1|7475|jgi|Botci1|7475|BC1T_05597:14-253

MSAVHPDANLFPHATGLAAEMVKQYSKEEPVKLYAGWFCPFTQRVLLLLLEKRIPFQYIEVNPYQKPLSLLKLNPRGLIPTLSYEGKALYESTIICEFLEDAYPSHYPRLLTREPFERARLKIWTDHITSRVVPAFNRFLQYQSVSLKDPRVLAIRNKFLTSLYEFTQEMHPIGPYFTGKEPCIVDYVLAPWALRIWIFDYFKGGLHIEELGELTGRWKKWLEAIEKRKSIQMTLSATEYYLPIYERYADSVPYVKIEKSREEEDGVF

>jgi|Botci1|6817|jgi|Botci1|6817|BC1T_16073:5-233

MAASYELIYYTGVPGRGEHVRLILEEAGVEYKDTQSLTFDEARDNVVTWLAGGGHGNPSYFAPPLFKYGDLVISQTPNILLYLGPKLGLAGSRENDLYRVNALALTALDLFSNEVHDTHHPIATMLPWEDQKEESKRRSKEWVQNRLPKVLAYWQKVLENEDRRPGPWLLGDTFTYADLVLFQTLDGTHYAFPKAMKQAREAGKYEKVFKLYEDVKARPNIAAYLASDRRQKYQDWGVYRHYDDNDVVAE

>jgi|Botci1|8327|jgi|Botci1|8327|BC1T_01279:20-234

MSSSFYLEGTPDEVKNAKGLHLVTMSTPNGKKVQIMLEELKAAYGTDYTHTLIHISTNEQKKDWFLKLNPNGRIPILIDNTKTPPFPVMETSAELLYLLKEFDSKDIFGFKDELERNECLQWMFFWHGSYQGQVNYFSKIAGEKNPGAIKRFKDETLRIFGVLEIRLSGKYTGEPRDYLAGNGKGKYSVADIGTWPWLNAWDFSGAITKEEMNAFPHLLKWIDRIAKRPAVQSGTSEKWQNW

>jgi|Botci1|7734|jgi|Botci1|7734|BC1T_05837:1-170

MVLELHIWGPSFGLPSIDAQCLATIAYMQQVIPRGQWSLVASSDPTLSPTSLDSLDIDTDDDKSRDQPSIIPESLRRGKQSVSSLLKASPETSAQIRLDALASDFFSAIGELKGNKKYLVSHEKLSSLDCLALGFMSLMLYPQLPQPWLQKTMRKKFPDLVQWVEELKDEAWNGVVGLDDALLTKSGNSGNANAVQQKKGSLPWQAPEARGILNIGGVFVASLADSLPIVGQQRKDNRMRREERR

>jgi|Botci1|902|jgi|Botci1|902|BC1T_12605:7-132

MGSHPAPKIILYTSYACPWAHRSQIALAELGLEFETVIIDLTVPRTPEYLAINPRGLVPALSYNGEILTESGVISQFLVDSHPSHLEKTSREEGGALQRARYNFFIETYFSKVAPHVVKAGIAKTWKKENKLPPML

>jgi|Botci1|1123|jgi|Botci1|1123|BC1T_12830:2-184

MVARPKITLYVDTVSPFAYEAYWILRHDPAFSKCDVEYVPVFLGGIMKDVGNTPPINVKNKDKWIDRERLHWASLFNIPMSSTTPIGFPHMTLRVQRALCVLPFLFPEENRAQDVLCHCLDRFYELYWVEGKNITAPGFLEDVLKHVVGEEKMELVVKEIAGKGKEAVMGNNERAVREGAFGLPVSFLLVSFFGGVS

>jgi|Botci1|2666|jgi|Botci1|2666|BC1T_03477:26-170

MSPAGTQATSSELTLYDLASQDHCASWSHNALKTRLLLNYKEIPYKTTWIEYPDIEPTFKSLKVQPNASGIQYTIPTIQIPELGYVQDSSTIATLLEHRYPSPSLKIETSELSQVEDLIPKLQLSIRPLWLPLLAKGLLNDASRVYFEETRKERVGMDIYEYGEKESKREVLGRY

>jgi|Botci1|979|jgi|Botci1|979|BC1T_12370:9-177

MSKAPKDTSTNEQKKDWFLRLNPNGRIPIIIDNFQSPPFPLMETSAELLYLLRLDKNHQFGFIDEIEQSELLQWLFFWHGSGAPYQGNYRFFSRAEEQSNFAIDRFRKETYRVYGVLELQLSGKYTGQPKEFLAGKDKGKYSIADIGTWAWVKNWPGSGFTEEEMKEFPSLLQWIERIAERPAVKRGIGEKYIGSR

>jgi|Botci1|7404|jgi|Botci1|7404|BC1T_05526:13-220

MASVNKPGLPTFHHLNNSQSQRILWFLEELGIEYNLVCHTRVEGRAPPELKNVHFMGKAPVLVTSDNVPIAESSAILGYLIDTYDKDGRFVAQDKIRDESLSSFAGSTIGTISMIELIFDIVAQKSPWPVSILLGGVKSNVHKSFTGPEYATQFQYLEKELTDVWFNGKNLGRSDVMLSWPMDFLAAKKYVDFEKYPKILQWRKGIQERDAWKRAMEKGNGYELAEI

>jgi|Botci1|14357|jgi|Botci1|14357|BC1T_10922:1-116

MVTITLDADYGYVILAATSTFILNFWHGINTGTYRKAAKIDYPAAYAPSSRTDTAAHQFNCAQRAHANFTENHSIAVTAMLVAGLEFPRSAATLGAAWTVSRWVYMRGYSQGGVGGKGRYKGIWFWLFQMGLMGLCGFMGGRMVLEGRV

>jgi|Botci1|1619|jgi|Botci1|1619|BC1T_13299:22-144

MSLKPLTIYYAVLQDPNTGYKIWETGAIIEYLIDQYDTDQKLSYASGLQKFNLFHAEKLPSAQKRYVDEIHRVVGVLDGVLGQSEDGWLVGNKVTYADLAFVTWHTALAGIFSPPEFKDQWDITKYAHYKKWVEKMLDRPAVKKVLEEQQRLVAESS

>jgi|Botci1|14577|jgi|Botci1|14577|BC1T_10460:3-217

MSSLGTVYTYPDNPRTMKIQAAAAFNHKTIDLFPEFVFFQTNRTPEFLSDFPLGRVPAFKDATSSFHLFESDAIAQYAAESGPAANQLLGSNVKERATIRQWISFANDEIFNPMTTLIFWRSGFGPFEKGAEDGAMGRLVLLLGVLEGQLSKHMYVSGTEDISLADISVAASLYWGFDQIIDVEMRERFPQVVRWYERTIKHQHLSPFWGEQKFVEKRRDRENV

>jgi|Botci1|11779|jgi|Botci1|11779|BC1T_08056:1-225

MADNKPTLHHLDHSQSQRILWLLEELGVEYNVVYHFRKPADDPIAPFRSPESLKALGPYGKAPVLTTGAADGNRYIPESDAICTYLLRKFDTEDKFGLISGDWVRDEILNCFNLTTFVRSVYFILFIDLDFIRNGVINWFDGPELREILTILDGELTNAPEGGYFMGKNPGRADIMMEFPMSFVKHRNWVDPEKEFPRLDEWLKRVYDRPAWKRGLQKGNGSYDLNVFPKRAVTS

>jgi|Botci1|3121|jgi|Botci1|3121|BC1T_13691:9-221

MTDNTPTFHHLNNSQSQRIFWLLEELSIRYNLQSHTRNPATDPKAPFLAPPSLKAISNYGKAPLLVTGPKDGNRSIPECLAIATYLIRTFDSADTFGLREGDWIRDEMLLSVIMTELGQLTYAMLMLDFNFIANGAGPMGKLLDGPALRKVLGVLEKELKEGPEGGFFMGKNPGRADIMLEFPMTLIKQRGWVDIEKEFPALGEWLTRCYERPAWKRSIEKGNGYDLTTFPQKAHLQAQL

>jgi|Botci1|897|jgi|Botci1|897|BC1T_12600:2-104

MSGQGPYFGQAHWFTKMHSEKVDSVIDRFLAQIKRVLYVLDRHLKGKEWLVGDKCTYADLSFVMWNEGIPWIFGDRAGELEMEKDYPNFFAWHTRLMERPSVKKVFEDKAAAKSGELPL

>jgi|Botci1|13563|jgi|Botci1|13563|BC1T_10073:25-197

MGLKLYGMRQATCTQRVLTTLAEKGVDYELILVNLMAGEQKAPSYLEKQPFGKVPVLDDNGFLIYESRAICKYLARKYADKGTKLIPAEGDVKGYGSFEQACSIEQAYLDVETFGLCARGWGATSPEAVQKHLQTLDNNLAVYDQILSKQKYLAGDEITLADLYHLPHGTQALKYGFQDLLGKYPHVNKWWEGLQARDSWKEVVAAAA

>jgi|Botci1|7138|jgi|Botci1|7138|BC1T_05767:32-189

MPQTSVYGTESAANKAIGGIPTIHYFDFQSRGRGQVVRLFLIDAGAAFKDIRYTFEEWPEHKRNGKVAEMNPTRNLPVVEMPEGKILTQSYAIVRHWSRLLGAYDGKNEDEKYWADAICDIVVDWRTIFLSAFFSDNKEEDYPKHQQGNQKKYLNAIETLLKGSELSKRGPFIIGKEITYADMALYQVLHDESLTKDGRKGLKEYPRLVQLVDAVEDRPNIKRFLNSDAYLG

>jgi|Botci1|5493|BC1T_15497

MVKPLGSPISTPKKSKVVTGVLESVLKTREWLVGDKCTYADLCFIAWQRWAPRYGGEDIYKDYPHVEAWLERMKMRPAVKKIYADQDFAMTEAQKK

>jgi|Botci1|14357|BC1T_10922 MAPEG

MVTITLDADYGYVILAATSTFILNFWHGINTGTYRKAAKIDYPAAYAPSSRTDTAAHQFNCAQRAHANFTENHSIAVTAMLVAGLEFPRSAATLGAAWTVSRWVYMRGYSQGGVGGKGRYKGIWFWLFQMGLMGLCGFMGGRMVLEGRV

>AB05379

MSFGKLYSYSGNPRTTSLLAVAKENGLDIEFVDTEPAKGVSEDYLKLNKLGKVPTFEGADGFVLSECIAIAVYLASQNEKTSLLGKTKQDYATILRWMSFANTEVLSPLGGWFRPILGRDPYNKKSVDESQKAALKAVHVIEEHLLTHTYLVGERLTLADIFATSILARGFQYFFDKQWRDSNPNTTRWYETVYNQSSYSAVAPKLEFISEALKNVAPKKEGGEKKKEQPKAAPKPKQEEAEEEEEEAPPAPKPKHPLESLPKATFVLDDWKRKYSNEETREVALPWFWENANFEEYSIYKVDYKYNDELTLTFMTANLIGGFFTRLEASRKYLFGCCSVYGQSNDSIVTGAFIVRGQEALPAFDVAPDVESYEFTKLDPTKPEDKEFVNDQWSWDKPIEVNGKSYEWADGKVFK

>AB03533

MTDSTPLVFYDISSPIQPRSYAPNPSKARLALGFKQVPFKTKFVDILDIPTVRKGLDCPATRKLDDGTDFHTLPMLQDPCANNKVIGDSFDMANYLEDTFPNSGGCLFPPDSTRTGLDYESPNKDTMFFAPLTLNQGAKNEAYAKFNTHRLRTL

>AB00471

MDKAPQQDGKDGKGSITDWVNPSDKSGEFKRQTSVFRNWIQNQPDAEFPPEKGRYHLYVSYACPWAHRTLIVRHLKGLEDIITYNSVHWHMAEKGWRFATSDEKVPGNTTPDSVHDGYTHLRDIYFEQNPDYEGRFTVPTLYDRKAKKIVSNESADIIRMLYTEFDDLVEEKYRKVNLFPKDLQKDIEAMNDWVYNDVNNGVYKSGFATTEEAYTKAVTQLFKSLDCLEESLSKSSTPYLLSSPHVTEADIRLFTTIIRFDPVYVQHFKCNIRDIRSGYPLLHKWMRHLYWDYPAFKETTNFEHIKKHYTKSHGQINKFQITPIGPLPDILEKDDEVPSVKAVMEK

>AB09620

MSGNEIHKYGTDDGWHGVIEEGSEFPPEKDRYHLYIGLFCPFAHRPNLIRHLKHLQPYLPVSIVRPYPKGEPGWRFDASYPNATPDHLFNSQFMHQLYFRDDPAYKGKYSVPLLWDKKSNRIVSNESAEMLKWLPSAFTSAQENNDKAKDLDFYPQELRQTIDKISPWLQSLICVGVYKAGFAPTQEGYEQGVIPLFAALNQLEELIHSNGGPYILGTKLTELDLLAYPTIVRFDTVYSQHFKTNLGSIRHDYPILNNWLKNLYHNVEGFKESTDFKHIKENYTKGHVDINPLSITPLGPYPDVEEGYEEDWGKLKPGKVAHPRVLEAQSKL

>AB08641

MTQHTLRLFHSPGSCSTAAHVLLQESGLLFSTEIINVMKGFPAELLHLNPKGRIPFLHMDGETITEMPAIMTAIAQLVPEKKFLGNNNLETVRCYEWFNFLAGDLHGQGYFTLYRPHYFIDDETAYDKIREKGRAKIDRCYGIIEDKLKGVHAVGNAFSAVDAYLLPFYRWGVAMGFGMKEKYPKYTALVENLAGMESVRKACEAEGIDPVAGARPELLTICDIFTIG

>AB09016

MPTIPLWFLQASRCIRTAWLLEELGLDYEVKFSERVNSKAPEDFKTASGNPLGKFPTLQDGTLTVYESGAINEDAPEGLLEATERSMSVNVQNDMSWLETELSLSPGRFLCSDHVTAVDTMMQFSAELIVAKELGTQGKEFPNVNKWLDACGETESYERALKKTGYKL

>AB07396

MTDTNQGGDSSKKTYHKKATGNALATVKNHSKEDDLKLYGSAFCPFVQRVWISLEHKQIPYQYIEVDPYKKPQSLLDVNPRGLVPALRHGPTWSTHESTVIMEYLEDLQAGPHLLPPDAQTRATSRLWSDHINRNIIPWFYKLLQAQEANEQVSHAKELRDQIGKLVDVADPTGPFFLGPQISFVDVQVAPWIIRLRRVLGPYRGWPEAEEGSRWKRWIDAIEADRSVKMTTSSDELYLDSYERYAENRPGTSQLADAVNSGRGLP

>AB06439

MSSSTTETSPLTLISATPSPFARMNRIALTLKGIPFKLQNEIPWQSATETPKYNPLEKLPILLFADGRPPVYDSAHIQEYIVRKYADKGPKLITDVDLDLQIRQIVVLGAGCMDAIVIARFECRREKEKQSQLWLDRQNRKIDGAMRALDEMPLLTKPKLEPIFSKSNDFTSQGSGGDDCSTVTNLIVDILRMSGCAQGKEIPIRLVLGKDAHDVNWEKCEGTLRRLKEWE

>AB06060

MSANKKARTQPPYELLYWPGIPGRGEFVRLAFEAAGVSYKDVANEAEDGIKVVAALTDQTSTGSDGNPPTFAPPALRIPGEGKNGAALIIYQTPSILSYLGDKLGLSGADEAEKSWILSHTLTALDLNNEAHDTHHPVSVGKYYEDQKEESLKKATEFRENRIPKFLGYFERVLKGNEAQGQGKFLVGGSLSYADTTLWHVLSGLEFAFPKEMEARKKDYSLLFGTFYDSIKETKGVKEYLGSDRRKPFSMGVFRHYPELDRQ

>AB03487

MPSNWHTSKFDGFHGRISVTGPFKPEPDRYHLYIGLFCPFAHRANLAMELKGIKEHAKIGVSVVRPYSLETGEGRRSLLFNVAAHEPDFENNYPGATQDKIFGSVTLGDVYLKADPLFEGRASVPMLWDRKLGTIVNNESDDLLRQLQTVFDPILPKQLQAVNLYPTTMQKEIDSISPWMHRDLNMGVYRVGFASTQKPYEEKVVSLFSALKRAEELVASRGGPFVLGEELTILDIQLFATIVRFDPAYVQAFRCNLGDIRHDHPALNTWLKTLYWNTTGKTAPFQETTHFDHIKDFYFMNFPELNPRRITPVGPCPPVEPAP

>AB01209

MSSPKLIVHHLQVGQGERIPWLLEELNIPYELKLYKRSPLLSPPELQAVYPLGASPVLEDLTDPSNPVKIAESGAICDYIIHKYANGRLALEPQHKNFADYLYWLHFANGTLQPALFRRGMTRGMVGEQDPRYKANDARVRKALSHVDNRLQNNKWLAGEEFTAAECMAGWCFTTMRAFEPVDLTDYPGILAWLGRVGEREAYRRAMGKSDPDINLEQSLSAKGMPMNEMFVKAMALKP

>AB06492

MPTPDVELLTAGTPNGQKISIFLEELGIPYKTTSINLSKDVQKSSSFLKVNPNGRIPAVIDHTRNSYPVFESGAIFLYLAEHYDTDFKFSFQDSDEKMEMMQWLFFQNAGVGPMQGQANHFVRYAPEKIEYGMKRYQNETKRLYSVLEARLQDRDYLAGKGRGKYSIADITTFTWVRWAPWAGIELKEFPRLKTWCEVIEKRDAVQKGLLVPSGEDQIERLRKDPNVQDPFKEWVQKGQKEEAKKHGN

>AB04098

MSQQPDINLYTTQTPNGIKISITLEELGLPYKVHKIDISKNTQKEEWFLKINPNGRIPALTDTFNDGQIINLFESGSIMEYLVDRYDSEHKISFPKGTREWYAMKNWLYFQNAGVGPMQGQANHFTRYAPEHIEYGVKRYQNETRRLYSVLDKHLASDNKPYICGEKCTIADISHYGWVASAGWAGIDIDEFPSLKAWEERMTSRPGVEKGRHVPDPHSIKELLQDKDKMEKYAAESRKWIQGGMKQDAEKQK

>Ab03486

MAPITLYFLQTSRAIRSAFLLEALELEYQVEVFNREANGDTPDQFRKNVPVGRAPAIVDNGLTIVESAAIAEYLCEKYDKGHKLMPADIAERTQVRVWMSASEGTFLMHALSIMYGSNAAPDAAEQISKGLSPQVHRDFDWLESELKQSTGKYLVGDHLTAADTMMGFTVAFIFKMGLGTSGKKWPAVEAWLANIESTEAYQRAVKKTGHTLG

>A4D12-AbGST6

MPSSKVVLYDLPGQQGTAWSLNPWKTRMVLNYRKIDYTTEWVEFPDLAPKFKTLGIPPNPKDAPGYFADYSSPVIKHANGTYQMDSWPIAFELEKQHPSSSLHLDDPIVEKIRDHIPKLIGPLVPLLIPQIPMAVLNKPSADYFYETREK

MFGKPVDQVGKDANVEECWERTKAPAKEAGDLLRNNGGPYFLGQTVSYADFIFVSTLHFVKCISENLFKELMALDDAFPKIYEASKQWLERED

>A1F1-AbGST2

MTLIVHHLQRSQSERIVWLCEELGIDYELKIYKRDRATLGAPSDLKAIHPTGTAPVIQDGDVTMSECGAIMDYIIGKYANGALTIPSNAPNFSDYVFWYHWGIGTFQNATMTLINVRMAGVEENHPILQALNKKITNALDMMNRRLLEST

WLAGDNFTAADIYAVFIVTTMRLFTPFPLSGYSGIKRWLENVRNRPAYRKMIEKAEQNENRGEVPVFCDEAPRPMISY

>AbGST1

msnqqgakitvhwlnksrgqrivwlleelgleydiavykrdknkragldlkavhplgksptvairpansekdivitesetimeyicdhfgkhliparypegqegvlgdeteewmrykflmdytegslftvlilalvtgniksapvpfflk

pitngvaskidsgfvnpelkthfdfledylvkspskgeffcsdkltaadimihfglegaaqrvplsetsypklyeymrrlqkrdayknaakrveeasgetyvpfsdakl

>A2C10-AbGST5

MSDQEPTGLKADKGIELLTFGTPNGWKASILLEELKDAYGKDYTWQAINISKNTQKEPWFTKLGPNGRIPVIVDHDQGGFAVQEGLAILTYLTRHYDPEHKFSFSDPLDVSRAEQWMAWQHGGLGPMQGQANHFNRFAKERIAYGMQRYT

GETERLAGVLDAALAKSDYLVGNKFSIADIASFGWIHMLRISGVDLDSFPNLKKWWERVLARPAVQRGLEIPSKSGFGNDTYVQKMKEDPEFAEKEHKLAEQIKTAKEQYGYKYSSP

>A2H5-AbGST4

MAIIQIPSEYGYVLAACVSTSFVGAWHAGRVGSFRKAAKIPYPYEYASYEQVQTASPQSRAAMLAFNATQRAHQNFNENHVTALGAMLITGLRHPVAAAVLGAVWSVNRVIYAIGYSRSGEDGGKGRYYGALGMVAHYVLMLMSGKAAYD

LVMG

>A2C1-AbGST3

MAQVNGNVPKITLYTNHRCPWAHRAHIVLKELGLPYEEVIIDLGKPREPWYLEINPVRSALYSSIEAPITLWQRGLVPAINFNGEIITESGVVATFLADAYPSHVLPASGSPEAALTRARINFFVDTWFSKAGSYFYKILMSGSEDEKAK

LSQEFVDVVGKEIEPLLKDANPFFGGSQKVTLAEALTAPFIIRTYAMAKHDMLPKSIASGLDALPNFSKWATQVNKQDSVTYIWDEVAVVDGTRKRIESQKAQAASK

>AB08862

MVLELHVWGPAFGLPSIEPECIATIGYCQRVIPKGEWSLVAEHNPTIGTTESLPILFDDDVATASGFEDIVAYLRNHPTVTNDPDINLSSRQRTDRTAFITFLQSTATPLIDLYLYVSAENYNTTTSSAYTAILPWYANYTVPPKRRDLARARTTHMGLSSLDVDTTAEAGFAPGRGTASSEYEAAKRAAGIPTESQPNAMSMGRGKGLGGLLGGQLYAARFRLDALSGELLDPLSDLLGKHDYLFRGEQSSSLDCLAFGYLSLLYYPPVPQAWLRETIQTKYPRIEAYIRSLHKELFRSEGVNSADVWSVATGAKKTPRSTCLPWAARPQTLASSMLAGAKEILGNVPGLSMLSRRNNVVVSEPLVVSERIQSELPSPLFVNTLLGVTAVFGISLASLAVHHRRSPREGELIFWALRPTNGLGEAGNILSVFANQLHDGALYAHS

>AB08827

MSRKITCYLDCVHLERNRKALAEHNVEIDIVPVFLGGINVGSGNKPPWTLEAKAKYSAYDNARAKKHFGLPNLEPPEFFPILSLLPQRALCAAKEAHREKFIDIFRDIFHAMWERGKDVSKPAILEEVLQQRLKEDEAKEVLAKANSQPYKQKLNDNTKQALDHGAFGCPWFFVRNSKGDEEPFFGSDRFHYIWEYLGLPWKDVELLPPGNAKAKI

>AB08219

MSQPKITLYVDVVSPFTYIAFHILKVFKQVKVTYVPILFGGLMKICENTPPLRIKNKDKWMNTERQRLSAYFNVPISQDNPPGFPINTLPIQRALASLSLSHPQHLEQTIALFYENFWAKWNDPTKPENLLAILTTALGSEEEARKVLERTKTEEVKKLLNGNTTKAFEDGAFGLPWFVATNAKGETEGYWGVDHMGLMCDHLGIERPESRGRRSQL

>jgi|Sacce1|3959|YLL060C yGTT2

MNGRGFLIYNGGEKMKQKMIIYDTPAGPYPARVRIALAEKNMLSSVQFVRINLWKGEHKKPEFLAKNYSGTVPVLELDDGTLIAECTAITEYIDALDGTPTLTGKTPLEKGVIHMMNKRAELELLDPVSVYFHHATPGLGPEVELYQNKEWGLRQRDKALHGMHYFDTVLRERPYVAGDSFSMADITVIAGLIFAAIVKLQVPEECEALRAWYKRMQQRPSVKKLLEIRSKSS*

>jgi|Sacce1|1829|YIR038C yGTT1

MSLPIIKVHWLDHSRAFRLLWLLDHLNLEYEIVPYKRDANFRAPPELKKIHPLGRSPLLEVQDRETGKKK

ILAESGFIFQYVLQHFDHSHVLMSEDADIADQINYYLFYVEGSLQPPLMIEFILSKVKDSGMPFPISYLA

RKVADKISQAYSSGEVKNQFDFVEGEISKNNGYLVDGKLSGADILMSFPLQMAFERKFAAPEDYPAISKW

LKTITSEESYAASKEKARALGSNF*

>jgi|Sacce1|2731|YGR154C yGto1

MSVSYKGTISKTHSVFKPEKGRYYIYGALGCPFTHRAILARSLKKLEPVLGLVLSHWQLDSKGARFLPAP

HRPEKYKERFFTATGGIASAKLDESEELGDVNNDSARLFVDGAFDPVENISRLSELYYLNDPKYPGTKFT

VPVLWDSKTRKIVNNESGDIIRILNSGVFDEFIQSEETNVIDLVPHDLIDEIDKNIKWVHPKINLGVYKV

GLAENGKIYETEVKTLFENLQKMECVLKENYKRLEEQFSGNKQKILAKYFVLGQRLTEADIRLYPSIIRF

DVVYVQHFKCNLKTIRDGFPYLHLWLINLYWNYAEFRFTTDFNHIKLFYIRMEVSRNKINQFGIVPLGPK

PDISRL*

>jgi|Sacce1|3917|YKR076W yGto2ECM4

MSKQWASGTNGAFKRQVSSFRETISKQHPIYKPAKGRYWLYVSLACPWAHRTLITRALKGLTSVIGCSVV

HWHLDEKGWRFLDMEKQLEDSEDFLEHWHDVAGGIRTAKEDSSKSFAEIKNDSQRFMVDATNEPHYGYKR

ISDLYYKSDPQYSARFTVPVLWDLETQTIVNNESSEIIRILNSSAFDEFVDDDHKKTDLVPAQLKTQIDD

FNSWVYDSINNGVYKTGFAEKAEVYESEVNNVFEHLDKVEKILSDKYSKLKAKYGEEDRQKILGEFFTVG

DQLTEADIRLYTTVIRFDPVYVQHFKCNFTSIRAGYPFIHLWVRNLYWNYDAFRYTTDFDHIKLHYTRSH

TRINPLGITPLGPKPDIRPL*

>jgi|Sacce1|4945|YMR251W yGTO3

MSEKSASNNKAEFKRQSSPFREIISADHPIYKPAKGRYWLYVALPCPWAQRTLITRALKGLAPIIGCSVA

HWHLDDKGWRFLEEGDGKTNERHWFDIAGGISSVNLNTSTPVANIPNNAHRLLVDGTDEPHYGYKRLSDF

YFKTKPDYKGRFTVPVLWDLETCTIVNNESSDIIGIMNSAAFDEFVGEEYRQVRLVPRSLEAQITEFNSW

VYDKINNGVYKAGFAECAEVYEREVTSLFQYLDKLENLLDKKYTDLEAEYGKNNKDKILDRYFAIGDTLT

EADVRLYPTIVRFDVVYHQHFKCNLATIRDDYSRIHTWLKNIYWRHEAFQRTTDFTHIKLGYTRSQPRVN

PIGITPLGPKPDIRPP*

>jgi|Sacce1|6309|YPL048W yEF1B2 TEF3

MSQGTLYANFRIRTWVPRGLVKALKLDVKVVTPDAAAEQFARDFPLKKVPAFVGPKGYKLTEAMAINYYL

VKLSQDDKMKTQLLGADDDLNAQAQIIRWQSLANSDLCIQIANTIVPLKGGAPYNKKSVDSAMDAVDKIV

DIFENRLKNYTYLATENISLADLVAASIFTRYFESLFGTEWRAQHPAIVRWFNTVRASPFLKDEYKDFKF

ADKPLSPPQKKKEKKAPAAAPAASKKKEEAKPAATETETSSKKPKHPLELLGKSTFVLDDWKRKYSNEDT

RPVALPWFWEHYNPEEYSLWKVTYKYNDELTLTFMSNNLVGGFFNRLSASTKYMFGCLVVYGENNNNGIV

GAVMVRGQDYVPAFDVAPDWESYDYAKLDPTNDDDKEFINNMWAWDKPVSVNGEPKEIVDGKVLK*

>jgi|Sacce1|3752|YKL081W yEF1B TEF4

MSQGTLYINRSPRNYASEALISYFKLDVKIVDLEQSSEFASLFPLKQAPAFLGPKGLKLTEALAIQFYLA

NQVADEKERARLLGSDVIEKSQILRWASLANSDVMSNIARPFLSFKGLIPYNKKDVDACFVKIDNLAAVF

DARLRDYTFVATENISLGDLHAAGSWAFGLATILGPEWRAKHPHLMRWFNTVAASPIVKTPFAEVKLAEK

ALTYTPPKKQKAEKPKAEKSKAEKKKDEAKPADDAAPAKKPKHPLEALGKSTFVLDDWKRKYSNDDTRPV

ALPWFWEHYNPEEYSIWKVGYKYNDELTLTFMSNNLVGGFFNRLSASTKYMFGCLVVYGENNNNGIVGAV

MVRGQDFAPAFDVAPDWESYEYTKLDPTKEEDKEFVNNMWAWDKPVVVNGEDKEIVDGKVLK*

>jgi|Sacce1|2781|YGR201C YEFB1

MSDGTLFTDLKERKLIRTIVPRGLVRSLKLDVKLADPSDAQQLYEREFPLRKYPTFVGPHDEWTLTEAMA

IDYYLIHLSSDKEAVRQLLGPEGDFKTRADILRWESLSNSDFLNEVCEVFFPLIGVKPYNATEFKAAREN

VDTIVSLYEKRLKKQQYLVCDDHETLADLISAAAFSLGFISFFDETWRSKHPEVTRWFNRVIKSRFFEGE

FESFKMCETEMQPIK*

>jgi|Sacce1|58|YAL025C yMAK16

MSDEIVWQVINQSFCSHRIKAPNGQNFCRNEYNVTGLCTRQSCPLANSKYATVKCDNGKLYLYMKTPERA

HTPAKLWERIKLSKNYTKALQQIDEHLLHWSKFFRHKCKQRFTKLTQVMITERRLALREEERHYVGVAPK

VKRREQNRERKALVAAKIEKAIEKELMDRLKSGAYGDKPLNVDEKVWKKIMGQMEEENSQDEEEDWDEEE

ESDDGEVEYVADDGEGEYVDVDDLEKWLADSDREASSASQSESDSESESDSDSDEENKNSAKRRKKGTSA

KTKRPKVEIEYEEEHEVQNAEQEVAQ*

>jgi|Sacce1|5145|YNL229C ure

MMNNNGNQVSNLSNALRQVNIGNRNSNTTTDQSNINFEFSTGVNNNNNNNSSSNNNNVQNNNSGRNGSQN

NDNENNIKNTLEQHRQQQQAFSDMSHVEYSRITKFFQEQPLEGYTLFSHRSAPNGFKVAIVLSELGFHYN

TIFLDFNLGEHRAPEFVSVNPNARVPALIDHGMDNLSIWESGAILLHLVNKYYKETGNPLLWSDDLADQS

QINAWLFFQTSGHAPMIGQALHFRYFHSQKIASAVERYTDEVRRVYGVVEMALAERREALVMELDTENAA

AYSAGTTPMSQSRFFDYPVWLVGDKLTIADLAFVPWNNVVDRIGINIKIEFPEVYKWTKHMMRRPAVIKA

LRGE*
